# Evidence for circulation of high-virulence HIV-1 subtype B variants in the United Kingdom

**DOI:** 10.1093/ve/veaf048

**Published:** 2025-05-20

**Authors:** Vinicius B Franceschi, Kieran O Drake, David F Bibby, Caroline A Sabin, David T Dunn, Jean L Mbisa, Erik M Volz

**Affiliations:** MRC Centre for Global Infectious Disease Analysis, Department of Infectious Disease Epidemiology, School of Public Health, Imperial College London, 90 Wood Lane, London W12 0BZ, United Kingdom; MRC Centre for Global Infectious Disease Analysis, Department of Infectious Disease Epidemiology, School of Public Health, Imperial College London, 90 Wood Lane, London W12 0BZ, United Kingdom; Antiviral Unit, Virus Reference Department, UK Health Security Agency, 61 Colindale Avenue, London NW9 5EQ, United Kingdom; Institute for Global Health, University College London, Rowland Hill Street, London NW3 2PF, United Kingdom; MRC Clinical Trials Unit at University College London, 90 High Holborn, London WC1V 6LJ, United Kingdom; Antiviral Unit, Virus Reference Department, UK Health Security Agency, 61 Colindale Avenue, London NW9 5EQ, United Kingdom; MRC Centre for Global Infectious Disease Analysis, Department of Infectious Disease Epidemiology, School of Public Health, Imperial College London, 90 Wood Lane, London W12 0BZ, United Kingdom

**Keywords:** HIV-1, virulence, CD4 lymphocyte count, viral load, molecular phylogenetics

## Abstract

The evolution of HIV-1 virulence has significant implications for epidemic control. Recent phylogenomic analyses identified low-prevalence HIV-1 variants exhibiting significant differences in disease progression. We analysed 40 888 partial HIV-1 pol sequences from the UK HIV Drug Resistance Database (UKRDB) across subtypes B, C, A1, and CRF02AG. We identified phylotypes with putative differences in transmission/phylogenetic patterns and assessed their virulence trends using pretreatment viral loads, CD4 cell counts, and four statistical methods. We classified three subtype B phylotypes—PT.B.40.UK, PT.B.69.UK, and PT.B.133.UK —as variants of interest (VOIs) due to significantly higher viral loads and/or accelerated CD4 decline. PT.B.40.UK and PT.B.69.UK exhibited higher viral loads, 4.93 log_10_ copies/ml (95% CI: 4.73–5.13) and 4.87 (4.65–5.10), representing 0.30–0.36 log_10_ copies/ml higher than the reference group (4.57; 4.55–4.59). Despite uncertainties in baseline CD4 counts, all three VOIs reached the clinically relevant threshold of 350 CD4 cells/mm^3^ significantly faster than the reference group (3.5 years, 3.1–3.9 years): 2.3 years (1.0–5.1) for PT.B.40.UK, 2.0 years (10.8 months–4.4 years) for PT.B.69.UK, and 1.8 years (10.8 months–3.6 years) for PT.B.133.UK. These VOIs and their closest relatives have been circulating in the UK for decades with limited international spread and did not exhibit unusually rapid growth rates. Although these findings suggest a heritable high-virulence HIV-1 phenotype, we did not find evidence that convergent genetic polymorphisms or switches in coreceptor usage explained these differences. The small fraction of HIV-1 subtype B variants in the UK evolving towards higher virulence is unlikely to pose a public health concern, given the ongoing decline in new HIV diagnoses following the widespread adoption of pre-exposure prophylaxis and targeted prevention campaigns. However, this study—alongside the detection of the VB variant in the Netherlands—demonstrates that more virulent variants are not rare and can emerge independently in multiple countries. Consequently, HIV-1 genomic surveillance remains crucial to monitor HIV-1 virulence and mitigate its healthcare impact.

## Introduction

HIV-1 evolution is characterized by an error-prone replication machinery, multilevel selective pressures leading to faster within- compared to between-host evolutionary rates (Lemey *et al*. 2006) and intermediate levels of virulence—the degree of sickness or damage caused by the virus to its host—which has been hypothesized to be near optimal for viral fitness in the absence of antiretroviral therapy (ART) (Fraser *et al*. 2007). Although differences in virulence across subtypes and Circulating Recombinant Forms (CRFs) have been reported (Taylor *et al*. 2008), the extent to which the aforementioned and other genetic determinants—including immune evasion, receptor binding efficiency, and tissue tropism (Wertheim 2022) —are shaping the evolution of virulence are still elusive. Most importantly, HIV-1 phenotypic traits such as viral loads (titre of viral particles in the blood) and CD4 counts (number of CD4^+^ T cells in the blood per unit volume) are heritable, i.e. their variation is partially attributable to virus genotype, being linked to transmissibility and evolving in response to different selective pressures (Fraser *et al*. 2014). The heritability of set-point viral load (SPVL), a more stable viral load measured during asymptomatic infection (typically between 6 and 24 months of infection), is estimated to be around 20%–30% (Fraser *et al*. 2014, Leventhal and Bonhoeffer 2016, Bartha *et al*. 2017, Blanquart *et al*. 2017, Bertels *et al*. 2018, Mitov and Stadler 2018). The heritability of CD4 cell decline is less well established, with estimates of 11% (95% CI: 0–19) (Blanquart *et al*. 2017) and 17% (95% CI: 5–30) (Bertels *et al*. 2018). Therefore, we expect that elevated viral loads and faster CD4 declines would be observed in more virulent variants in the absence of ART.

An HIV-1 variant (VB) with elevated virulence was recently detected in the Netherlands (Wymant *et al*. 2022) through comparative phylogenomic analyses of a large cohort of people living with HIV (PLWH) integrated with the aforementioned clinical markers (viral loads and CD4 counts). This discovery has demonstrated the potential existence of low-prevalence variants exhibiting substantial changes in transmissibility or pathogenicity and builds substantially on previous knowledge by indicating individual lineages that could be responsible for driving population-level shifts in HIV-1 virulence. Most importantly, this raises the question: what is the evolutionary pathway HIV is taking in different countries? Examples from three continents show potentially divergent scenarios: (i) the less virulent subtype A is outcompeting subtype D in Uganda (Blanquart *et al*. 2016), whereas (ii) a slow but detectable evolution towards higher virulence is observed in the USA (Wertheim *et al*. 2019).

Phylogenomic analyses have also been conducted in the past few years to elucidate how HIV spreads over time, between locations, and across risk groups (Lewis *et al*. 2008, Faria *et al*. 2014, Le Vu *et al*. 2019). More importantly, the way phylogenetic clusters are structured can provide valuable insights into the processes that contributed to their establishment, such as the action of natural selection. In this context, it is now possible to detect cryptic population structure in time-scaled phylogenies (Volz *et al*. 2020). This refers to the action of evolutionary and epidemiological forces that have led to distinct evolutionary patterns in different clades of the virus, but that are not necessarily associated with any visible metadata associated with molecular sequence data. Consequently, their existence must be inferred from phylogenetic patterns. Partitioning the HIV-1 phylogeny into such distinct clades, which we call ‘phylotypes’, provides a natural basis for comparative phylogenetic studies. We consider several methods for tree partitioning (Balaban *et al*. 2019, Tonkin-Hill *et al*. 2019, Volz *et al*. 2020), but our main results focus on the method ‘treestructure’ (Volz *et al*. 2020), which is premised on partitioning clades that show significant deviations from a neutral coalescent null hypothesis.

Here, we investigated the presence or absence of fast-growing and virulent HIV variants in the UK HIV Drug Resistance Database (UKRDB)—a UK central repository for routine resistance testing—using coalescent-based phylodynamic methods combined with PLWH-level sociodemographic and clinical data. We identified one subtype B phylotype with concomitant elevated viral loads and rapidly declining CD4 counts (PT.B.69.UK), one with remarkably higher viral loads (PT.B.40.UK) and one with pronounced faster CD4 decline (PT.B.133.UK). We designated these phylotypes as VOIs. We also demonstrated that these moderately virulent HIV-1 variants and their closest relatives have been circulating in the UK and worldwide (especially in North America) for the past 40 years.

## Methods

### Study population and sequence selection

We obtained partial HIV-1 pol sequences from the UKRDB, a UK central repository for routine resistance testing established in 2001. Sequences covering the whole protease (PR) and 75% of the reverse transcriptase (RT) genes (HXB2 nucleotide coordinates 2253–3554) are aligned and subtypes assigned using REGAv3 (de Oliveira *et al*. 2005). Clinical and demographic data from the UK Collaborative HIV Cohort (UK-CHIC) Study (UK Collaborative HIV Cohort Steering Committee 2004) and the national HIV and AIDS Reporting System (HARS) (https://www.gov.uk/guidance/hiv-surveillance-systems) are linked to genetic data from early 1991 to mid-2019 and records subsequently anonymized and delinked from clinical identifiers. The London Multicentre Research Ethics Committee gave ethical approval for nonconsented studies of anonymized data (MREC/01/2/10; 5 April 2001).

From a total of 98 803 PLWH and 145 094 sequences available, we selected the four most abundant subtypes for detailed analysis, namely, B (44 900 people, 45.4%), C (*n* = 23 119, 23.4%), A1 (*n* = 6322, 6.4%), and CRF02AG (*n* = 5171, 5.2%), (Table 1, Table S1). After alignment, the sequences were trimmed to a length of 995 nucleotides because sequencing coverage was consistently high in this region across the set of sequences. Only individuals with a sequence available while treatment naive were included, and the first sequence per PLWH with length > 900 nucleotides was selected. People with unknown risk groups were excluded from the analysis (Table 1, Tables S1 and S2). Risk groups refer to modes of HIV acquisition as recorded in HARS, based on self-reports from PLWHs. However, clinician judgement is applied to determine the most likely route of infection. A total of 18 871 (46.2%) of all analysed sequences (*n* = 40 888) were linked to UK-CHIC data, the majority of which (70%) originated from London, indicating an overrepresentation from this region. Additionally, all CD4 measurements (100.0%) were obtained from UK-CHIC.

**Table 1:** Number of PLWH per subtype included throughout the employed analytic workflow

	**HIV-1 subtypes**					
**Analytic step**	**B**	**C**	**A1**	**CRF02_AG**	**Others (*n* = 360)**	**Total**
All UKRDB participants	44 900 (45.4%)	23 119 (23.4)	6322 (6.4)	5171 (5.2)	19 291 (19.5)	98 803
After sequence and treatment-naive filters^a^	25 281 (56.3)	11 710 (50.7)	2844 (45.0)	2836 (54.8)	–^d^	42 671
Nonmolecular-clock outliers^a^	24 100 (95.3)	11 331 (96.8)	2714 (95.4)	2743 (96.7)	–	40 888
Viral load Bayesian joint analysis^a^^,^^b^	8789 (36.5)	2356 (20.8)	755 (27.8)	933 (34.0)	–	12 833
CD4 decline Bayesian random effect model^a^^,^^c^	8946 (37.1)	2098 (18.5)	700 (25.8)	619 (22.6)	–	12 363

^a^Percentages calculated respective to the same subtype (column) quantities on the immediate upper rows; for viral load and CD4, percentages are calculated from nonmolecular-clock outliers. Therefore, row percentages (except for the first) do not sum to 100%. ^b^This row presents the number of unique individuals used in the Bayesian joint viral load analysis. In the other viral load analysis (*t*-tests), phylotypes with <10 individuals with viral load information available were removed, leading to a smaller number of individuals considered (*n* = 3306). ^c^Only individuals with more than one CD4 measurement pretreatment were included. This row presents the number of unique individuals. The total number of measurements considered is 31 503, 6617, 2266, and 1969, respectively. ^d^The en dash symbol (–) means ‘not applicable’, as only the four more represented subtypes were used for subsequent analyses.

### Phylogenomic analyses

For each subtype, we aligned UK sequences against reference sequence outgroups (HXB2 [NC_001802] for subtypes C, A1 and CRF02AG; C.ET.86.ETH2220.U46016 for subtype B) using MAFFT v7.505 (Katoh and Standley 2013) to root the phylogenies. We reconstructed maximum likelihood (ML) phylogenetic trees using IQ-TREE v2.2.2.6 (Minh *et al*. 2020), employing the general time-reversible (GTR) model (Tavare 1986) with a FreeRate model for rate heterogeneity across sites and 1000 ultrafast bootstrap (UB) replicates to quantify branch support. For subtype B, we estimated time-scaled phylogenies using treedater v0.5.3 (Volz and Frost 2017) under a strict clock model (substitution rate: 1.15 × 10^−3^ subst./site/year, which is the median estimate from 10 independent replicates of 3000 sequences each) for computational feasibility. For subtypes C, A1, and CRF02AG, we used an uncorrelated additive relaxed clock model (Didelot *et al*. 2021). For evolutionary analyses, we also removed molecular-clock outliers (Table 1) using root-to-tip regression as implemented in treedater (tail probability = 0.0025 for subtype B and 0.05 for the other subtypes).

We further identified HIV-1 clades (henceforth called phylotypes and named according to the following convention: PT.subtype.id.country, e.g. PT.B.50.UK) with putative significant differences in evolutionary patterns using a coalescent null hypothesis for branching patterns as implemented by treestructure v0.3.1 (Volz *et al*. 2020). This method recursively tests the distribution of times of the most recent common ancestor (TMRCAs) in subclades within a phylogeny using a neutral coalescent null hypothesis and then partitions the tree where the null is rejected. This approach will detect clades where there are significant differences in natural selection, sampling, or transmission patterns, such as due to different behaviours and geographic structure (Volz *et al*. 2020). We retained clades of three minimum clade sizes (30, 50, and 100) and having at least 80% UB support (Table S4). We also explored other partitioning methods including fastbaps (Tonkin-Hill *et al*. 2019), ClusterPicker (Ragonnet-Cronin *et al*. 2013), and treecluster (Balaban *et al*. 2019). Fastbaps is an algorithm that assigns clusters based on hierarchical clustering, while ClusterPicker and treecluster groups sequences based on a genetic distance threshold. For fastbaps*,* we ran four options for the hyperprior of the Dirichlet process mixture model (*baps, symmetric, optimise.baps, optimise.symmetric*) and selected the output given by *optimise.symmetric* that better approximated the number of phylotypes detected by treestructure with the minimum size of 30 in order to provide a fair comparison (Table S4). For Cluster Picker*,* we varied the initial and main bootstrap support threshold, as well as the maximum genetic distance allowed within clusters, but >500 clusters were returned even for high distance thresholds (10%). We therefore excluded this method from the analysis. For treecluster, we tested two different methods (*max clade* and *avg clade*, which are, respectively, based on maximum or average pairwise distance between leaves in the cluster), 30 genetic distance thresholds equally spaced between 0.5% and 15% and a bootstrap support of 80% (Fig. S2). The *avg clade* method and genetic distance = 7.5% more closely approximated the number of phylotypes estimated by treestructure (Fig. S2 and Table S4).

Results are focused on the partitioning method and minimum cluster size value that explained more variance in viral loads, namely, treestructure with at least 30 sequences in each phylotype. In total, 154, 30, 12, and 11 phylotypes were assigned for subtypes B, C, A1, and CRF02AG, respectively (Table S4). In genomic analyses, paraphyletic phylotypes were resolved by tracing TMRCA of all sequences in the phylotype until a monophyletic group was achieved (Table S5). Moreover, the presence of large ‘backbone’ phylotypes for each subtype—which represents the largest (>50% of samples) and diverse ancestral type from which all of the other phylotypes were descended—alongside the analysed well-supported phylotypes (Fig. S1) allowed us to compare the clinical markers of each phylotype against such clusters that show absence of evidence of a putative difference in transmission dynamics (Table S5).

We separately estimated effective population sizes (Ne) and growth rates for each phylotype using mlesky v0.1.7 (Didelot *et al*. 2023) under a skygrid model (Gill *et al*. 2013) with fixed phylotype-specific ML trees and 50 grid points, and also including several variations in the precision parameter ranging from 2.5 to 100, which was optimized using cross-validation. Additionally, we computed logistic growth rates for each phylotype using a generalized additive model (GAM) with a binomial link to estimate the time-varying probability of sampling each VOI implemented in the mgcv v1.8 package (Wood 2011, Volz 2023).

To estimate the heritability of viral load, we used a Bayesian Phylogenetic Ornstein–Uhlenbeck Mixed Model (POUMM) as implemented in the POUMM R package v2.1.7 (Mitov and Stadler 2019). This model used a standard uniform distribution as an uninformed prior for the phylogenetic heritability (*H*^2^) parameter, 500 000 Markov chain Monte Carlo (MCMC) iterations, and three independent chains. An ML tree pruned to contain all subtype B sequences with cross-referenced pretreatment viral loads (*n* = 8789) and the respective mean viral loads per individual were provided as input.

### Linkage between genetic data and viral loads

As the times of infection were not known, we were unable to extract stable SPVLs during confirmable chronic infections and the following heuristic was used: (i) we extracted pre-treatment viral loads up to 2 years after diagnosis; (2) we filtered out measurements below 200 copies/ml (viral suppression) and above 10 000 000 copies (viral quantification assay precision limit). Viral loads were then converted to the log_10_ unit. Subsequently, we calculated the mean viral load per individual. Restricting to individuals with a viral load defined in this way resulted in *n* = 12 833 PLWH across the four investigated subtypes (Table 1). The National Health Service (NHS) laboratories providing these data use different commercial European Conformity (CE)–marked viral load assays. These assays are considered comparable, as they have passed the accreditation process and participate in an annual accredited External Quality Assessment (EQA) scheme.

To estimate the phylotype-level variation in viral loads, we first implemented a simple approach that tests differences in viral loads between each phylotype and its respective backbone phylotype (Table S6). For this purpose, we utilized a Welch’s *t*-test, which is more robust to unequal variances between groups when compared to the traditional Student’s *t*-test. We defined a one-sided test in R v4.1.3 to select only the phylotypes with higher viral load than the backbone. *P*-values were adjusted for multiple testing using the Bonferroni and the Benjamini–Hochberg (false discovery rate [FDR]) (Benjamini and Hochberg 1995) methods. An FDR-adjusted *P*-value ≤ .05 was considered statistically significant. For this test, phylotypes not containing at least 10 individuals with viral load measurements were removed.

Second, we used a Bayesian joint model (Table S7; Chris Wymant, personal communication) that uses all the data instead of performing pairwise comparisons against the backbone. It assumes that viral load measurements are normally distributed around a mean value that is itself normally distributed between phylotypes. Weakly informative prior distributions were designed using an empirical Bayesian approach in order to be conservative about detecting phylotypes with higher viral loads (Text S1).

We implemented this model in RStan v2.32.6 (Carpenter *et al*. 2017) using two independent chains of 4000 MCMC iterations each and assessing convergence based on R-hat < 1.01 and effective sample size (ESS) > 400. We calculated a two-tailed Bayesian *P*-value for each group with respect to the population-level mean, i.e. twice the posterior mass for the group effect being less than zero (for positive effects) or greater than zero (for negative effects). The precision of the *P*-value depends on the number of samples we extract from the posterior (iterations of the MCMC). Finally, we extracted median values and estimated 95% credible intervals for each parameter using the 2.5th and 97.5th percentiles of the posterior samples.

### Linkage between genetic data and CD4 counts

To investigate the rate of CD4 cell decline, we extracted data from individuals with more than one pretreatment CD4 measurement (Tables 1 and S2). We excluded CD4 measurements taken >1 month before the diagnosis date, which are probably related to earlier clinical investigations unconnected with an individual’s HIV infection. Additional filtering steps were carried out to avoid the inclusion of mis-recorded measurements. We removed outlying individuals by calculating *z*-scores from maximum-likelihood slopes and intercepts (from the random effects model described below) and filtering out intercept and slope values outside the range of −3 to 3. This threshold corresponds approximately to the 0.5th and 99.5th percentiles of the distributions of slopes and intercepts. Under the assumption of approximate normality, it excludes extreme outliers while retaining >99% of the data—representing a balance between robustness to erroneous values and avoiding overfitting to noise. We also removed measurements with >2000 cells/mm^3^, which are implausibly high. Limiting the sample to individuals whose CD4 count was defined in this manner resulted in *n* = 12 363 PLWH across the four investigated subtypes (Table 1).

We then built ML and Bayesian linear mixed-effects models to evaluate if any phylotypes were associated with faster CD4 decline. As previously observed (Wymant *et al*. 2022), square root transforming CD4 counts was not influential to the model, as there was a strong overlap of significant phylotypes resulting from the transformed and untransformed CD4 ML models. Therefore, results are presented using the untransformed scale.

Firstly, as a way of finding outlier phylotypes, we implemented an ML version of a mixed-effects model using the lme4 v1.1.30 (Bates *et al*. 2015) R package, providing a fast and convenient way to filter phylotypes before in-depth analysis. This model utilized a random effect on the intercept and slope for both phylotypes and individuals (distributed as a 2D joint normal allowing correlation) and fixed effects on other covariates such as age group (intercept), sex (intercept and slope), and risk group (slope). The reference category for fixed effects was defined as: men who have sex with men (MSM) in the backbone phylotype and belonging to the 30–39 age group. The likelihood used has each measurement normally distributed about its expected value with the same variance for every measurement in the dataset, with the expected value being an individual-specific linear function of time since the first CD4 measurement. This model assumes independence across phylotypes, normality of random effects and residuals, linearity between the dependent variable and fixed effects, and homoscedasticity of residual variance. For more amenable computation, we ran this model 1000 times using bootstrap resampled data. We performed bootstrap resampling at the observation level, sampling CD4 measurements with replacement from the prefiltered dataset of all CD4 observations across individuals, giving equal probability to every observation. This approach preserved the hierarchical structure of observations nested within individuals and phylotypes. Individuals reduced to a single observation in a bootstrap sample (e.g. ~10% for the subtype B analysis) were kept; while slope estimation is limited in such cases, the mixed-effects model accommodates varying numbers of observations per individual. The very few phylotypes with no measurements in some bootstrap iterations (e.g. ~5% for subtype B and a minimum clade size of 30) were assigned missing values for those iterations. Standard errors for phylotype-specific slope estimates and variance components were calculated from the empirical distribution of bootstrap estimates, excluding missing values. We computed *t*-statistic values and *P*-values for the phylotype slope effect size based on a standard two-tailed test. *P*-values ≤ .05 were considered statistically significant. Although this model does not incorporate a sparsity-inducing prior or penalty on phylotype-level effects, at this stage of the analysis, we can accept a higher false-positive rate to reduce the risk of missing biologically important effects.

We also conducted a sensitivity analysis that included two alternative CD4 decline models to ensure that the proposed ML method was appropriate for shortlisting suspected VOIs. The first implementation was a Bayesian version of this random effect model, specifying weakly informative normal priors on phylotype effects (see Text S2). While this model better controls for multiple testing, it strongly shrinks estimates towards zero and penalizes small phylotypes with large effects. Therefore, no phylotype slopes reached a Bayesian *P*-value < .05 in this case. A more appropriate significance level for this conservative model was defined as > 80% posterior probability of having a CD4 slope more negative than the backbone (PP) calculated as the proportion of posterior draws that are negative. For a comprehensive sensitivity analysis, we also designed another model to account for some of the limitations of the ML and Bayesian hierarchical CD4 decline models, which were both premised on normally distributed phylotype-level effects on CD4 slope. We hypothesized that a normally distributed phylotype effect may be inappropriate in the presence of the punctuated evolution of a new variant with a large difference in virulence. We used the R2-D2 prior (Zhang *et al*. 2020), a hierarchical non-normal prior that improves estimation under sparsity by allowing large effects on relatively small groups, a scenario that is biologically plausible in this study. It consists of a regularized shrinkage prior for regression coefficients based on specifying a prior belief about the marginal *R*^2^ of the model (i.e. the proportion of variance explained by fixed effects), rather than directly setting priors on individual coefficients. This R2-D2 model we implemented also modelled all subtype-specific data jointly but using fixed instead of random effects on phylotype slopes and intercepts (see Text S3).

After the sensitivity analysis confirmed the reliability of the ML model with a random effect on phylotype, we performed a more biologically relevant phylotype-specific comparison of the suspected VOIs against the likely ancestral type. This was accomplished by using a Bayesian mixed-effects model with a fixed effect on phylotype to estimate the overall effect size (i.e. rate of CD4 decline per year and baseline CD4 count) of these phylotypes against the backbone phylotype. Instead of running the model considering only each suspected VOI and the backbone, we firstly also included non-VOIs (i.e. all other phylotypes combined) and found that the difference between the rate of CD4 decline of backbone and non-VOIs was negligible, similar to the Bayesian joint viral load model (Fig. 2A) and reinforcing its suitability as the reference group in evolutionary and clinical terms. We therefore excluded non-VOIs and focused on measuring differences between individual VOIs and the backbone. We also implemented this model in brms v2.19.0 (Bürkner 2017), with two independent chains of 5000 MCMC iterations, discarding 40% as burn-in, and assessing convergence using R-hat < 1.01 and ESS > 400. We applied conservative and weakly informative normal priors to the phylotype slope (mean = 0 and SD = 20 cells/mm^3^/year) and phylotype intercept (mean = 0 and SD = 20 cells/mm^3^) coefficients.

### Targeted analysis on variant of interest phylotypes

To identify phylotypes with potential enhanced virulence, we combined estimates and *P*-values for the two viral load analyses (Tables S6 and S7) and the two CD4 decline analyses (Tables S8 and S11) and computed their intersection and extreme values (Table 2). We designated VOIs based on intersecting higher viral load and faster CD4 decline, statistically significant differences, or more extreme values in either viral load or CD4 analyses.

**Table 2:** Estimates from the two viral load and two CD4 decline models combined. The VOIs (PT.B.40.UK, PT.B.69.UK, and PT.B.133.UK) are bolded and underlined. The table is ordered by the numeric ID of phylotypes inside the subtypes B, C, A1, and CRF02_AG, respectively. Only phylotypes significant in at least one of the four statistical analyses are presented. The sample sizes represent unique individuals and are smaller than the minimum clade size = 30 in some cases because phylotypes considered all available pretreatment sequences regardless of matching with viral loads and CD4 count data.

		**Viral load models**					**CD4 count decline models**				
**Subtype**	**Phylotype ID**	***T*-test FDR adjusted**		**Bayesian joint**		**Sample size**	**ML (random effect on phylotype)**		**Bayesian (fixed effect on phylotype)**		**Sample size**
		**Estimate**	***P*-value**	**Estimate**	***P*-value**		**Estimate**	***P*-value**	**Estimate**	***P*-value**	
B	3	4.375	1	4.517	.3	19	−2.055	.51	–	–	22
B	4	5.112	.048	4.804	.18	13	−1.695	-	–	–	6
B	7	–^a^	–	4.744	.42	5	−0.847	.87	–	–	7
B	8	4.358	1	4.403	0	119	7.66	.061	–	–	115
B	14	4.543	.89	4.577	.58	41	6.27	.13	–	–	35
B	18	4.855	.19	4.76	.22	32	1.323	.58	–	–	24
B	20	4.883	.0092	4.819	.018	69	−3.415	.55	–	–	63
B	24	4.82	.11	4.735	.35	28	−7.638	.033	−19.249	.073	30
**B**	**40**	**5.11**	**.0023**	**4.927**	**.004**	**38**	**−6.159**	**.1**	**−13.00** ^b^	**.19**	**32**
B	62	4.783	.19	4.719	.41	33	−5.907	.05	−17.44	.09	32
**B**	**69**	**5.076**	**.038**	**4.866**	**.033**	**26**	**−7.978**	**.0024**	**−22.32**	**.047**	**23**
B	77	4.693	.66	4.65	.88	12	−3.687	.034	−12.706	.38	16
B	79	5.102	.034	4.84	.088	18	−0.185	.92	–	–	13
B	84	4.821	.19	4.708	.5	17	−8.173	.03	−23.758	.062	14
B	90	4.627	.74	4.627	.99	19	−7.987	.0033	−25.673	.012	23
B	101	5.237	.056	4.914	.019	20	1.432	.4	–	–	18
B	118	4.584	.87	4.607	.85	23	−8.773	.0039	−21.484	.027	24
B	125	5.287	.038	4.843	.11	11	−4.051	.36	–	–	11
**B**	**133**	**4.535**	**.9**	**4.588**	**.72**	**17**	**−10.182**	**.00004**	**−31.861**	**.0067**	**19**
B	137	4.955	.038	4.777	.22	19	−8.61	.02	−20.907	.057	18
C	4	4.162	1	4.437	.4	21	0.163	.96	–	–	27
C	14	4.981	.046	4.651	.19	26	−0.168	.89	–	–	16
C	18	5.076	.046	4.606	.39	12	−0.037	.98	–	–	9
C	20	–	–	4.51	.9	6	−0.018	.98	–	–	5
A_A1	3	4.369	.72	4.404	.86	77	−15.564	.019	−18.771	.012	57
A_A1	7	–	–	4.423	.81	8	1.627	.62	–	–	9
A_A1	8	4.373	.72	4.408	.92	40	−7.336	.019	−22.834	.012	34
CRF_02_AG	3	4.316	.91	4.54	.56	17	−2.93	.76	–	–	16
CRF_02_AG	7	4.305	.91	4.55	.64	12	−9.919	.006	−16.22	.28	10
CRF_02_AG	8	4.36	.91	4.544	.59	20	2.605	.56	–	–	14

^a^En dash symbols (–) represent ‘not applicable’ and were added for absent estimates due to, e.g. small sample sizes for the viral load models or not being a suspected VOI based on CD4 decline for the Bayesian fixed effect model against backbone. ^b^PT.B.40.UK is not considered a VOI based on CD4 decline, but its CD4 decline estimate was included due to its VOI status (based on viral load).

To further investigate if demographic variables in VOIs were significantly different from those in the backbone, and to identify potential confounders given the substantial association between viral and host factors, we performed several statistical tests. For a second time, we used a Welch’s *t*-test to investigate the mean age at diagnosis (see Text S4 for age group categories) of each of the three individual VOIs against the backbone. In addition, we used chi-square tests to compare the observed versus expected frequencies across categories of ethnicity, risk group, and region of diagnosis (see Text S4 for categories of each variable). This enabled the analysis of deviations from expected distributions under the null hypothesis of independence (i.e. no association between the row and column of the contingency table).

We also employed the previously described method of estimating internode lengths from a time-scaled phylogenetic tree, which can be used as proxies of maximum transmission intervals (Lewis *et al*. 2008, Hughes *et al*. 2009), to provide a simple comparison between transmission rates of VOIs and non-VOIs. Internode lengths were calculated as the time differences between successive internal nodes, excluding edges that connect nodes to terminal tips.

To identify non-UK sequences closely related to VOIs, we downloaded 167 710 HIV-1 subtype B global sequences (pol CDS, minimum length = 950) from the Los Alamos National Laboratory (LANL) HIV Sequence Database (https://www.hiv.lanl.gov/) as of 26 June 2023 and performed BLAST v.2.9.0 (Altschul *et al*. 1990) similarity searches interfaced using rBLAST v0.99.2 (Hahsler and Nagar 2024) to obtain 90%, 95%, 96%, 97%, 98%, 99%, and 100% percent identity matches (Table S18). We used a random sample of 250 matched global sequences alongside the three VOIs to build ML phylogenies and characterise their global spread. To confidently estimate TMRCA of these VOIs, we compared results from ≥1000 trees resulting from a fast ML parametric bootstrap method implemented in treedater v0.5.3 (Volz and Frost 2017) with an uncorrelated additive clock model (Didelot *et al*. 2021) and a Bayesian MCMC approach from BEAST v1.10.4 (Suchard *et al*. 2018) with an uncorrelated lognormal relaxed clock (Drummond *et al*. 2006).

### Consensus phylotype and whole-genome sequence analyses

**Figure 1:**
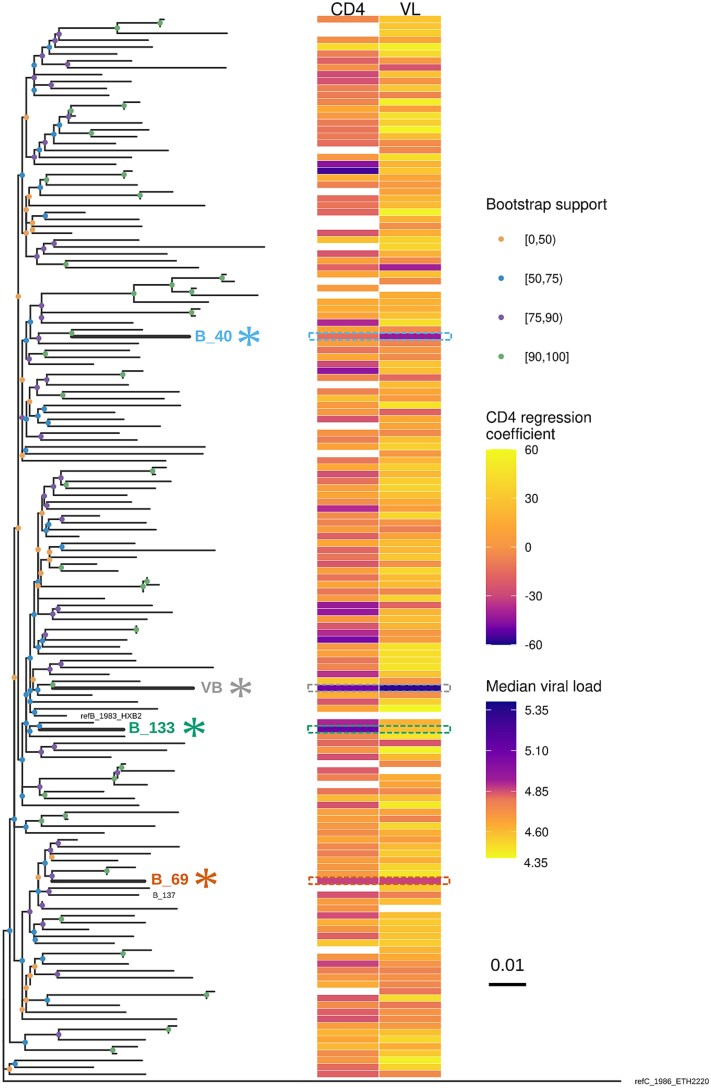
Maximum likelihood (ML) tree of partial-pol consensus subtype B phylotypes, VB clade consensus (sequences from Netherlands only), and subtype C reference. Node colours represent bootstrap support intervals. CD4 regression coefficients (in cells/mm^3^/year) from a fixed-effects ML CD4 decline model against the backbone phylotype and median viral loads (in log_10_ copies/ml) given by the Bayesian joint model are represented by colour bars. VB clade CD4 and VL estimates were extracted from the article describing its discovery (Wymant *et al*. 2022). More negative CD4 estimates and more positive viral loads (as shown by the reversed colour gradient) represent increased virulence. Gradient colour transitions were optimised to enhance the visual distinction of potentially virulent phylotypes. VOIs are indicated by asterisks in the phylogenetic tree and labels of other phylotypes were removed for clarity. CD4 coefficients with associated standard errors > 30 cells/mm^3^/year are omitted from the colour bars.

We obtained consensus phylotype sequences for the four analysed subtypes using seqinr v4.2.8 (Charif and Lobry 2007) with the site majority method (i.e. the high-frequency character is returned as consensus) and a minimum relative frequency threshold of 80% (Figs 1 and S7).

We analysed whole genome sequence (WGS) data to identify any common polymorphisms among VOIs that may provide a mechanism for altering virulence. VOIs thst were defined based on a large set of partial-pol sequences were analysed in combination with a smaller set of UK HIV-1 WGS data from the INITiO study (Mbisa *et al*. 2020) on recently infected individuals from the UK between 2015 and 2021 (*n* = 1581) and COMPARE-HIV (Fisher *et al*. 2010) study, which generated WGS from a previous Brighton HIV cohort study between 2000 and 2006 (*n* = 287). Therefore, to extrapolate whole genomes associated with each subtype B VOIs, we performed a comparative genomic analysis of all sequences within each VOI against these two WGS cohorts (Text **S5**). We then tabulated these genome-wide mutations from the WGS, forming a monophyletic clade with the identified VOIs at the partial-pol ML phylogenetic trees (Text S5, Fig. S5, Data S1). We also investigated the proportion of viruses capable of using the CXCR4 coreceptor associated with faster disease progression by analysing V3 loop sequences (Text S6).

## Results

### Correlation of genomic and clinical HIV-1 data

The number of samples satisfying inclusion criteria used for specific analyses is cross-tabulated with HIV-1 subtype and PLWH characteristics in Tables 1, S1, and  S2. Despite a few slight deviations when compared to the distribution of demographic variables of the database, the included partial-pol sequence and clinical data closely reflect the distribution of sex assigned at birth, ethnicity, risk group, region of diagnosis, and age groups of the UK HIV-1 epidemic (Text S7, Tables S1 and S2). From the total amount of 98 803 UKRDB PLWH and 145 094 sequences (75.7% with one sequence, 14.6% with two sequences, and 9.7% with >2 sequences), 40 888 PLWH and their respective first high-quality partial-pol sequences while treatment naive were retained after multiple filters (see Methods: Study Population and Sequence Selection, Table 1). Additionally, a total of 41 185 viral load measurements (median: 2 per person, IQR: 1–5) were successfully cross-referenced with sequences and included in the statistical analysis. A total of 77 541 CD4 measurements were available across all individuals with included sequences (median: 1 per person, IQR: 1–3). After restricting to individuals with more than one measurement and applying the filtering criteria, 42 355 measurements remained for analysis.

### A small fraction of subtype B phylotypes have higher viral loads and faster CD4 declines

After standard genomic alignment and phylogeny reconstruction, we tested four tree partitioning methods. When fitting the Bayesian joint viral load model using partitions designated by fastbaps and treecluster, we found that the clusters which overlapped with subsequently identified VOIs from treestructure also exhibited statistically significant higher viral loads. These clusters, however, exhibited a larger and more diverse number of sequences, as well as lower viral loads when compared to treestructure partitions (Fig. S3). Most importantly, the treestructure method (Volz *et al*. 2020) with a small threshold of minimum size (i.e. 30) explained a larger proportion of the variation in viral loads when compared with partitions of similar complexity estimated using genetic distance (treecluster) and hierarchical clustering (fastbaps) methods (see Methods: Phylogenomic Analyses, Figs S2–S4 and Table S4). Therefore, our main focus is to present results from this analysis for the four analysed subtypes.

We employed two different methods to investigate subtype-specific differences in pretreatment individual mean viral loads across phylotypes: (i) a Welch’s *t*-test that compares each phylotype against the large ancestral type from which all the other phylotypes are derived (i.e. backbone) and corrects for multiple testing and (ii) a Bayesian joint model that uses data from all phylotypes (see Methods and Fig. S1). Both of these analyses identified similar phylotypes as possessing higher viral loads, with the *t*-test adjusted using the FDR method detecting *n* = 9 (*n* = 7 subtype B and *n* = 2 subtype C) (Table S6) and the Bayesian joint model highlighting *n* = 4 phylotypes (all subtype B) as significant (Table S7). Most importantly, three of those had significantly higher viral loads in both analyses (PT.B.20.UK, PT.B.40.UK, and PT.B.69.UK).

To investigate phylotype-specific differences in the rate of CD4 decline, we also used two different methods: an ML model with a random effect on phylotype to find outlying phylotypes and a Bayesian model with a fixed effect on phylotype to estimate its overall effect size on suspected VOIs highlighted by the aforementioned random effect model. Both models were adjusted for relevant covariates. From the random effects model, *n* = 12 phylotypes (*n* = 9 subtype B, *n* = 2 A1, and *n* = 1 CRF_02_AG) had significantly faster decline (Table S8) and we classified as ‘suspected VOIs’. When comparing these suspected VOIs against the backbone using the Bayesian fixed effects model, six remained statistically significant.

Results from the sensitivity analysis of the CD4 decline models showed consistent overall effect size estimates for the rate of CD4 decline across models. However, the more conservative Bayesian random effect model with a normal prior on phylotype coefficients would not shortlist any phylotypes based on Bayesian *P*-values (*P* < .05) for CD4 slopes. When using a more appropriate Bayesian threshold for this model, the model identified five phylotypes (four overlapping with those from the ML method) at PP > 90%, and 20 phylotypes (10 overlapping with ML) at PP > 80% (Table S9, Fig. S8B). The second model, which uses the R2-D2 prior on fixed effects to mitigate over-shrinkage in smaller groups, yielded more negative phylotype slope estimates than the two random effect models (Table S10, Fig. S8B). This model shortlisted two phylotypes using *P* < .05 (PT.A1.3.UK and PT.B.133.UK), 12 phylotypes (six overlapping with ML) at PP > 90%, and 30 phylotypes (10 overlapping with ML) at PP > 80%. Most importantly, 10 phylotypes were consistently identified across both analyses (i.e. *P*-value < 0.05 in the ML CD4 model and a PP > 80% in the two Bayesian sensitivity analyses). Therefore, the observed agreement between the ML CD4 model and the two alternative modelling approaches supports its use as a rapid and reliable method for detecting phylotypes with outlying slopes. Notably, when comparing estimates across the four CD4 decline models, the central estimates from the ML and Bayesian random effects models are generally consistent. However, the Bayesian random effects model exhibits noticeable shrinkage towards no effect as hypothesized (Fig. S8B).

The joint examination of viral loads and CD4 results (Tables 2, S3, S6–S8, and  S11) revealed that one subtype B phylotype (PT.B.69.UK) showed significant differences in all four analyses. However, two other phylotypes that were not detected in all analyses had, respectively, the highest viral load (PT.B.40.UK) and the fastest CD4 decline (PT.B.133.UK) alongside stronger statistical significance. Based on this, we classified PT.B.40.UK, PT.B.69.UK, and PT.B.133.UK as VOIs to allow more targeted investigations.

The maximum likelihood phylogeny reconstructed from the analysed subtype B partial-pol sequences is shown in Fig. S6. The phylogeny of consensus phylotype sequences (Fig. 1) and its distance matrix (Fig. S7) highlight the support for their assignments and demonstrate that they show substantial divergence from the recently discovered VB clade from the Netherlands (Wymant *et al*. 2022). The proportion of VOI sequences across all analysed subtype B samples analysed in this study was similar in more recent years (i.e. after 2015), accounting for 1.1% (from 2999 total sequences) when compared to before 2015 (0.8% from 20 776 sequences) (Table S12). Therefore, despite estimated increased virulence, this analysis suggests that VOIs did not increase in frequency rapidly.

### Viral load and CD4 differences in subtype B variants of interest

**Figure 2:**
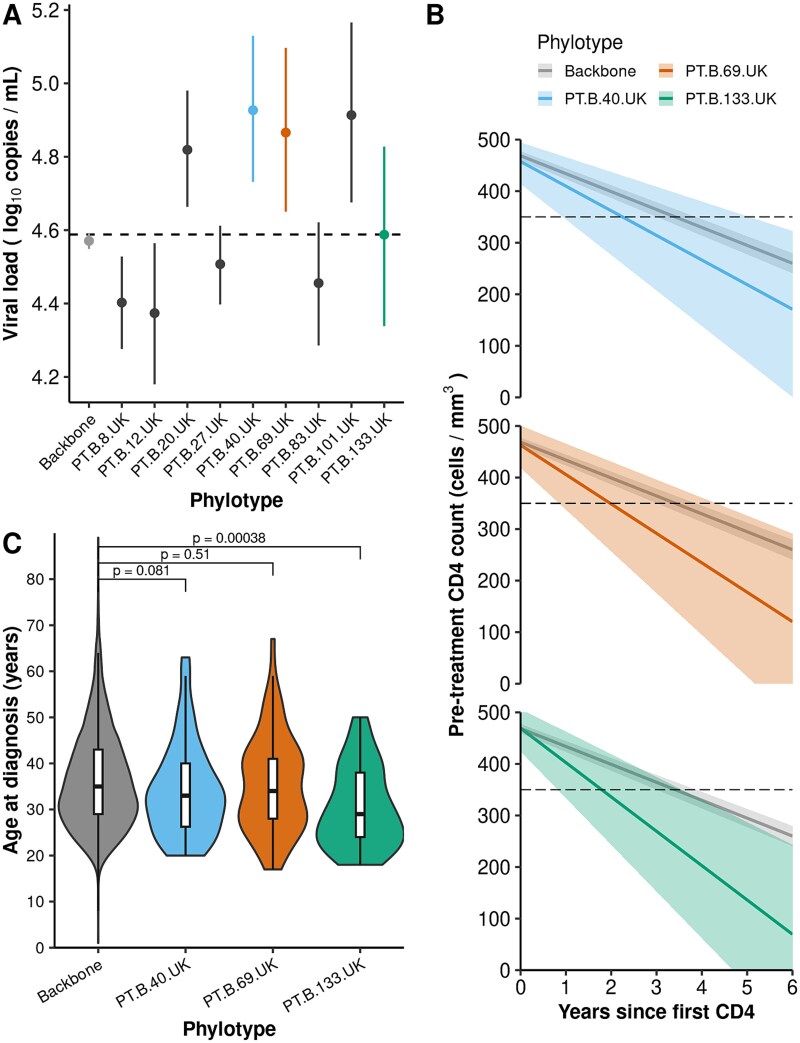
Clinical and demographic characteristics of the HIV-1 subtype B VOIs. (A) Bayesian estimates of a joint model of pretreatment viral load (in log_10_ copies/ml) differences and 95% CIs. Phylotypes with significantly elevated or reduced viral loads are presented alongside the backbone and the PT.B.133.VOI phylotype. The estimated mean viral load across all subtype B phylotypes is represented by the dashed horizontal line. (B) Expected decline in CD4 (measured in cells per mm^3^ of blood with respective 95% CIs) in the absence of treatment as measured by the fixed-effects Bayesian model fitted to each VOI against the reference group (MSM in their thirties and within the backbone phylotype. PT.B.40.UK is presented for comparison despite only displaying elevated viral loads. (C) Violin and boxplot of age at diagnosis for the VOIs compared with the backbone phylotype violin plots display the age distribution density for each phylotype, with overlaid boxplots showing the median (central line), IQR (box limits represent 25th and 75th percentiles), and whiskers extending to values within 1.5 × IQR. *T*-test *P*-values adjusted for multiple testing using the FDR method are shown.

To evaluate the impact of phylotype membership on HIV-1 prognostic markers, we conducted comprehensive statistical analyses of viral loads and CD4 declines. Figure 2 shows the phylotype-specific viral load estimates given by the Bayesian joint model, CD4 cell declines from the Bayesian fixed effects model, and age at diagnosis for VOI phylotypes. When compared to the population mean and the backbone phylotype (both of which have very similar estimates around 4.58 log_10_ copies/ml), PT.B.40.UK and PT.B.69.UK VOIs clearly demonstrate higher viral loads, 4.93 log_10_ copies/ml (95% CI: 4.73–5.13) and 4.87 (95% CI: 4.65–5.10), respectively. Two other phylotypes also presented elevated viral loads (PT.B.20.UK and PT.B.101.UK) for this model, despite not meeting the VOI criteria, while four others presented lower values, and the PT.B.133.UK VOI presented usual values of viral load (Fig. 2A, Tables 2 and S7).

To provide a detailed perspective on the accelerated disease progression of these VOIs, we extracted phylotype-specific slope and intercept regression coefficients from the Bayesian fixed effects CD4 model. Until 2015, ART guidelines advised starting treatment when the CD4 count fell below 350 cells/mm^3^, whereas current recommendations suggest initiating treatment as soon as diagnosed and regardless of CD4 count (British HIV Association 2015). While individuals in the backbone phylotype take 3.5 years (95% CI: 3.1–3.9) to reach this 350 cells/mm^3^ threshold, the CD4 cell decline of VOIs is up to twice as fast. PT.B.40.UK reaches this threshold in 2.3 years (95% CI: 1.0–5.1 years), PT.B.69.UK in 2.0 years (95% CI: 10.8 months to 4.4 years), and PT.B.133.UK in 1.8 years (95% CI: 10.8 months to 3.6 years). In other words, relative to the reference group (MSM in their thirties infected with the backbone phylotype), PT.B.133.UK has an additional CD4 decline of 32 cells/mm^3^/year (95% CI: 9–54), PT.B.69.UK has an additional depletion of 22 cells/mm^3^/year (95% CI: 0–44), and PT.B.40.UK has an additional decline of 13 cells/mm^3^/year (95% CI: increase of 6 to decrease of 32) (Figs 2B, S8, and  S9; Table S11). Although the rate of CD4 decline is faster in PT.B.133.UK, the median baseline CD4 counts of the other two VOIs are slightly lower. However, it is important to note that there is high uncertainty in the phylotype-specific CD4 baseline levels (Table S13), leading us to present an alternative scenario where the backbone and VOIs start at a common baseline CD4 of 469 cells/mm^3^ as estimated for the reference group. The results remained similar under this assumption (Fig. S10).

We also evaluated the rate of CD4 decline across several sociodemographic groups. Older people (>60 years) have a baseline CD4 count of 86 cells/mm^3^ (95% CI: 34–136) lower than the reference group. This baseline level is also reduced for individuals in their fifties (54 cells/mm^3^; 95% CI: 28–79), females (32 cells/mm^3^, 95% CI: 7–58) and individuals in their forties (34 cells/mm^3^, 95% CI: 19–49). The effects of these variable categories on the slope (rate of CD4 decline per year) are not as pronounced as the phylotype effects (Table S13). These variables were included in all multivariate modelling to adjust for possible confounding given the strong associations between epidemiological, demographic, and viral factors.

The mean age at diagnosis for individuals infected by the PT.B.133.UK VOI was significantly lower (mean 30.33 years; SD: 8.63, FDR-adjusted *P*-value = .0003) when compared to individuals within the backbone phylotype (mean: 36.24 years, SD: 10.40) (Fig. 2C, Table S14). Ethnicity across VOIs was consistent with the distribution of subtype B infections in the UK (Table S1), i.e. ~70% of people being of White ethnicity (Fig. S11) with a few exceptions (Text S7, Fig. S11). Observed individuals in London were fewer than expected for VOIs PT.B.40.UK and PT.B.69.UK (Fig. S12B and C). The count of people in the North of England was higher than expected for PT.B.40.UK (Fig. S12B). The same occurred in the South of England for PT.B.69.UK (Fig. S12C). The appearance of PT.B.133.UK is also slightly higher than expected in the Midlands and East of England (Fig. S12D). However, spatial structure is unsurprisingly common across phylotypes (Fig. S12A). The risk group and sex at birth distribution is in line with the database, with most phylotypes composed by >75% homo/bisexual and male individuals, with only a few deviations (Text S8, Figs S13 and S14). Median transmission intervals were similar between subtype B VOIs (8.28 months; IQR: 0.002–26.7) and non-VOIs (6.9 months; 0.002–23.8). The mean transmission intervals, respectively, 18.7 (SD: 26.6) and 17.8 (SD: 29.0), more closely approximated the previous median estimate of 14 months for MSM in the UK (Hughes *et al*. 2009).

### Growth rates and drug resistance patterns of HIV-1 subtype B variants of interest

**Figure 3:**
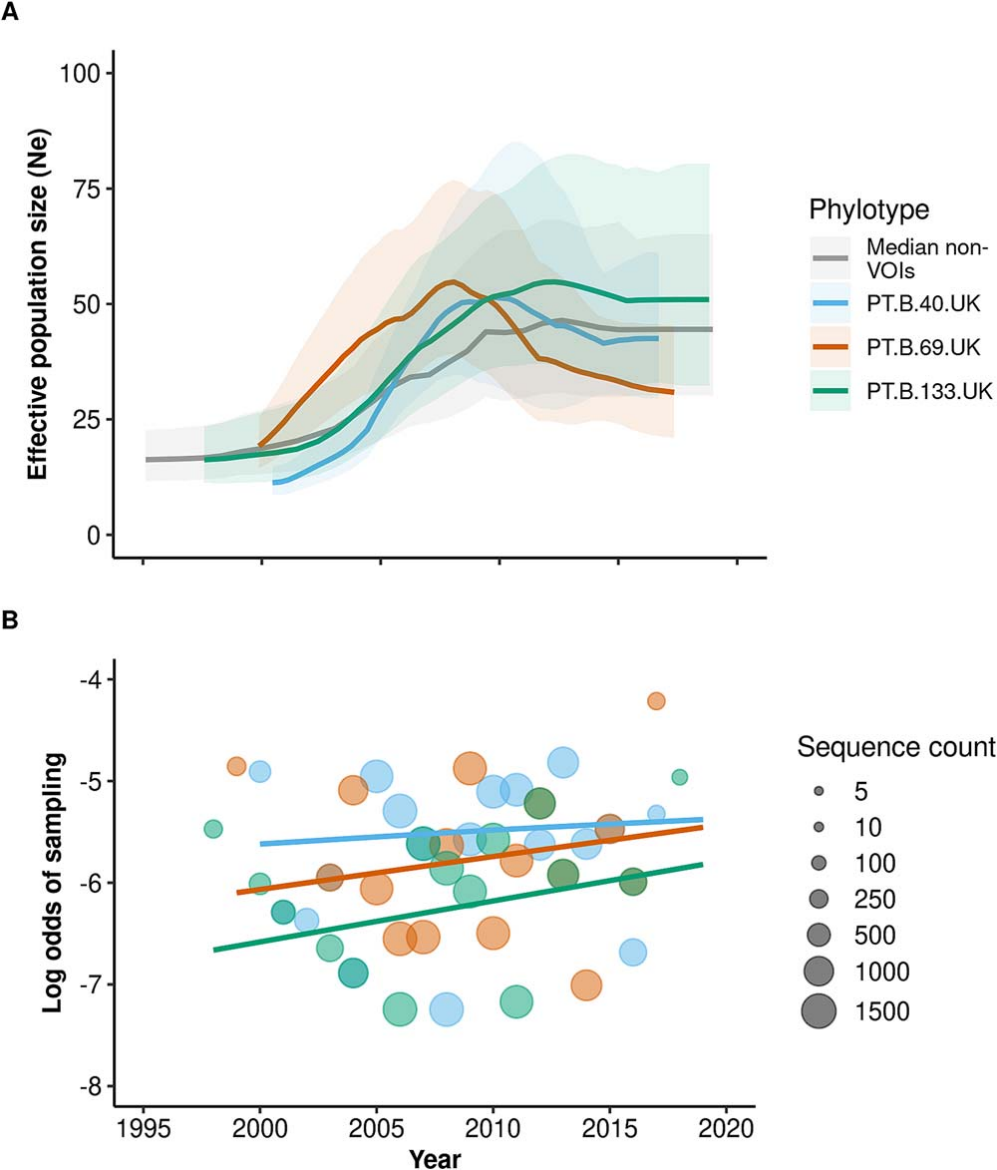
Growth rates of the three identified VOIs. (A) Ne over time (in units of years, as scaled by the coalescent generation time) for individual HIV-1 subtype B VOIs calculated using 50 grid points, precision (smoothness) of 50, and the skygrid demographic model. Median Ne estimates for the other 149 non-VOI phylotypes were calculated using linear interpolation on a common time axis. Shaded regions indicate 95% confidence intervals (CIs) estimated using parametric bootstrap. (B) Change in log odds frequency over time among VOIs. VOI phylotypes follow the same colour scheme as (A). Sequence count refers to the total number of sequences (sample size) at each timepoint, including the VOI phylotype under consideration and all the other background sequences.

Phylodynamic estimates of effective population size over time (Ne_(t)_) indicated that VOIs have similar growth patterns to non-VOIs (Fig. 3A). Ne(t) of most VOIs expanded between the early 2000s and 2010, although the magnitude and smoothness of the peaks are dependent on how the phylodynamic model is calibrated (see Methods, Fig. S15). The logistic growth model for estimating the probability of sampling each VOI also indicates that even if a selective advantage exists—which remains uncertain—its magnitude is expected to be very small (Fig. 3B).

We did not identify any concerning patterns of drug resistance mutations (DRMs) associated with VOIs, and resistance score profiles were similar to non-VOIs, although PT.B.40.UK had the PR:L90M DRM in high frequency and the RT:K103N DRM was found in a low percentage of PT.B.69.UK sequences (Text S9, Tables S15 and S16). Furthermore, due to reduced sample sizes (i.e. lack of sufficient follow-up of treated PLWH), it was not possible to confidently investigate the hypothesis that these subtype B VOIs are disproportionately contributing to the increase of treatment failures (Text S10, Fig. S16, and Table S17).

### HIV-1 subtype B variants of interest present differences in global and regional distributions

By performing BLAST similarity searches against the >167 000 HIV-1 subtype B sequences from the LANL database, we identified a few sequences PT.B.40.UK (*n* = 8) outside the UK having identity ≥98% (i.e. dissimilarity of 0–20 mutations) along the partial-pol gene. No global (non-UK) matches at this identity threshold were found for PT.B.69.UK and PT.B.133.UK (Table S18). For the two VOIs with the fastest decline in CD4 and consequently most likely accelerated disease progression (PT.B.69.UK and PT.B.133.UK), the closest hits were detected at 97% identity (*n* = 11 and *n* = 149 matches, respectively). To understand the global routes for dispersal and potential sites of emergence of these five VOIs, we combined the UK pol sequences from this study assigned to each VOI with a random sample of 250 unique sequences from 95% identity BLAST hits. ML and Bayesian dating analysis of VOIs alongside these closest global matches were highly compatible, suggesting that PT.B.UK.69 emerged (or was introduced in the UK) approximately in May 1986 (95% CI: December 1981 to October 1990) for the Bayesian estimate and May 1985 (95% CI: September 1977 to June 1992) for the ML model. Comparably, TMRCA of PT.B.133.UK was predicted around August 1987 (95% CI: August 1980 to March 1994) and May 1990 (95% CI: June 1983 to December 1995), respectively. The estimated emergence of PT.B.40.UK was more recent: April 1993 (95% CI: January 1989 to January 1996) or June 1993 (95% CI: April 1988 to July 1997) (Fig. S17).

**Figure 4:**
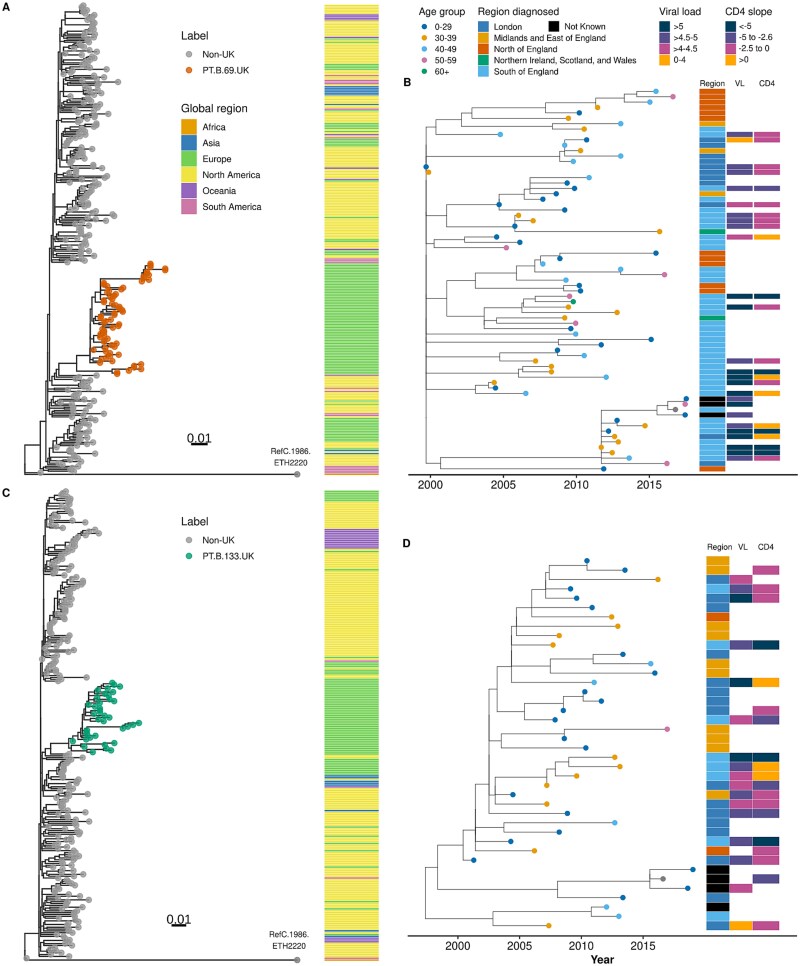
Evolutionary history and global context of two VOI phylotypes (PT.B.69.UK and PT.B.133.UK). (A, C) Maximum likelihood tree of the partial-pol gene of PT.B.69.UK and PT.B.133.UK combined with 250 random non-UK BLAST global matches at an identity threshold of 95%. Side panel annotation depicts global regions of sampling. (B, D) Time-scaled phylogenies of the partial-pol gene of PT.B.69.UK and PT.B.133.UK, respectively. Tip points are coloured by age group at diagnosis, and side panels represent the region of diagnosis, mean viral loads (in log_10_ copies/ml), and CD4 slope ranges (in cells/mm^3^/year) per individual. Absent entries in side panels occur because some individuals do not have pretreatment viral loads or at least two CD4 measurements before treatment or these did not pass filtering steps.

The worldwide distribution over time (Fig. S18) and the ML phylogeny of PT.B.40 (Fig. S20), PT.B.69 and PT.B.133 (Fig. 4A and C) alongside global closest sequences suggest that progenitors of these VOIs may have been circulating and diversifying, possibly in North America in the late 1970s and early 1980s, before giving rise to these VOIs. The VOI phylotypes appear to be very rarely transmitted to other continents and their progenitors hardly form substantially big clusters (*n* > 10) outside of Europe and North America, except for one of PT.B.40 in Asia (Fig. S20) and another of PT.B.133 in Oceania (Fig. 4B). No clades other than PT.B.40.UK, PT.B.69.UK, and PT.B.133.UK were found to descend from long branches in these global phylogenies, although both of the last two observations are probably highly influenced by unsampled diversity in some countries and overrepresentation in others. Time-scaled trees of PT.B.40.UK (Fig. S21), PT.B.69.UK, and PT.B.133.UK (Figs 4B and D and S19**)** with age, region, viral load, and CD4 annotations highlight a substantial mixing across different age groups, the existence of region-specific subclades, and patterns consistent with some degree of heritability of viral load and CD4 declines for closely related tips. Using a POUMM model for univariate continuous traits fitted to an ML tree composed of all subtype B sequences with viral loads (*n* = 8789), we estimated a phylogenetic heritability of 18% (95% highest posterior density [HPD] interval: 12%–23%), consistent with previous estimates (Mitov and Stadler 2018).

### Potential whole-genome signatures of enhanced virulence

Common mutations across three subtype B VOIs (here considering PT.B.40.UK, PT.B.133.UK, and PT.B.137.UK, see details in Text S5) and VOI-specific mutations are tabulated in Data S1 (see also Fig. S5 and Text S5). We observed 75 changes relative to HXB2 at the nucleotide level that were defining (present in >80% of sequences from each VOI) and shared among these three VOIs with closely related WGS matches, including 44 nonsynonymous substitutions, one deletion (of one nucleotide), and 30 synonymous substitutions. Of the nonsynonymous changes, none are considered to be a DRM, one is a CTL escape mutation (pol integrase:E11D) with a typical value for escape (i.e*.* strength of selection) when compared to other integrase mutations (log odds = 10.973, specifically in individuals with the Human Leukocyte Antigen [HLA] allele B*44) (Carlson *et al*. 2012), and one mutation (pol integrase:V201I) is in common with the VB variant (Wymant *et al*. 2022). These two mutations are not rare in subtype B sequences, occurring in 34%–47% of treatment-naive and 24%–43% of treated PLHW (https://hivdb.stanford.edu/hivseq/by-patterns/report/?mutations=IN%3AE11D%2CIN%3AV201I&name=IN%3AE11D%2BIN%3AV201I). We found that most of the 44 nonsynonymous substitutions shared across VOIs are common or fixed in global publicly available subtype B sequences. Most importantly, only two of these shared nonsynonymous mutations are not present in the consensus sequence of the analysed WGS cohorts (see Data S1; Tab 1). These are the previously mentioned CTL escape mutation (pol integrase:E11D) and env:gp120:Q8K.

Regarding shared VOI mutations relative to HXB2, we identified four mutations located in close proximity inside the hypervariable V3 loop of the env gene (gp120:V318Y, I320T, K322E, and N325D) that rarely occurred worldwide (Data S1, Text S5). Interestingly, gp120:Y318 and D325 are coreceptor binding sites inside the V3 loop and gp120:K322 is a coreceptor-specific (R5/X4) site inside the V3 loop (Korber and Gnanakaran 2009). It is unlikely that these few mutations represent a novel mutation signature of virulence given that they are also present in the consensus of the two WGS cohorts analysed.

Lastly, we investigated whether subtype B VOIs exhibited a higher proportion of viruses capable of using the CXCR4 coreceptor, which is associated with faster disease progression (Connor *et al*. 1997) However, no statistically significant difference was found when compared to the subtype B backbone (Wilcoxon rank sum test *P*-value = .77 and Beta regression *P*-value = .91) (Text S6).

## Discussion

Viral loads and the rate of CD4^+^ T-cell decline are used as proxies for HIV-1 virulence due to their significant correlation with disease progression in the absence of ART (Fraser *et al*. 2007, Herbeck *et al*. 2014, Pantazis *et al*. 2014, Wymant *et al*. 2022) In this study, we selected a coalescent-based phylodynamic approach to partition HIV-1 large trees into phylotypes with potentially distinct transmission patterns. Phylotypes derived by this approach explained more variance in viral loads in comparison with partitions of similar complexity derived from two other partitioning methods based on genetic distance thresholds and hierarchical clustering. Among such phylotypes, we identified VOIs based on their virulence estimated using four statistical models—two on viral loads and two on CD4 cell declines. The only three VOIs identified were from subtype B, the most widespread in the UK. These VOIs and their progenitors seem to have successfully circulated in the UK and worldwide for the past 40 years. Estimates of effective population size_)_ and growth rates of these variants over time align with the UK HIV-1 epidemic curve while also displaying some heterogeneity in size, time of peak, and current growth rates. Phylogenomic and statistical analyses indicated a slightly younger age at diagnosis for PT.B.133.UK and some geographical differences in the distribution of some VOIs in the UK. However, the modest disparities in sociodemographic and behavioural characteristics are consistent with the hypothesis that the elevated virulence of these lineages is at least partly attributable to viral genetics instead of host factors.

There is conflicting evidence regarding the evolution of HIV-1 virulence over time, which is possibly a result of a mixture of systematic sampling biases, different epidemic circumstances across countries, and lack of studies focusing on specific genotypes (Herbeck *et al*. 2012, Pantazis *et al*. 2014). Before our study, only one article investigated the effect of a particular viral variant/genotype (VB clade) (Wymant *et al*. 2022) on HIV-1 virulence using viral loads and CD4 counts. Therefore, we extend on the usual population-wide studies estimating viral loads and CD4 counts over time by considering the viral genetics component and identifying lineages of HIV-1 that are influencing these clinical prognostic markers. The virulence-transmission trade-off hypothesis provides a possible explanation for these country-specific declining (Blanquart *et al*. 2016), stable (Müller *et al*. 2006), or increasing (Wertheim *et al*. 2019, Wymant *et al*. 2022) patterns of HIV-1 virulence reported during the last few decades. Since viral load (and most specifically SPVL) influences both transmission and the rate of disease progression, virulence will theoretically evolve to an intermediate level (balancing infectiousness and persistence within hosts), reaching an optimal value between 4.52 and 4.75 log_10_ copies/ml in the absence of ART (Fraser *et al*. 2007, Herbeck *et al*. 2014, 2016, Zhao *et al*. 2022). Additionally, results from European seroconverter cohorts revealed that SPVL increased from 4.05 log_10_ copies/ml in 1980 to 4.50 log_10_ copies/ml in 2002 with a tendency of decline thereafter (Pantazis *et al*. 2014). Despite using viral loads measured shortly after diagnosis instead of SPVLs, our Bayesian joint model results align with both studies, as the detected VOI phylotypes fall within the optimal SPVL range (4.65–5.13 log10 copies/ml when considering uncertainty bounds) and the likely ancestral type (backbone) and the mean population value are highly consistent (4.55–4.59). Although the virulence-transmission trade-off hypothesis provides a reasonable explanation for the observed HIV-1 virulence patterns, the number of generations (in terms of serial transmissions) needed to evolve towards this threshold is potentially large.

A meta-analysis estimated differences over time in baseline CD4^+^ T-cell counts and SPVL from a total of 12 studies conducted across HIV cohorts from Europe (*n* = 10), the USA (*n* = 2), Canada (*n* = 1), and Australia (*n* = 1). The inferred rate of baseline CD4 count decline calculated from MSM (*n* = 11 cohorts), heterosexual (*n* = 10), and injecting drug users (IDU) (*n* = 8) varied between −6.01 and − 4.93 cells/mm^3^/year (Herbeck *et al*. 2012). Additionally, the timeframe to reach the 350 CD4 cells/mm^3^ threshold was estimated to have decreased from a mean of 7 years in 1980 to 3.40 years in 2004 (Pantazis *et al*. 2014). This estimate aligns with the backbone phylotype in this study, which reached this threshold in 3.5 years (95% CI: 3.1–3.9). Ultimately, we observed a smaller viral load increase (up to 0.36 log_10_ copies/ml for PT.B.40.UK) and a less pronounced excess decline of CD4 cells/mm^3^/year (32; 95% CI: 9–54) of UK VOIs from this study compared to the recently discovered VB clade from the Netherlands (0.54 log_10_ copies/ml increase and a depletion of further 49 cells/mm^3^/year [95% CI: 20–79], respectively) (Wymant *et al*. 2022). It is relevant to observe, however, that mean SPVL for non-VB (the ‘control’ group) is 0.22 log_10_ copies/ml higher than the viral loads from the UK backbone phylotype (4.79 versus 4.57) —probably due to the inclusion of samples from well-documented early infections in the former—and CD4 model coefficients of VB and the UK VOIs detected here are only 11–25 cells/mm^3^/year apart.

The estimated dates of emergence of UK VOIs (median estimates ranging between mid-1985 and mid-1993) slightly predate the widespread availability of ART, and there are no VOI-signature DRMs or widespread high-resistance DRMs in VOI phylotypes. Therefore, the evolution of these variants was probably not influenced by selective pressures stemming from contemporary ART regimes. The change in HIV-1 clinical management practice in the UK, i.e. the recommendation for initiation of therapy as soon as diagnosed (irrespective of CD4 count) in 2015 not only represents an important and beneficial step for HIV control but also limits our ability to analyse more recent pretreatment clinical data and understand intrinsic virulence mechanisms of HIV-1 in more detail.

Due to the unavailability of UKRDB whole-genome sequences for analysis, we identified the closest matches in the partial-pol segment from two English cohorts with available data. This allowed us to investigate potential convergent genetic factors contributing to differences in viral loads and CD4 declines across VOIs. Although we identified very few whole-genome hits against the VOIs (i.e. not all mutations detected will be clade-defining and undoubtedly found in the VOIs), we still detected 75 mutations (44 nonsynonymous) in common across three VOIs with matching genomes. Among those, one is a CTL escape, one is shared with the VB variant, and four are env V3 loop mutations (gp120:V318Y, I320T, K322E, and N325D). However, only two of the 44 nonsynonymous mutations (pol integrase:E11D and env gp120:Q8K) are unique to the VOIs, i.e. not presented in the consensus sequence of the WGS cohorts analysed. Additionally, we did not find an enrichment of X4-tropic viruses in the potential V3 loop sequences of subtype B VOIs, despite the well-established association between the switch in coreceptor usage from CCR5 to CXCR4 and faster disease progression (Connor *et al*. 1997).

The lack of a clear common genetic cause associated with the elevated virulence of these VOIs (among them and when compared to VB) suggests that these molecular mechanisms are complex or multifactorial. Additionally, potential residual confounding effects cannot be discounted despite attempts to adjust our analyses for several known covariates that influence disease progression. One possible interpretation of the existence of such confounders is that a severe course of infection is correlated with sexual transmission networks. For example, host genetic factors that affect virulence are correlated among individuals from the same finely defined ethnic groups. If these groups also tend to be connected on a transmission network—such as among sexual partners or people who shared needles—this assortative mixing would induce a correlation between host and viral genetic factors. Another possibility is that a co-epidemic of HIV-1 and another sexually transmitted infection (STI) within a single transmission network could alter the course of infection of affected individuals with a closely related virus. We did not have access to data on coinfection with STIs. However, a systematic review based on 37 studies conducted in many countries (Kalichman *et al*. 2011) estimated an STI prevalence of ~15% among PLWH. These coinfections can affect HIV viral load (Serwadda *et al*. 2003, Palacios *et al*. 2007) and infectivity (Serwadda *et al*. 2003). It is important to note, however, that this systematic review provides a cross-sectional estimate and does not directly reflect the probability of coinfection occurring at some point after HIV acquisition, as STIs may resolve over time.

HIV diagnoses in the UK decreased by ~ 50% in the past 10 years. The epidemic dynamics are also changing; acquisition among MSM have been consistently declining, while the proportion of heterosexual cases has increased. Diagnosis of the latter is typically made later in life, with a longer delay following infection, and is likely the driver of an overall shift to higher age at diagnosis (UK Health Security Agency 2024). Therefore, further studies combining phylodynamic and clinical analyses will be essential to establish the potential role of higher virulence in these groups. This work highlights that clinical and genomic surveillance remains critical to monitor changes in HIV-1 virulence and transmissibility, especially in resource-limited settings where HIV drug resistance is a pressing challenge. It also demonstrates that variants with increased virulence are likely more widespread than previously anticipated and potentially emerging independently in other countries. However, their differences can only be identified through widespread surveillance and individual-level linkage of genomic and longitudinal clinical data.

## Supplementary Material

appendix_production_HIV_phylotypes_veaf048

Data_S1_veaf048

Data_S2_veaf048

## Data Availability

Due to ethical and legal considerations, individual-level HIV genetic, demographic, and clinical data from the UKRDB cannot be publicly shared, as they contain sensitive information about study participants. Access to these data is restricted and may be granted for collaborative research projects following approval of a formal proposal by the UKRDB Steering Committee. Consensus partial-pol sequences representing each UK phylotype are publicly available *via* Zenodo (https://doi.org/10.5281/zenodo.14792962). All code used in the analysis is openly available on GitHub (https://github.com/vinibfranc/HIV_UKRDB_phylotypes).
