# Supplementary material for: Evidence for circulation of high-virulence HIV-1 subtype B variants in the United Kingdom
Source: Virus Evol. 2025 May 20;11(1):veaf048. doi: 10.1093/ve/veaf048 (PMC12257091; doi:10.1093/ve/veaf048)
Supplement: appendix_production_HIV_phylotypes_veaf048 [file appendix_production_hiv_phylotypes_veaf048.docx]

**Appendix for Franceschi et al.**

Evidence for circulation of high-virulence HIV-1 subtype B variants in the United Kingdom

Table of Contents

[Supplementary text 6](#_Toc198199293)

[Text S1. Bayesian joint viral load model variables and prior selection 6](#_Toc198199294)

[Text S2. Bayesian joint CD4 decline model with a random effect on phylotype slopes and intercepts 6](#_Toc198199295)

[Text S3. Bayesian joint CD4 decline model with a fixed effect on phylotype intercepts and slopes using a R2-D2 prior 6](#_Toc198199296)

[Text S4. Stratification of ethnicity, risk group, region of diagnosis, and age 7](#_Toc198199297)

[Text S5. Whole-genome sequence analysis 7](#_Toc198199298)

[Text S6. Coreceptor usage prediction 8](#_Toc198199299)

[Text S7. The UK HIV-1 epidemic through the lenses of UKRDB 8](#_Toc198199300)

[Text S8. Examples of sociodemographic structure across non-VOI phylotypes 9](#_Toc198199301)

[Text S9. DRMs and resistance scores 9](#_Toc198199302)

[Text S10. Treatment failures 9](#_Toc198199303)

[Supplementary figures 10](#_Toc198199304)

[Figure S1. Example of *treestructure* output for subtype B, minimum clade size of 30, and BT = 80.. 10](#_Toc198199305)

[Figure S2. Variation in the number of clusters detected by *treecluster* per genetic distance threshold for the two most common genetic distance methods and considering bootstrap support = 80% for subtype B sequences. 11](#_Toc198199306)

[Figure S3. Comparison of clusters/phylotypes given by different partitioning methods for subtype B UKRDB partial *pol* sequences. The leftmost panel shows the ML tree built from 24,100 sequences 13](#_Toc198199307)

[Figure S4. Comparison of the proportion of variance (R^2^) in viral load explained by different partitioning methods and among choices of minimum clade size of *treestructure* for subtype B sequences.. 14](#_Toc198199308)

[Figure S5. Maximum likelihood phylogenetic trees of partial pol sequences from four UKRDB subtype B VOI phylotypes (PT.B.40.UK, PT.B.69.UK, and PT.B.133.UK) combined with INITiO and COMPARE cohort sequences.. 15](#_Toc198199309)

[Figure S6. Maximum likelihood phylogeny of 2051 subtype B partial pol sequences subsampled from the original tree of 24,101 sequences from which the phylotypes with a minimum size of 30 were computed with *treestructure*. 16](#_Toc198199310)

[Figure S7. Nucleotide-level distance matrix of consensus sequences of the 154 subtype B phylotypes for a 995-length *pol* (complete protease and partial reverse transcriptase) alignment. 17](#_Toc198199311)

[Figure S8. Overview of CD4 measurements and differences between the four CD4 decline models investigated. 18](#_Toc198199312)

[Figure S9. (A-C) Absolute number of CD4 measurements pre-treatment (for individuals with more than one measure) per year coloured by episode per individual within the VOIs (A) PT.B.40.UK, (B) PT.B.69.UK, and (C) PT.B.133.UK. (D) Pre-treatment CD4 declining slopes for each individual infected by the VOIs coloured by age group. The dashed line indicates CD4 = 350 cells/mm^3^. 19](#_Toc198199313)

[Figure S10. Expected decline in CD4 (measured in cells per mm^3^ of blood) in the absence of treatment as measured by the fixed effects mixed Bayesian model against the backbone phylotype.. 20](#_Toc198199314)

[Figure S11. Distribution of ethnic groups for individuals infected by each of the 154 subtype B phylotypes (n=24,100 with both included sequences and demographic data available) and across three VOIs. 21](#_Toc198199315)

[Figure S12. Distribution of the UK region of diagnosis for individuals infected by each of the 154 subtype B phylotypes (n=24,100) and across the three VOIs. 22](#_Toc198199316)

[Figure S13. Distribution of risk groups (or modes of HIV acquisition) for individuals infected by each of the 154 subtype B phylotypes (n=24,100) and across the three VOIs. 23](#_Toc198199317)

[Figure S14. Distribution of sex at birth for individuals infected by each of the 154 subtype B phylotypes (n=24,100) and across the three VOIs.. 24](#_Toc198199318)

[Figure S15. Ne(t) for the HIV-1 subtype B VOIs calculated using 50 grid points, the skygrid demographic model, and variations of the precision/smoothness (τ) parameter.. 25](#_Toc198199319)

[Figure S16. Exploratory analysis of post-treatment CD4 measurements according to VOI status (VOI, non-VOI, and backbone) for subtype B.. 26](#_Toc198199320)

[Figure S17. Distribution of TMRCA estimates of the three identified VOIs (PT.B.40.UK, PT.B.69.UK, and PT.B.133.UK) alongside 95% identity global matches for 10000 trees resulting from both Bayesian (BEAST) and ML (treedater parametric bootstrap) estimations. 27](#_Toc198199321)

[Figure S18. Global distribution over time of the VOI phylotypes (A) PT.B.40.UK, (B) PT.B.69.UK, and (C) PT.B.133.UK combined with their respective 250 random BLAST global matches at an identity threshold of 95%. 28](#_Toc198199322)

[Figure S19. Time-scaled tree of the pol gene for the main VOI phylotypes (PT.B.69.UK and PT.B.133.UK) similar to Figure 4A and C but also including ethnicity and years since diagnosis annotations.. 29](#_Toc198199323)

[Figure S20. Maximum likelihood tree of VOI PT.B.40.UK combined with 250 random BLAST global matches at an identity threshold of 95%.. 30](#_Toc198199324)

[Figure S21. Time-scaled tree of phylotypes from partial pol gene of VOI PT.B.40.UK. 31](#_Toc198199325)

[Supplementary tables 32](#_Toc198199326)

[Table S1. Demographic characteristics of all individuals from the UKRDB database for each of the more represented subtypes (B, C, A1, and CRF02_AG) and all others combined 32](#_Toc198199327)

[Table S2. Demographic characteristics of all individuals from the UKRDB database for each of the more represented subtypes (B, C, A1, and CRF02_AG) and all others combined after performing (i) sequence selection considering quality, pol minimum length, only for individuals while treatment naive, and non-molecular-clock outliers and (ii) CD4 data selection for individuals with matched sequences with at least two measurements before treatment initiation and passing additional CD4 filtering for outlying measurements and individuals. 34](#_Toc198199328)

[Table S3. Individual viral load and CD4 measurements over time in the absence of treatment for the defined VOIs 37](#_Toc198199329)

[Table S4. Number of *treestructure* phylotype assignments for different minimum clade sizes (30, 50, and 100) and bootstrap branch support values (90, 80, and without considering it) for the four analysed subtypes. The number of clusters resulting from the selected parameter choices of other partitioning methods (*fastbaps* and *treecluster*) are also presented. 50](#_Toc198199330)

[Table S5. *Treestructure* paraphyletic phylotype identifiers for the three minimum clades sizes and four subtypes tested.. 51](#_Toc198199331)

[Table S6. Welch’s one-sided t-test comparing the pre-treatment mean viral load (in log_10_ copies per ml) for individuals within their respective phylotypes against the backbone phylotype based on the minimum clade size = 30 *treestructure* designations. 52](#_Toc198199332)

[Table S7. Estimates of a Bayesian model that jointly estimates differences in viral load between *treestructure* (minimum clade size = 30) phylotypes considering all the data (*i.e.* not against the backbone phylotype).. 60](#_Toc198199333)

[Table S8. Phylotype regression coefficients (effect on slope, *i.e.* rate of CD4 decline per year) from the maximum likelihood mixed-effects model of CD4 cell decline with a random effect across different phylotypes (estimated using *treestructure*, minimum clade size = 30) and different individuals and fixed effects on risk group, age group, years since first CD4, sex, and relevant interactions of each phylotype against respective backbone phylotype.. 66](#_Toc198199334)

[Table S9. Phylotype regression coefficients (effect on slope, *i.e.* rate of CD4 decline per year) from the sensitivity analysis using a Bayesian mixed-effects model of CD4 cell decline with a random effect across different phylotypes (estimated using *treestructure*, minimum clade size = 30) and different individuals and fixed effects on risk group, age group, years since first CD4, sex, and relevant interactions of each phylotype against respective backbone phylotype. 72](#_Toc198199335)

[Table S10. Phylotype regression coefficients (effect on slope, *i.e.* rate of CD4 decline per year) from the sensitivity analysis using a Bayesian mixed-effects model of CD4 cell decline with a random effect across different individuals and fixed effects across different phylotypes using the R2-D2 prior (estimated using *treestructure*, minimum clade size = 30) and risk group, age group, years since first CD4, sex, and relevant interactions of each phylotype against respective backbone phylotype. 78](#_Toc198199336)

[Table S11. Phylotype regression coefficients (effect on slope, *i.e.* rate of CD4 decline per year) from the Bayesian linear mixed-effects model of CD4 cell decline against the respective backbone phylotype on suspected VOIs highlighted by the ML CD4 decline model with a random effect on phylotypes (Table S8) and corroborated by the sensitivity analysis (Tables S9 and S10). In this model, phylotypes (estimated using *treestructure*, minimum clade size = 30) are modelled as having fixed effects on slopes and intercepts. 84](#_Toc198199337)

[Table S12. Absolute number and proportion of VOIs across the 24,100 subtype B sequences analysed in this study before and after (including) 2015. 85](#_Toc198199338)

[Table S13. Results of other covariates of the Bayesian fixed effects CD4 decline model for the six subtype B variants with significantly faster CD4 decline (see table S11).. 86](#_Toc198199339)

[Table S14. Welch’s two-sided t-test comparing the mean age at diagnosis for individuals within VOI subtype B phylotypes against the backbone phylotype (ID=153).. 88](#_Toc198199340)

[Table S15. Major drug-resistance nucleotide mutation (and respective non-synonymous amino acid change) in high frequency (≥75%) for the VOI phylotypes.. 89](#_Toc198199341)

[Table S16. Percentage of sequences within subtype B phylotypes with high and intermediate scores of drug-resistance (red and yellow, respectively) according to the Stanford University HIV Drug Resistance Database (HIVdb) searched using sierra-local for eight PR and 12 NRTI/NNRTI inhibitors. 90](#_Toc198199342)

[Table S17. Bayesian model of CD4 post-treatment measurements with a fixed effect on phylotype. The key difference (when compared to the model described in Methods: Linkage between genetic data and CD4 counts and presented in Table S11) is that in this case we are investigating post-treatment CD4 increase (replenishment). 91](#_Toc198199343)

[Table S18. BLAST similarity search of subtype B VOI phylotypes against a reference database of 167,710 HIV-1 subtype B sequences from the LANL database. 92](#_Toc198199344)

[References 93](#_Toc198199345)

##

## **Supplementary text**

### Text S1. Bayesian joint viral load model variables and prior selection

In order to choose weakly but still relatively informative priors—that would also be conservative to detect viral load differences—for the key parameters of the Bayesian joint viral load model, we used the following empirical approach as follows. We first ran the RStan Bayesian joint viral load model for the four subtypes and 3 treestructure minimum clade sizes (30, 50, and 100). We used default uniform priors defined by the specified parameter bounds—for example, Uniform(-10, 10) for $y_{mean\_pop}$(the population-level mean viral load, in log_10_ copies/mL) and Uniform(0, 10) for both $y_{sd}$(the standard deviation [SD] of the residuals across observations, in log_10_ copies/mL) and $y_{sd\_group}$(the SD of phylotype-specific effects, also in log_10_ copies/mL). The phylotype-specific effects, $phylotype\_effects\_unscaled_{[j]}$, were assigned a Normal(0,1) prior. Based on the 12 combinations of subtypes and treestructure thresholds, we visually inspected the posterior distributions of these key parameters and crudely determined mean and SD for these parameters. These were then set as the priors for these parameters for all subtypes and treestructure thresholds. They were specified as follows:

$y_{mean\_pop}\sim N(4.5,1)$ subject to $2 \leq y_{mean\_pop} \leq7$

$y_{sd} \sim N(0.85, 0.05)$ subject to $y_{sd} \geq0$

$y_{sd\_group} \sim N(0.05, 0.3)$subject to $y_{sd\_group} \geq0$

$phylotype\_effects\_unscaled_{[j]} \sim N(0,1)$. This distribution ensures that phylotype effects are centered around the population mean.

### Text S2. Bayesian joint CD4 decline model with a random effect on phylotype slopes and intercepts

For the Bayesian joint CD4 decline model with a random effect on phylotype slopes and intercepts, we specified a normal (mean = 0 and SD = 20 cells/mm^3^/year) for slopes and normal (mean = 0 and SD = 50 cells/mm^3^) for intercepts. We placed a Lewandowski–Kurowicka–Joe (LKJ) prior with a concentration parameter of 2 on the correlation matrices of random intercepts and slopes for both phylotypes and individuals, which modestly favours correlations near zero while still allowing moderate and strong correlations if supported by the data. We implemented this model in brms v2.19.0 (Bürkner 2017), with two independent chains of 10,000 MCMC iterations, discarding 50% as burn-in, and assessing convergence using R-hat < 1.01 and ESS > 400.

### Text S3. Bayesian joint CD4 decline model with a fixed effect on phylotype intercepts and slopes using a R2-D2 prior

For the Bayesian joint CD4 decline model with a fixed effect on phylotype intercepts and slopes, the R2-D2 shrinkage prior — which was applied in all fixed effects — was configured with an expected mean marginal R^2^ given by the ML random effect model (e.g. 15.5% in the case of the subtype B and minimum clade size of 30), precision of 2 (relatively weak belief around mean R^2^), and a Dirichlet concentration parameter of 0.5, which controls the prior concentration of effect sizes and applies aggressive shrinkage on coefficients with low relevance. We also ran this model in brms v2.19.0 (Bürkner 2017), with two independent chains of 5,000 MCMC iterations, discarding 40% as burn-in, and assessing convergence using R-hat < 1.01 and ESS > 400.

### Text S4. Stratification of ethnicity, risk group, region of diagnosis, and age

Regarding sociodemographic variables, sex is defined as sex assigned at birth (male or female). Ethnicity was grouped into eight classes: White; Black Caribbean; Black African; Other or unspecified black; Indian, Pakistani, or Bangladeshi; Other Asian or Oriental; Other and mixed; Other. These denominations were simplified into five categories in tables due to the absence of a substantial number of individuals in some ethnic groups. Risk groups were categorised as: Homo/bisexual; Heterosexual; IDU; Blood products; Others/unknown. IDU and blood products were also combined in tables. The region of diagnosis was organised into five classes: South of England; London; North of England; Midlands and East of England; Northern Ireland, Scotland, and Wales. Age at diagnosis was calculated relative to year of birth. Age groups were defined relative to age at diagnosis and were categorised into five levels: <29, 30-39, 40-49, 50-59, and >59. Additionally, dates of diagnosis, sampling, viral load measurements, and CD4 episodes, all of which had a month's precision, were rounded to the 15^th^ day of the respective month and year.

### Text S5. Whole-genome sequence analysis

We collated and trimmed INITiO (recent infections) and COMPARE (Brighton cohort) sequences from the UK Health Security Agency (UKHSA) to their pol domains using nhmmer v3.3.2 (Wheeler & Eddy 2013). We then performed BLAST v2.12.0 (Altschul et al. 1990) searches against a subtype-annotated reference database and those matching subtype B were retained and binned. Subsequently, we aligned the UKHSA and VOI phylotype (PT.B.40.UK, PT.B.69.UK, and PT.B.133.UK) sequences against the aligned reference sequences for subtype B using WITCH v1.0.4 (Shen et al. 2022), and trimmed those to match the VOI sequence fragments. We also calculated a distance matrix containing all pairwise Poisson distances between sequences. This process quantifies the dissimilarity between sequences by comparing the mutation counts in each sequence and assessing the probability that the observed differences could arise under a Poisson distribution model. We retained all references, all VOI phylotype sequences, and the 200 UKHSA closest matches to the VOIs, and built guide trees using FastTreeMP v2.1.11 (Price et al. 2010). Finally, we estimated ML trees for each VOI and their respective closest UKHSA matches with IQ-TREE v2.2.0.3 (Minh et al. 2020) using a General Time Reversible (GTR) (Tavare 1986) model with 6 rate categories, 1000 ultrafast bootstraps, 2000 iterations, and convergence checks after every 100 iterations. We could divide the output trees based on the phyletic status of the VOI sequences. In cases where a single branch (monophyletic clade) grouped all VOI sequences, we recorded the UKHSA sequences clustering therein.

We extracted the UKHSA whole-genome sequences (WGS) forming a monophyletic clade with the VOI pol sequences from the VOI-specific trees, and added the HXB2 reference strain to the existing alignments using the *--addfragments* parameter in MAFFT v7.490 (Katoh & Standley 2013). Subsequently, we ensured the ungapped length of the alignments was the same as the HXB2 reference (9719 nucleotides), and masked regions of poor genomic coverage. We then performed variant calling to identify synonymous and non-synonymous mutations, insertions, and deletions using gofasta (Jackson 2022) variants v1.2.1 and a custom GenBank file annotation for the HXB2 reference. We loaded and summarised the CSV outputs containing the identified mutations in R v4.1.3. The same procedure of variant calling against the HXB2 reference was performed for a consensus sequence of the WGS cohorts analysed. This consensus was generated in the same way as consensus phylotype sequences. This analysis allowed us to flag mutations relative to HXB2 that are rare or enriched not only in the LANL global database, but also in the analysed dataset (see Data S1).

We did not find any close matches to PT.B.69.UK. We obtained 16, one, and two WGS for PT.B.40.UK, PT.B.133.UK, and PT.B.137.UK, respectively (Fig. S5). PT.B.137.UK (despite not being initially classified as VOI) was included in this case because it is closely related to PT.B.69.UK (**Fig. 1**) and is significant in one viral load (T-test, **Table S7**) and both CD4 (**Tables S8 and S11**) analyses. The total number of mutations across all sequences in each VOI (for this specific analysis PT.B.40.UK, PT.B.133.UK, and PT.B.137.UK) subset was 3066, 756, and 737, respectively. The number of defining mutations (found in >80% of the WGS sequences for each VOI) was 219, 756, and 737. Evidently, mutations from PT.B.133.UK and PT.B.137.UK are expected to be less reliable due to the presence of only one and two WGS. Of the above, there are 95 putative defining mutations not found in the consensus of the analysed WGS cohorts for PT.B.40.UK, 607 for PT.B.133.UK, and 366 for PT.B.137.UK.

To search for potential convergent genetic features across these four VOIs, we merged common defining mutations of all of them and investigated the presence of drug resistance mutations (DRMs) tabulated from the Stanford HIV Drug Resistance Database (HIVdb 2024), cytotoxic T lymphocytes (CTL) escapes (Carlson et al. 2012), and overlapping mutations with the highly virulent VB variant (Wymant et al. 2022). By performing amino acid motif searches against the LANL global HIV database (<https://www.hiv.lanl.gov/components/sequence/HIV/search/search.html>) for the 44 non-synonymous mutations shared by the three VOIs, we identified a few rare mutations in subtype B, including gag:S465F, pol integrase:G123S, and at least four close env_gp120 mutations (V318Y, I320T, K322E, and N325D) (in bold in **Data S1**). We also joined common mutations of VOIs in different combinations, and of each one individually, but no major differences were observed as compared to the combination of mutations across the three VOIs. When differences occurred (*i.e.* additional sites detected to be CTL escapes or mutations overlapping the VB variant sites), their log odds for escape were not extreme and they were not particularly rare in subtype B (i.e., representing a novel mutation signature) as indicated by the motif searches.

### Text S6. Coreceptor usage prediction

Given the association between the switch in coreceptor usage from CCR5 to CXCR4 and faster disease progression (Connor et al. 1997), we used the geno2pheno (coreceptor) algorithm (Lengauer et al. 2007) to compare the proportions of X4 and R5-capable viruses from backbone and subtype B VOI V3 loop sequences. This analysis included 89 backbone and 30 sequences from the three subtype B VOIs. A sample is predicted as X4-capable if its False Positivity Rate (FPR) is below the FPR cutoff (20% for single sequences as recommended by the European guidelines, Vandekerckhove et al. 2011). The backbone sequences presented a median of 38% FPR (IQR: 10-59%) while the VOI sequences had 41% (IQR: 11-65%). The percentage of predicted X4-tropic viruses was, respectively, 38% and 33%. We also tested for a difference in the distribution of FPRs between these two groups using a Wilcoxon rank sum test and performed a Beta regression to quantify the relationship between VOI membership and these FPR values.

### Text S7. The UK HIV-1 epidemic through the lenses of UKRDB

The UK HIV-1 epidemic through the lenses of UKRDB is particularly dominated by male individuals (56.5%), white (44.4%), homo/bisexual (39.7%), people diagnosed in London (38.0%), and diagnosed at the age of 30-39 years (29.9%), although there are marked differences, particularly between subtype B and the other subtypes (**Table S1**).

Overrepresentation of some demographic groups is observed when comparing the UKRDB complete database and the analysed data resulting from sequence and treatment-naive filters, and CD4 data selection (**Table S2**). When compared to **Table S1** (all UKRDB individuals), there is an overrepresentation (13-22% difference) of male, white, homo/bisexual PLWH for subtype B. For subtype C, black (Caribbean/African/other) and heterosexual populations have a higher proportion of analysed PLWH than usually found in the database (*e.g.* a representation of 71.6% heterosexuals for the complete database becomes 91.8% after sequence filters). There is also an asymmetry in the representativity of individuals from London across all subtypes, especially regarding CD4 data availability, where usually there are ~27-32% more individuals included than the percentage observed in the database (**Table S2**).

### Text S8. Examples of sociodemographic structure across non-VOI phylotypes

Some non-VOI subtype B phylotypes show differences in ethnic composition. Notably, PT.B.83.UK, PT.B.146.UK and PT.B.148.UK are more common in Black-Caribbean. Additionally, PT.B.86.UK, PT.B.92.UK, and PT.B.108.UK are distributed more widely in different ethnic groups, including especially Black and Asian backgrounds (**Fig. S11A**). Interestingly, PT.B.17.UK is a phylotype almost only found in injecting drug users (IDU), and PT.B.146.UK is predominant in heterosexual individuals (**Fig. S13A**). PT.B.146.UK also has a >75% of females (**Fig. S14A**).

Altogether, these distributions of PT.B.146.UK variables suggest a sociodemographic outlier given that this phylotype is composed mostly of Black-Caribbean heterosexual females living in London (**Figs. S11-S14**).

### Text S9. DRMs and resistance scores

For the 154 subtype B phylotypes, we inspected DRMs and resistance scores using sierra-local (Ho et al. 2019) and HIVdb version 9.4 (last updated in December 7, 2022) to search for potential high and intermediate levels of resistance against eight PR and 12 (non-) nucleoside/nucleotide reverse transcriptase (NNRTI/NRTI) inhibitors.

We identified only one major DRM across all VOIs: PR:L90M from PT.B.40.UK. It is found in 86.4% of the sequences of this phylotype, while not found in >75% frequency in any other UK subtype B phylotype (**Table S15**). This DRM is primarily selected by SQV, NFV, IDV, and ATV, and contributes to reduced susceptibility to ATV and LPV (HIVdb 2022). Additionally, we did not detect widespread enrichment for intermediate and high levels of resistance across VOIs. However, PT.B.40.UK had most sequences resistant to three protease inhibitors (IDV, NFV, and SQV). PT.B.69.UK had a small number of sequences resistant to the NRTIs / NNRTIs inhibitors EFV and NVP, which results from the presence of mutation RT:K103N. Most interestingly, the non-VOI PT.B.140.UK presented ≥10% of its sequences resistant to both classes of antiretrovirals (**Table S16**).

### Text S10. Treatment failures

A total of 3802 post-treatment measurements across 1738 individuals from subtype B were successfully cross-referenced with sequence data. From these, n=12 individuals (32 CD4s) from PT.B.40.UK, n=1 (2 CD4s) from PT.B.69.UK , and n=3 (6 CD4s) from PT.B.133.UK. The backbone phylotype had n=1105 individuals (2408 CD4s). We firstly performed an exploratory analysis by VOI status (VOI, non-VOI, and backbone) on individual CD4 slopes and their distribution across these three VOI statuses. The distribution of slopes for the backbone and non-VOI groups closely match, while VOI distributions have one peak at declining and another at increasing slopes (**Fig. S16**).

Despite these small sample sizes, we attempted to investigate and potentially identify if treatment failures could be increased in VOIs by using the same Bayesian fixed effects model (see **Methods: Linkage between genetic data and CD4 counts**). The model was fitted using VOI and backbone individuals only. As initially suspected, the model estimates for VOIs presented wide credible intervals (**Table S17**). The estimated difference in the rate of CD4 increase between VOIs and the backbone was small—only about 5 cells/mm³/year—indicating that VOIs have a similar CD4 increase (approximately 100 cells/mm³/year) to the backbone (95 cells/mm³/year), and that treatment is likely working as expected. Nevertheless, the pronounced uncertainty around these estimates prevents us from reaching a definitive conclusion about the hypothesis that these VOIs with increased virulence are more likely to result in treatment failures.

## **Supplementary figures**


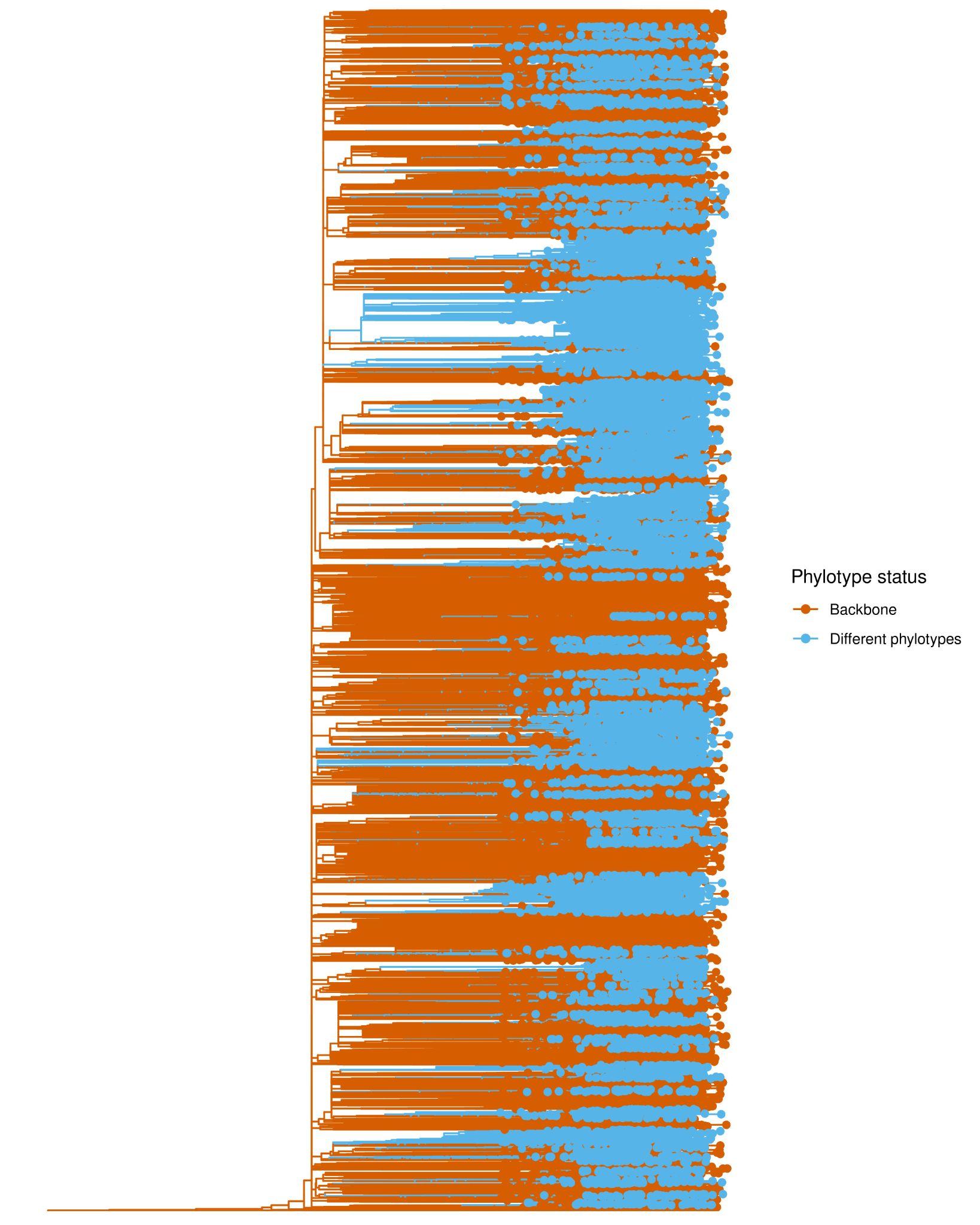


### Figure S1. Example of *treestructure* output for subtype B, minimum clade size of 30, and BT = 80. It demonstrates the existence of the backbone phylotype (orange), which includes the root and likely resembles the ancestral state of the virus, against which individual phylotypes are compared in viral load and CD4 statistical analyses. In this example, light blue (different phylotypes) indicates the other 153 phylotypes that were merged for visualisation purposes.


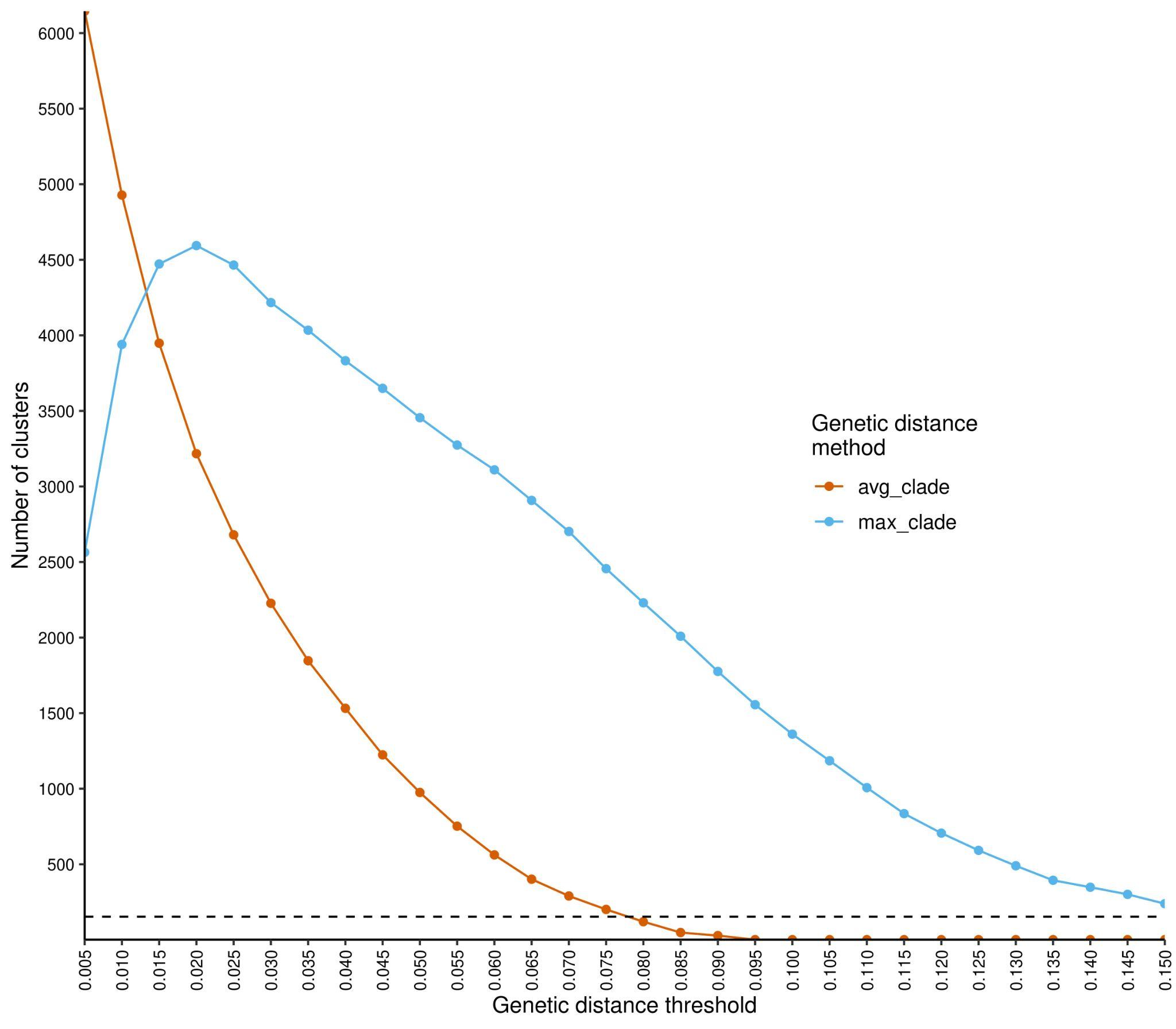


### Figure S2. Variation in the number of clusters detected by *treecluster* per genetic distance threshold for the two most common genetic distance methods and considering bootstrap support = 80% for subtype B sequences. The horizontal dashed line represents the number of clusters detected by *treestructure* using minimum clade size = 30 and bootstrap support = 80, which is equal to 153 in this case.


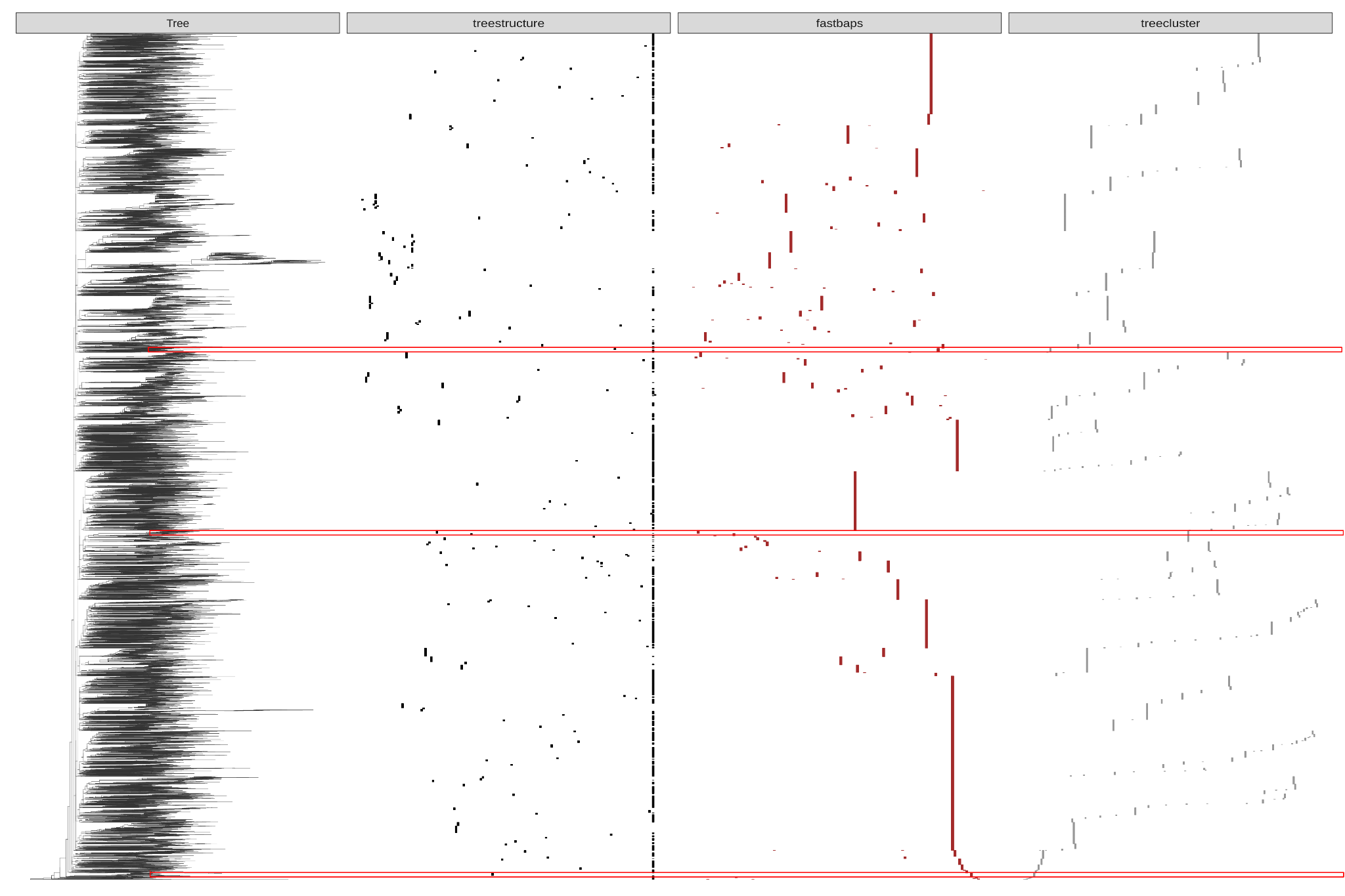


### Figure S3. Comparison of clusters/phylotypes given by different partitioning methods for subtype B UKRDB partial *pol* sequences. The leftmost panel shows the ML tree built from 24,100 sequences. The second panel shows the 154 phylotypes detected by *treestructure* (minimum clade size = 30 and bootstrap support = 80%). The third panel depicts clusters detected by *fastbaps* (optimise.symmetric hyper prior). The rightmost panel shows clusters detected by *treecluster (*avg_clade method, 0.075 [*i.e.* 7.5% genetic distance], and bootstrap support = 80%*). Fastbaps* and *treecluster* outputs were chosen so that they more closely resembled the number of partitions/phylotypes given by *treestructure* (*e,g,* see Figure S2 below). Each horizontal small panel indicates different partitions, and sequences in the same vertical position belong to the same partition. The VOI phylotypes and their respective assignments are indicated by red rectangles. From top to bottom these are PT.B.133.UK, PT.B.40.UK, and PT.B.69.UK


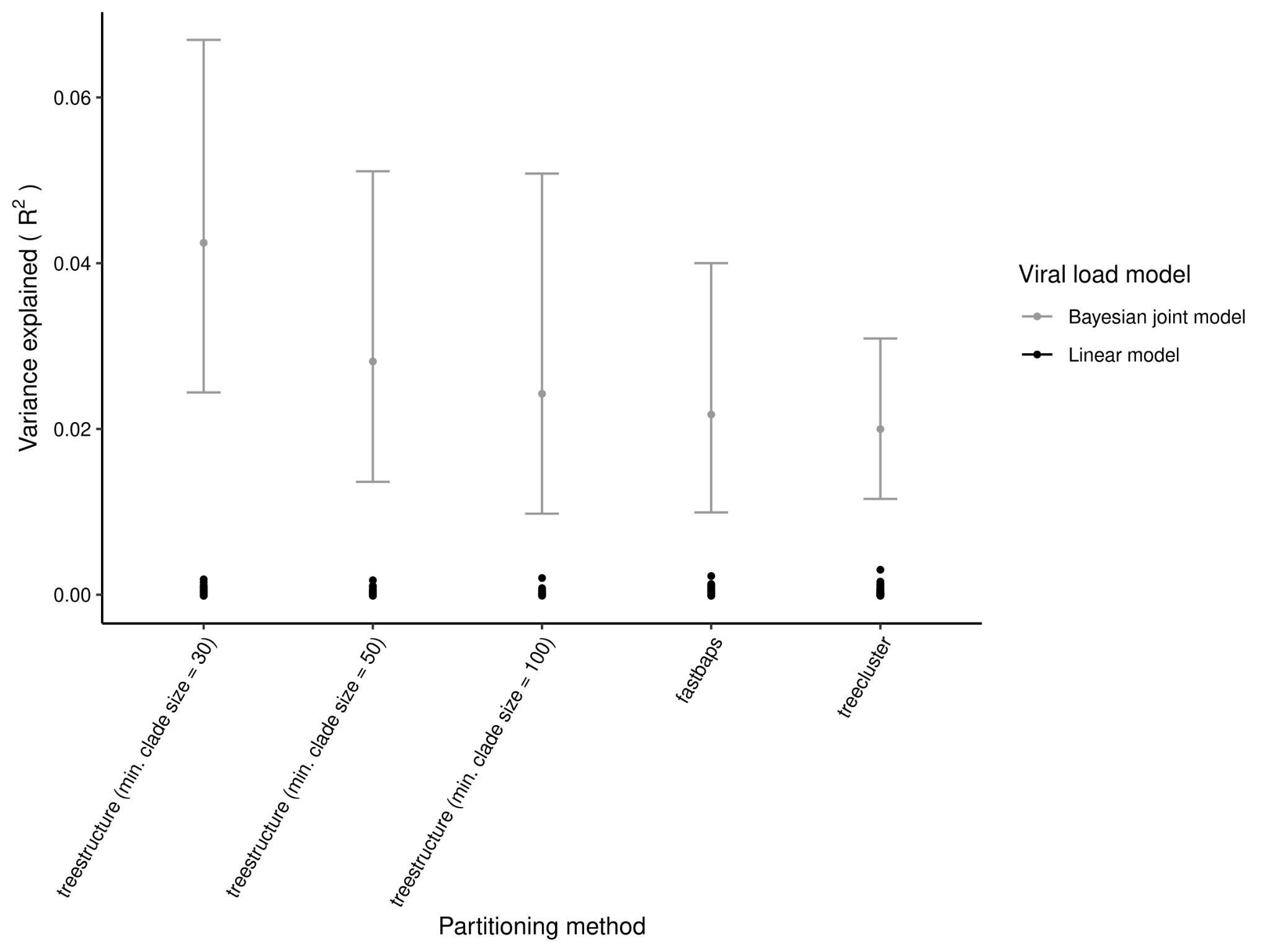


### Figure S4. Comparison of the proportion of variance (R^2^) in viral load explained by different partitioning methods and among choices of minimum clade size of *treestructure* for subtype B sequences. To calculate the proportion of variance in viral load explained by phylotype effects in the Bayesian joint model, we used posterior samples of the residual standard deviation (y_sd) and the group-level (phylotype) standard deviation (y_sd_group). For each posterior draw, we computed the variance explained by phylotypes as the squared group-level SD, and the total variance as the sum of group-level and residual variances. The Bayesian R^2^ was then defined as the ratio of group-level variance to total variance. We summarised this across samples by reporting the median and 95% credible interval. This approach reflects the proportion of variance attributable to phylotypes while accounting for posterior uncertainty. For the simple linear model, these are the default *lm()* summary output of adjusted R^2^ for each phylotype individually against the backbone. This metric is computed by multiplying the unexplained proportion of variance (i.e., 1− R^2^) by a factor that increases with the number of predictors and decreases with the sample size. This adjusted value is then subtracted from 1 to yield the adjusted R^2^.


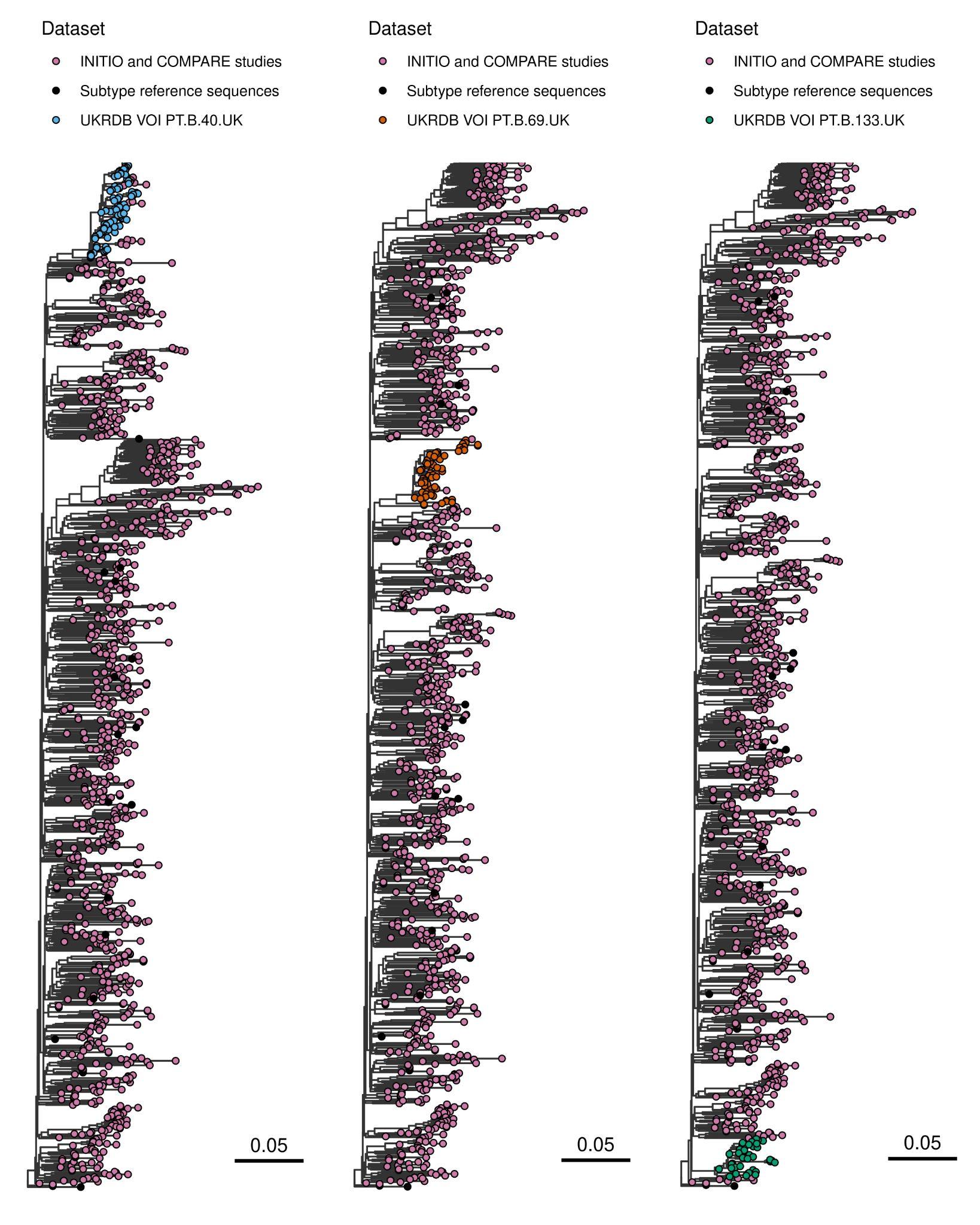


### Figure S5. Maximum likelihood phylogenetic trees of partial pol sequences from four UKRDB subtype B VOI phylotypes (PT.B.40.UK, PT.B.69.UK, and PT.B.133.UK) combined with INITiO and COMPARE cohort sequences. Sequences from these cohorts composing a monophyletic clade with the UKRDB phylotype VOIs were retained for whole-genome sequence analysis.

###


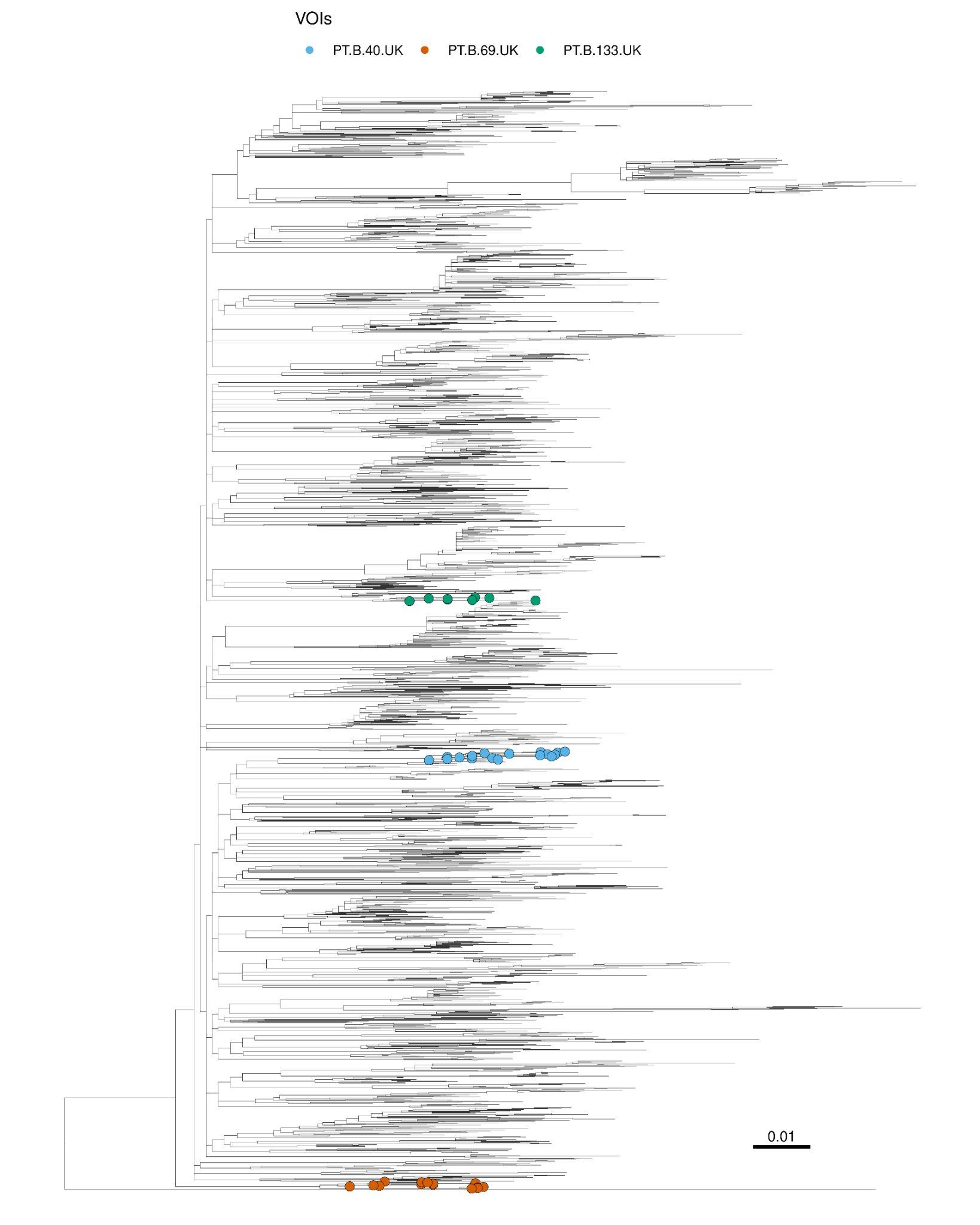


### Figure S6. Maximum likelihood phylogeny of 2051 subtype B partial pol sequences subsampled from the original tree of 24,101 sequences from which the phylotypes with a minimum size of 30 were computed with *treestructure*. The number of tips for each of the 154 phylotypes was selected according to their sizes (total size of phylotype divided by 5, *i.e.* 20% of sequences) for visualisation purposes. Tips of VOI phylotypes are coloured.

##


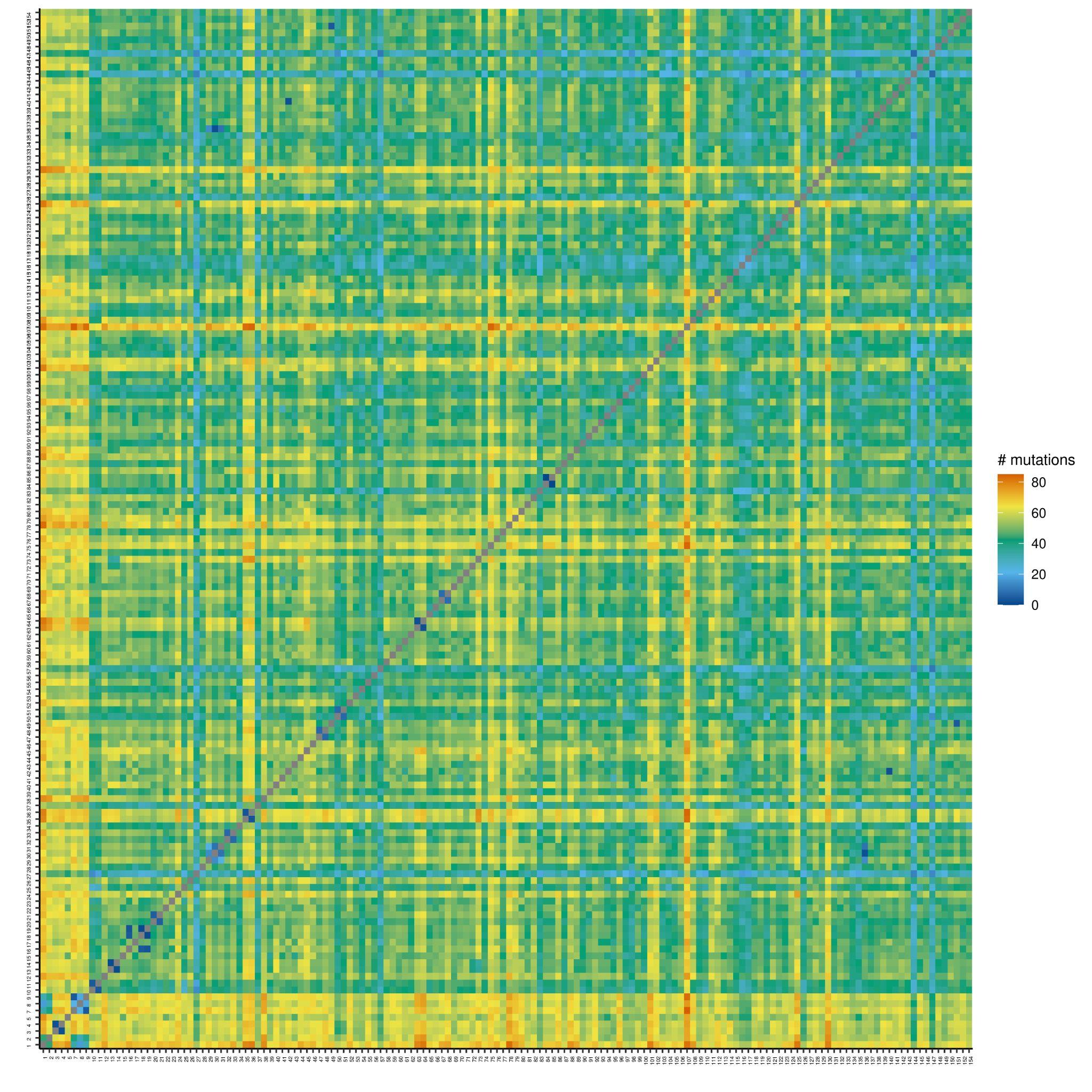


### Figure S7. Nucleotide-level distance matrix of consensus sequences of the 154 subtype B phylotypes for a 995-length *pol* (complete protease and partial reverse transcriptase) alignment.


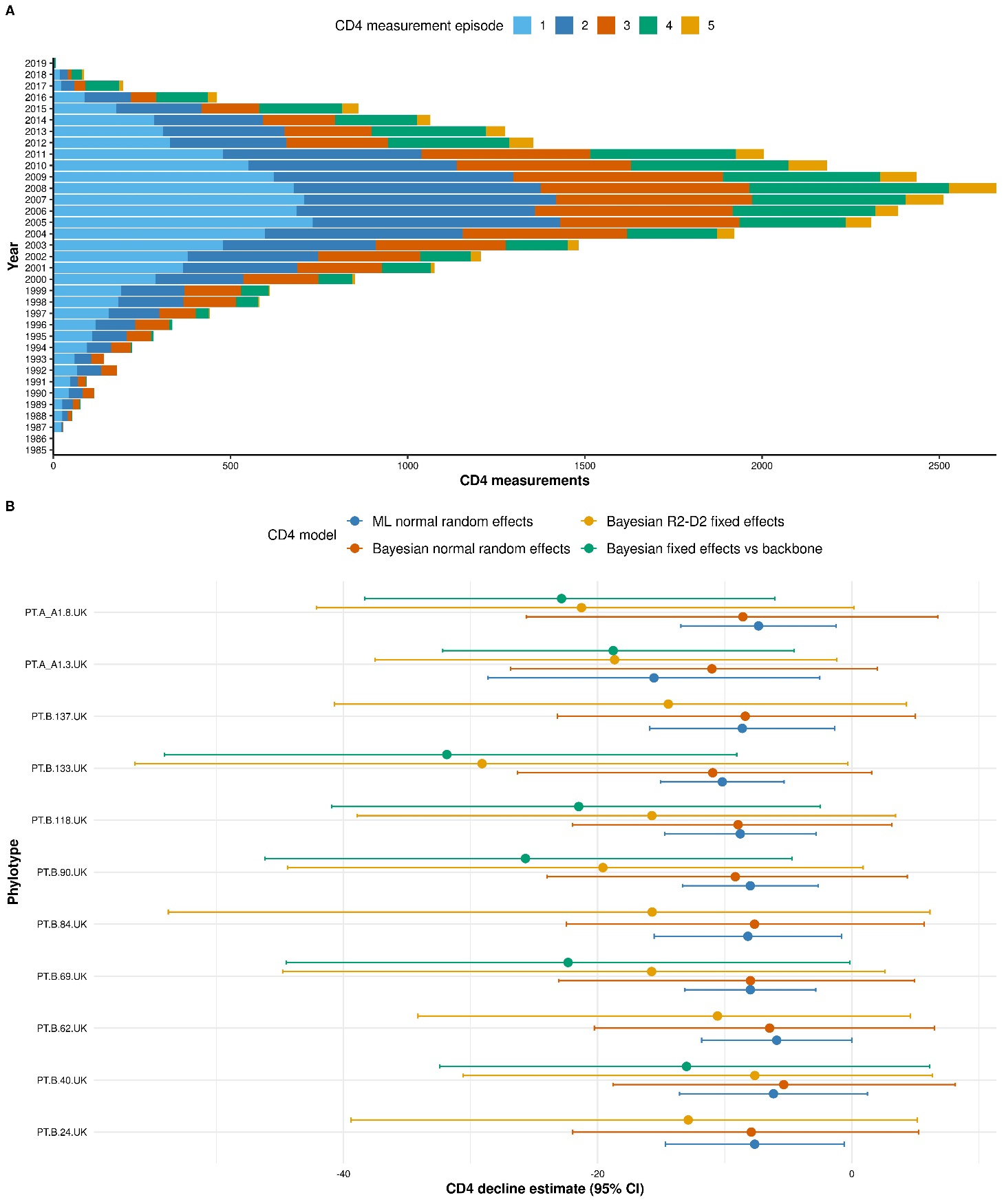


### Figure S8. Overview of CD4 measurements and differences between the four CD4 decline models investigated. (A) Absolute number of CD4 measurements pre-treatment per year coloured by measurement episode (*i.e.* nth measurement over the course of individual’s infection) across the 8946 individuals (and 31,503 total CD4 measurements) analysed that were infected with subtype B virus. (B) Estimates from the four CD4 decline models for n=11 main phylotypes (n=9 subtype and n=2 subtype A1), defined as the intersection of phylotypes with p-value < 0.05 from the ML D4 model and PP > 80% in the two Bayesian sensitivity analysis models. The VOI based on viral load (PT.B.40.UK) is also included.

##


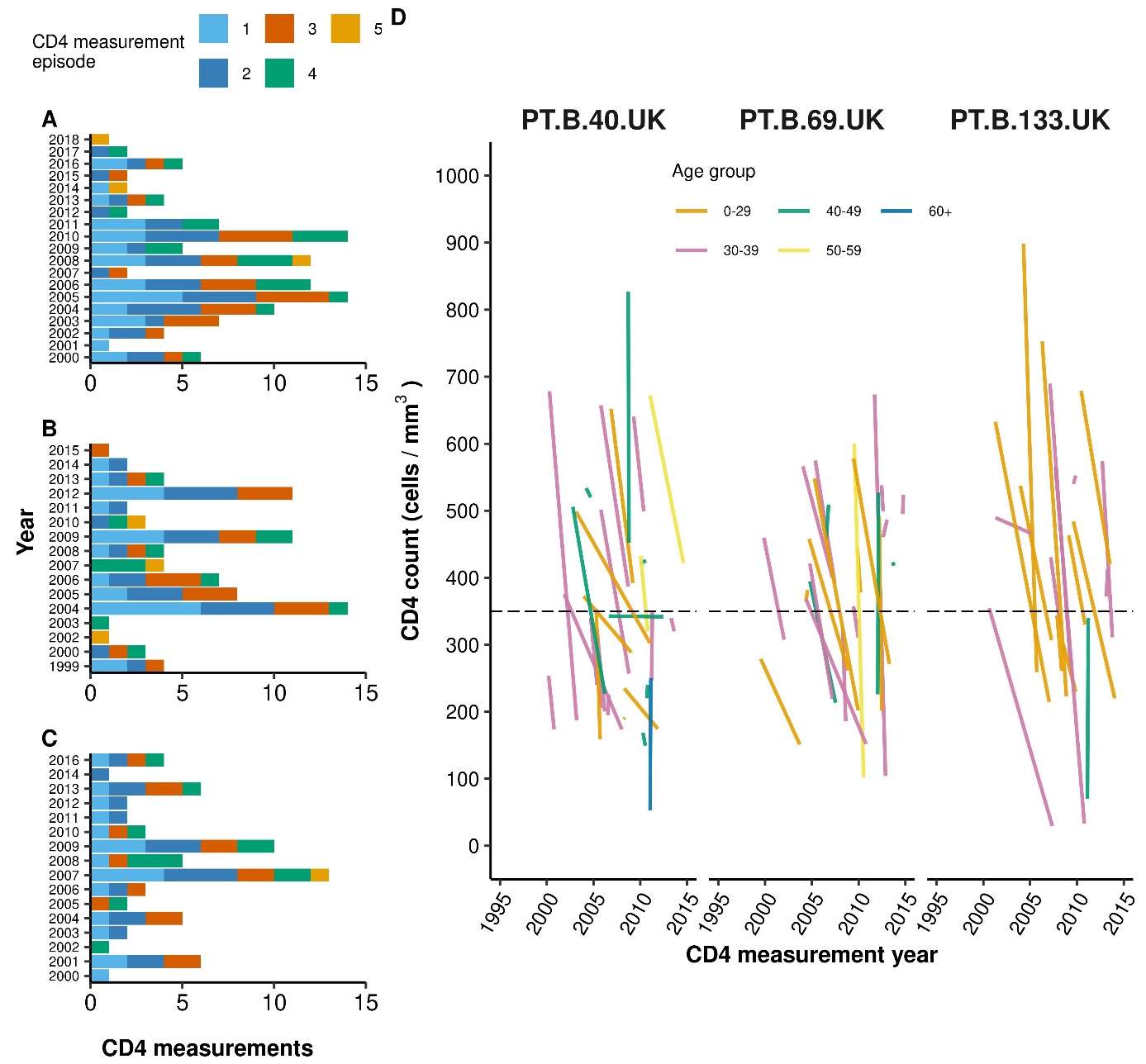


### Figure S9. (A-C) Absolute number of CD4 measurements pre-treatment (for individuals with more than one measure) per year coloured by episode per individual within the VOIs (A) PT.B.40.UK, (B) PT.B.69.UK, and (C) PT.B.133.UK. (D) Pre-treatment CD4 declining slopes for each individual infected by the VOIs coloured by age group. The dashed line indicates CD4 = 350 cells/mm^3^.


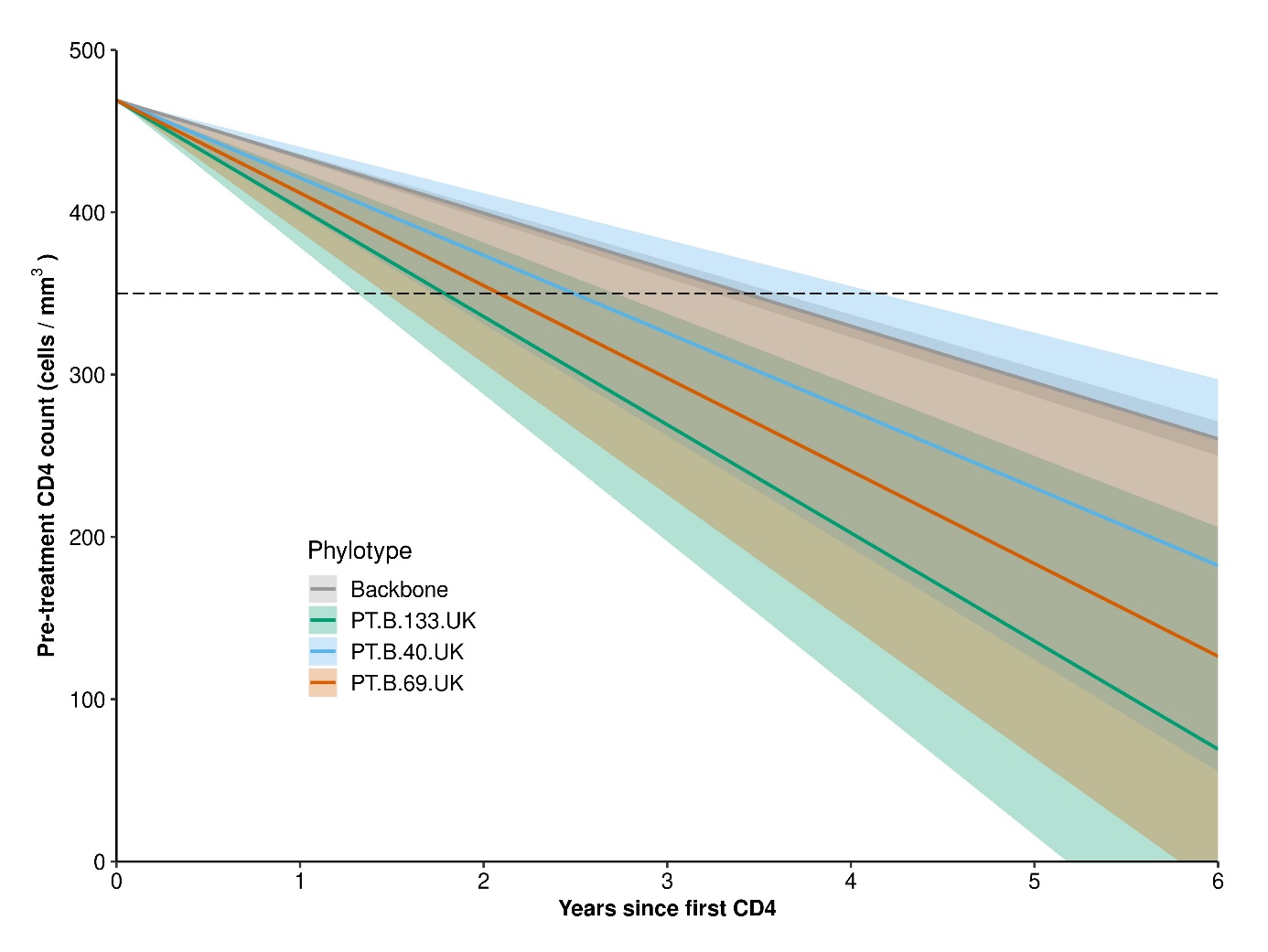


### Figure S10. Expected decline in CD4 (measured in cells per mm^3^ of blood) in the absence of treatment as measured by the fixed effects mixed Bayesian model against the backbone phylotype. This assumes an initial CD4 measurement of 469 cells/mm^3^ (intercept estimated for the reference group) and is presented alongside Fig. 2B given the uncertainty around phylotype-specific baseline CD4 counts (intercepts). 95% CIs of the slopes (rate of CD4 decline per year) are represented by shaded regions. PT.B.40.UK is presented for comparison despite only displaying elevated viral loads.


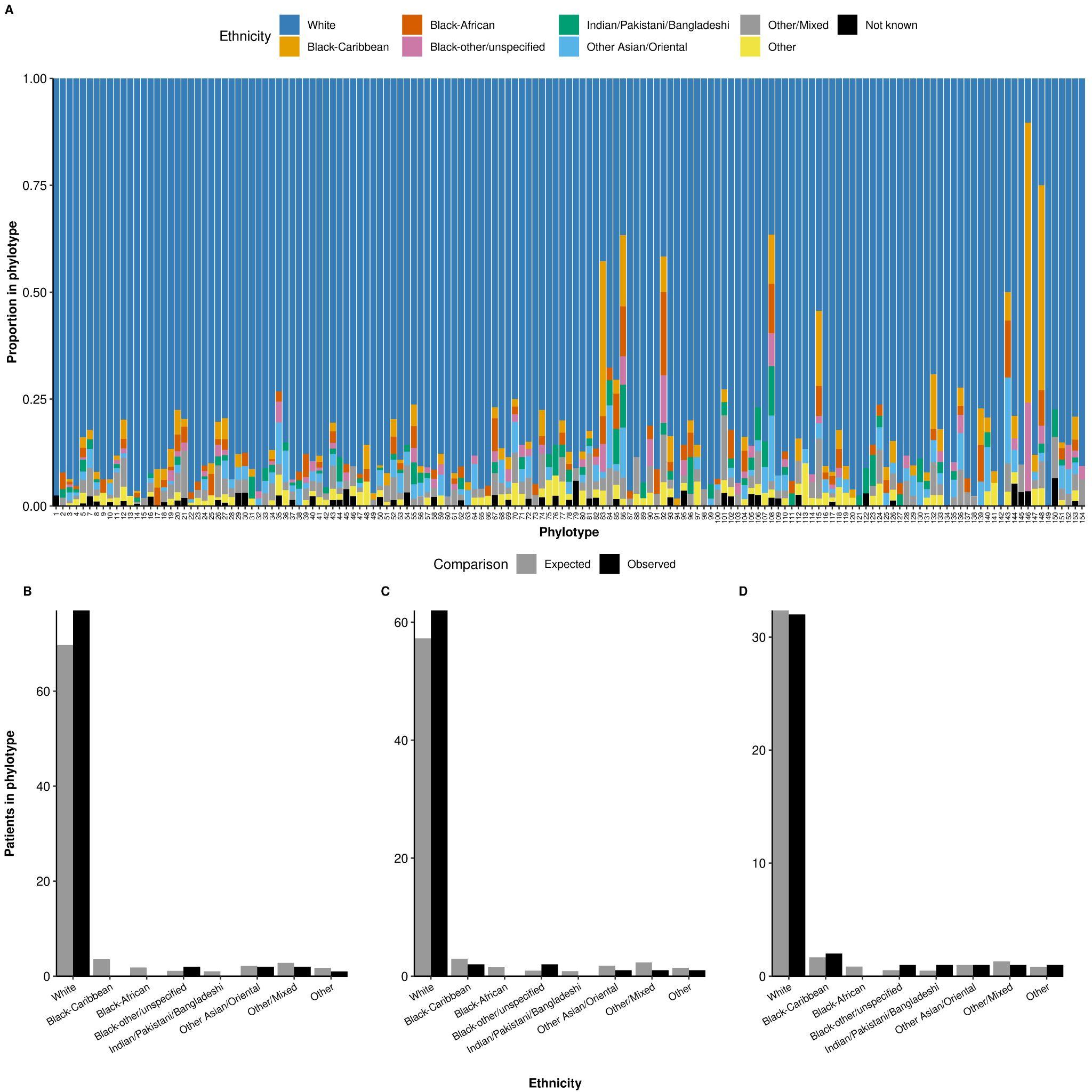


### Figure S11. Distribution of ethnic groups for individuals infected by each of the 154 subtype B phylotypes (n=24,100 with both included sequences and demographic data available) and across three VOIs. (A) Percentage of people per ethnic group within each phylotype. (B) Expected under null *vs* observed distributions of ethnicity categories given by Chi-squared test for phylotypes (B) PT.B.40.UK, (C) PT.B.69.UK, and (D) PT.B.133.UK.


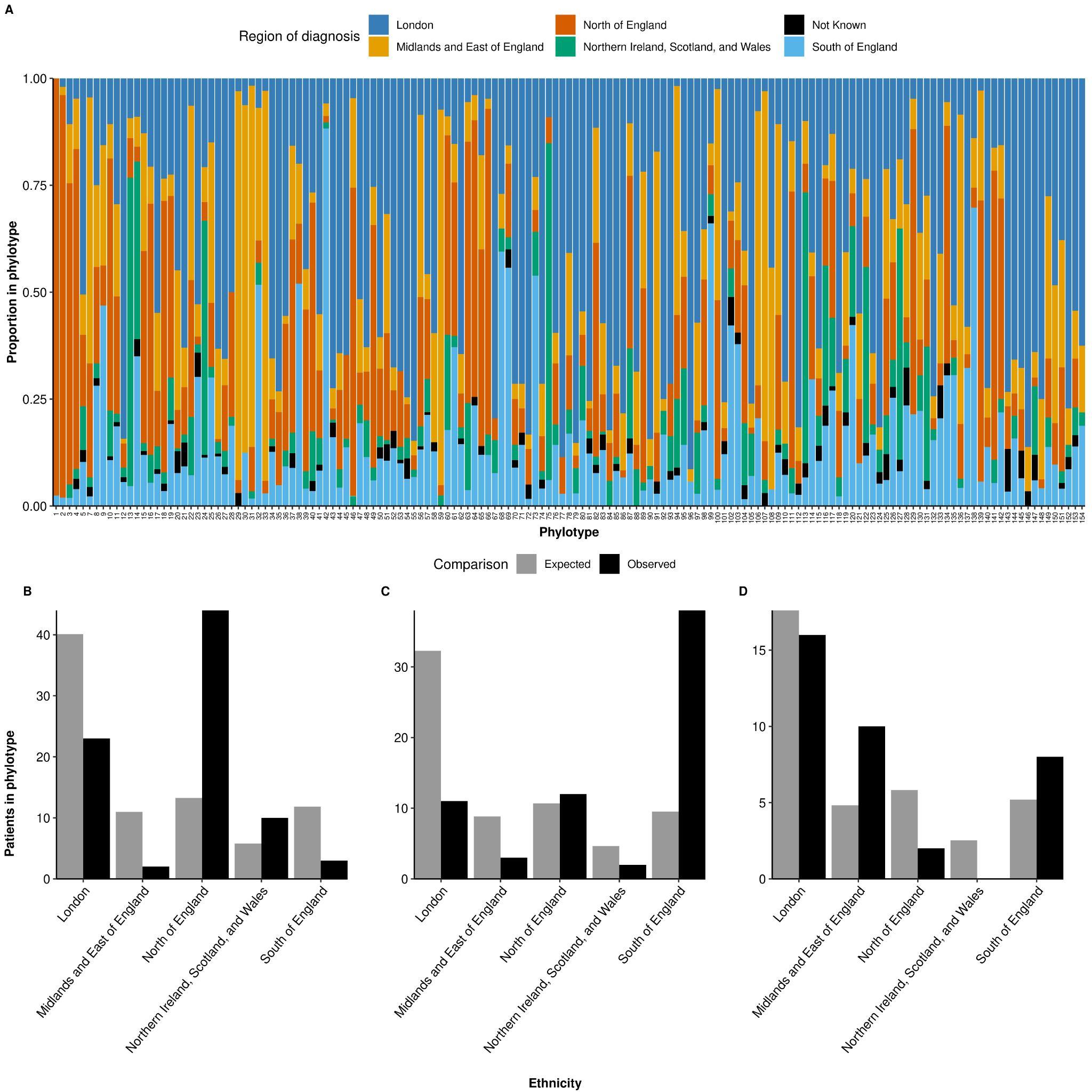


### Figure S12. Distribution of the UK region of diagnosis for individuals infected by each of the 154 subtype B phylotypes (n=24,100) and across the three VOIs. (A) Percentage of people per region of diagnosis within each phylotype. (B) Expected under null *vs* observed distributions of region categories given by Chi-squared test for phylotypes (B) PT.B.40.UK, (C) PT.B.69.UK, and (D) PT.B.133.UK.


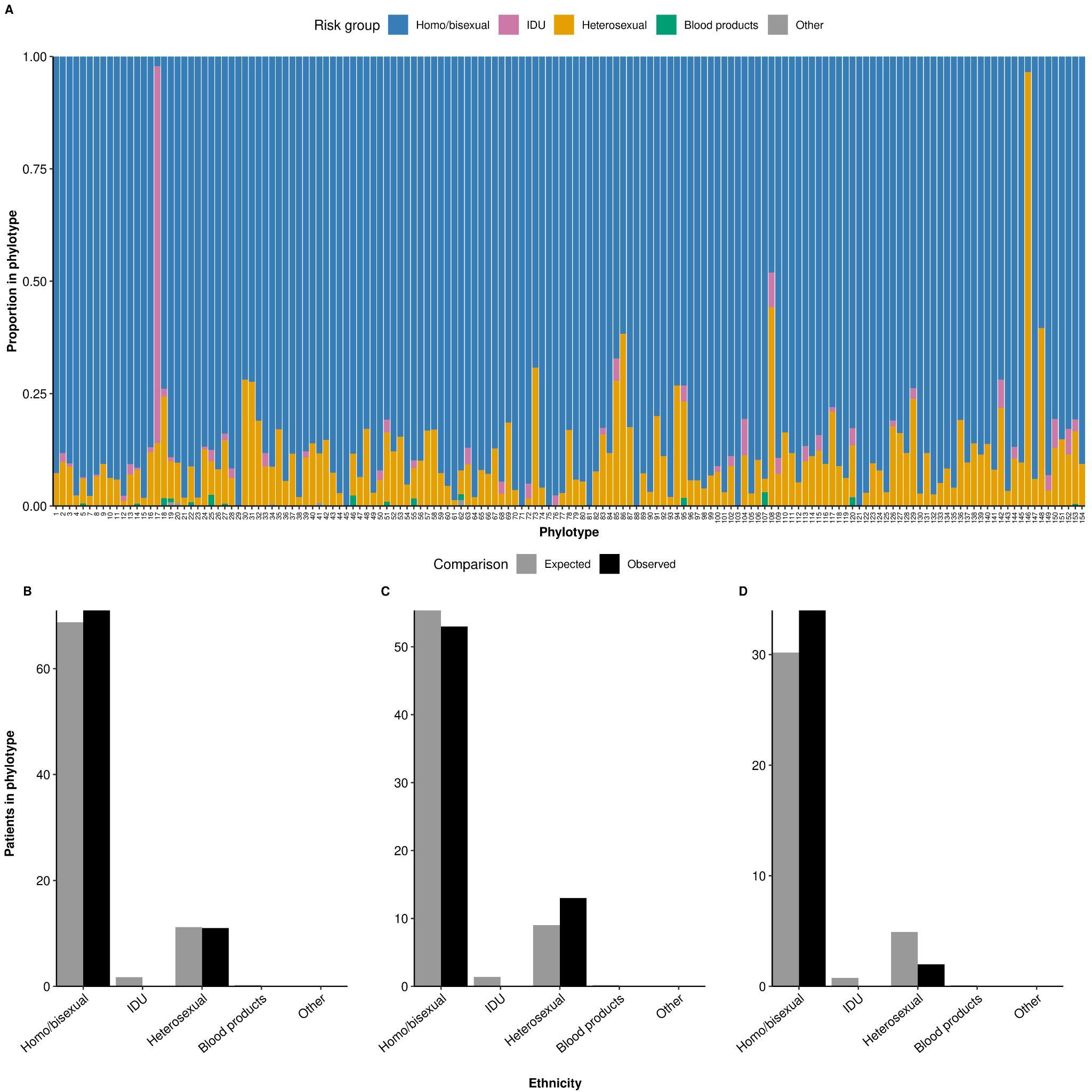


### Figure S13. Distribution of risk groups (or modes of HIV acquisition) for individuals infected by each of the 154 subtype B phylotypes (n=24,100) and across the three VOIs. (A) Percentage of people per risk group within each phylotype. (B) Expected under null *vs* observed distributions of risk group categories given by Chi-squared test for phylotypes (B) PT.B.40.UK, (C) PT.B.69.UK, and (D) PT.B.133.UK.


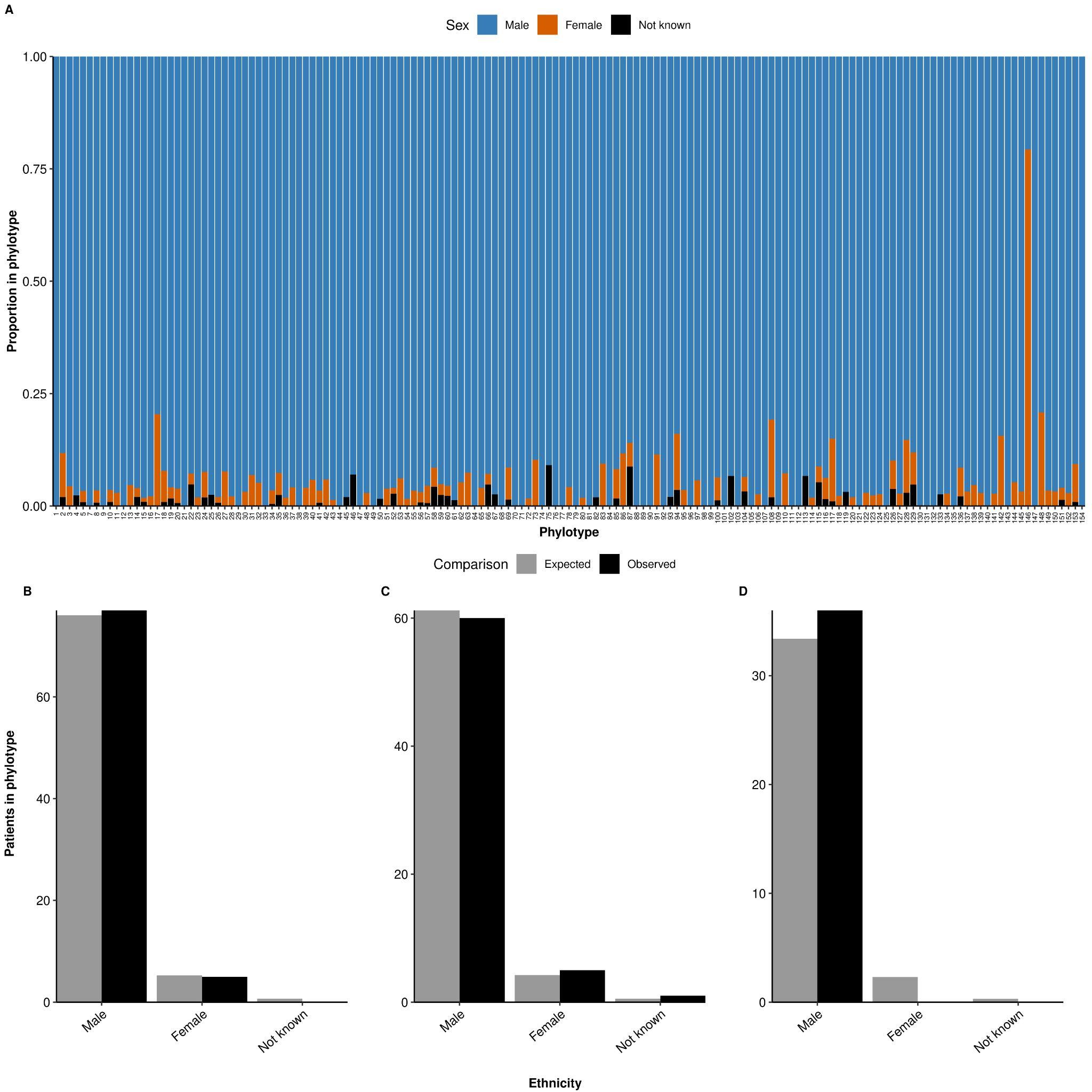


### Figure S14. Distribution of sex at birth for individuals infected by each of the 154 subtype B phylotypes (n=24,100) and across the three VOIs. (A) Percentage of people per sex at birth within each phylotype. (B) Expected under null *vs* observed distributions of sex at birth categories given by Chi-squared test for phylotypes (B) PT.B.40.UK, (C) PT.B.69.UK, and (D) PT.B.133.UK.


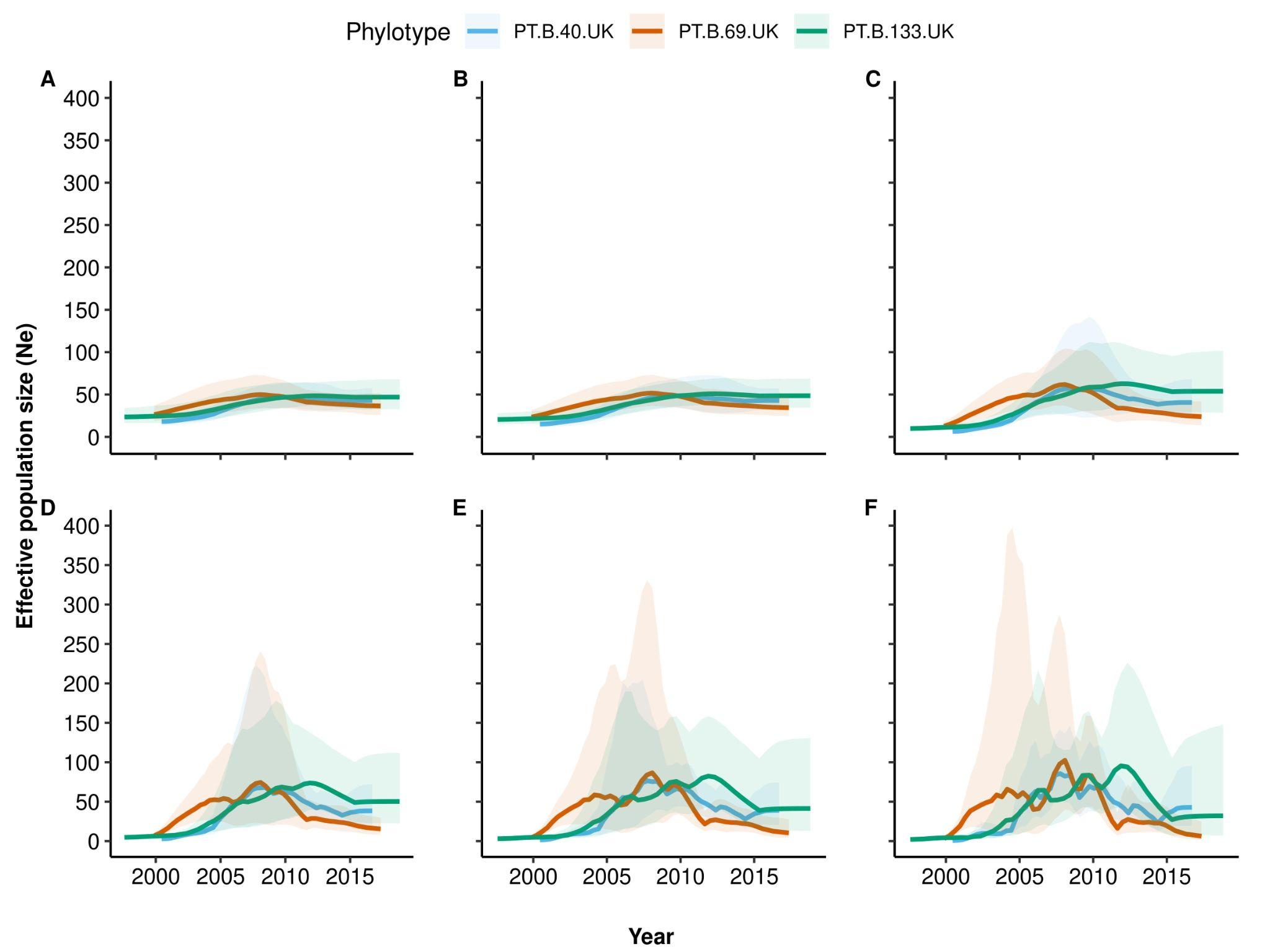


### Figure S15. Ne(t) for the HIV-1 subtype B VOIs calculated using 50 grid points, the skygrid demographic model, and variations of the precision/smoothness (τ) parameter. (A-F) τ={100, 75, 25, 10, 5, 2.5}. τ=50 is shown in Figure 3A. Shaded regions indicated 95% CIs estimated using parametric bootstrap.


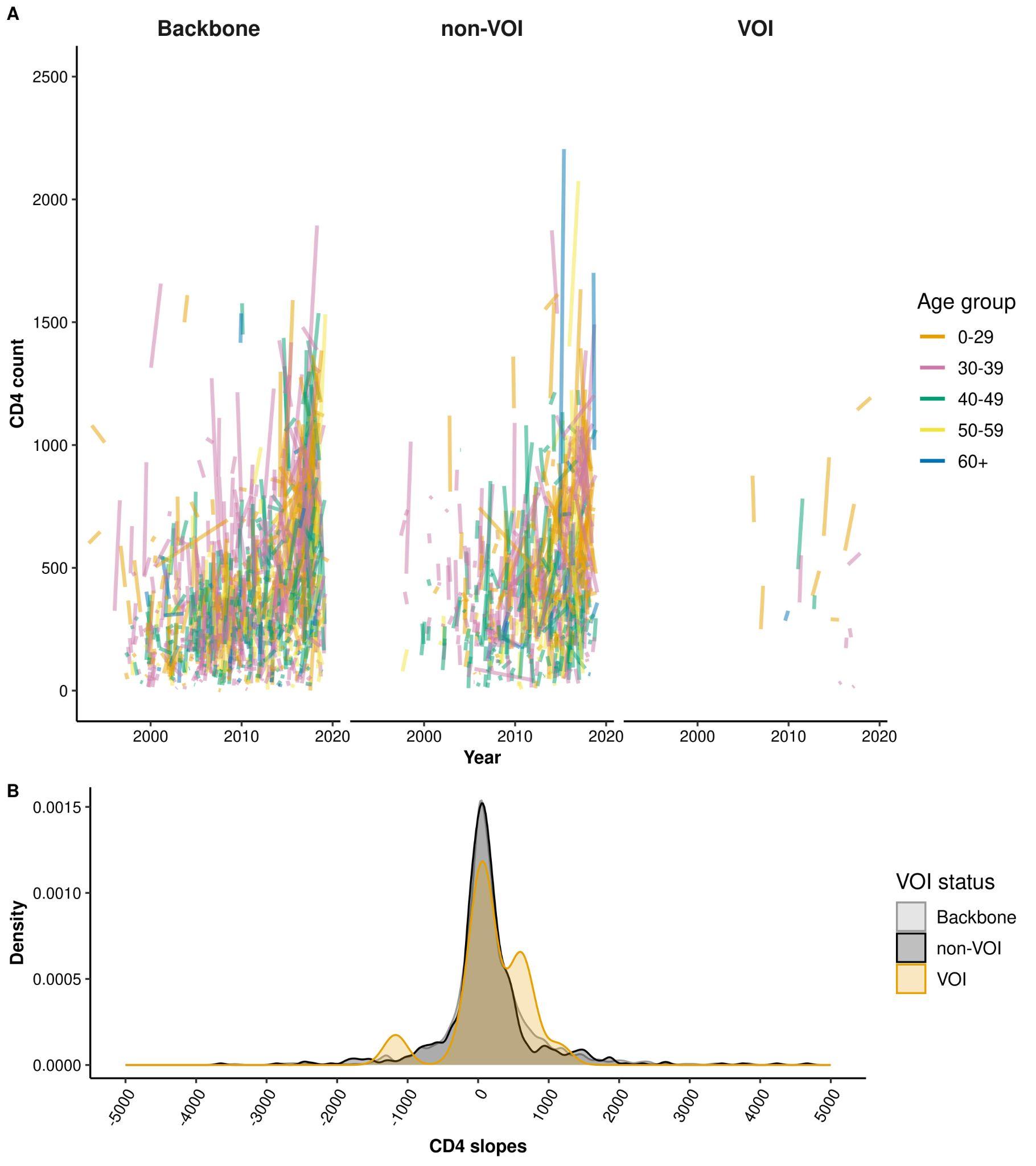


### Figure S16. Exploratory analysis of post-treatment CD4 measurements according to VOI status (VOI, non-VOI, and backbone) for subtype B. (A) Individual slopes per VOI status coloured by age group. (B) Overlaid distributions of CD4 slopes per VOI status.

###


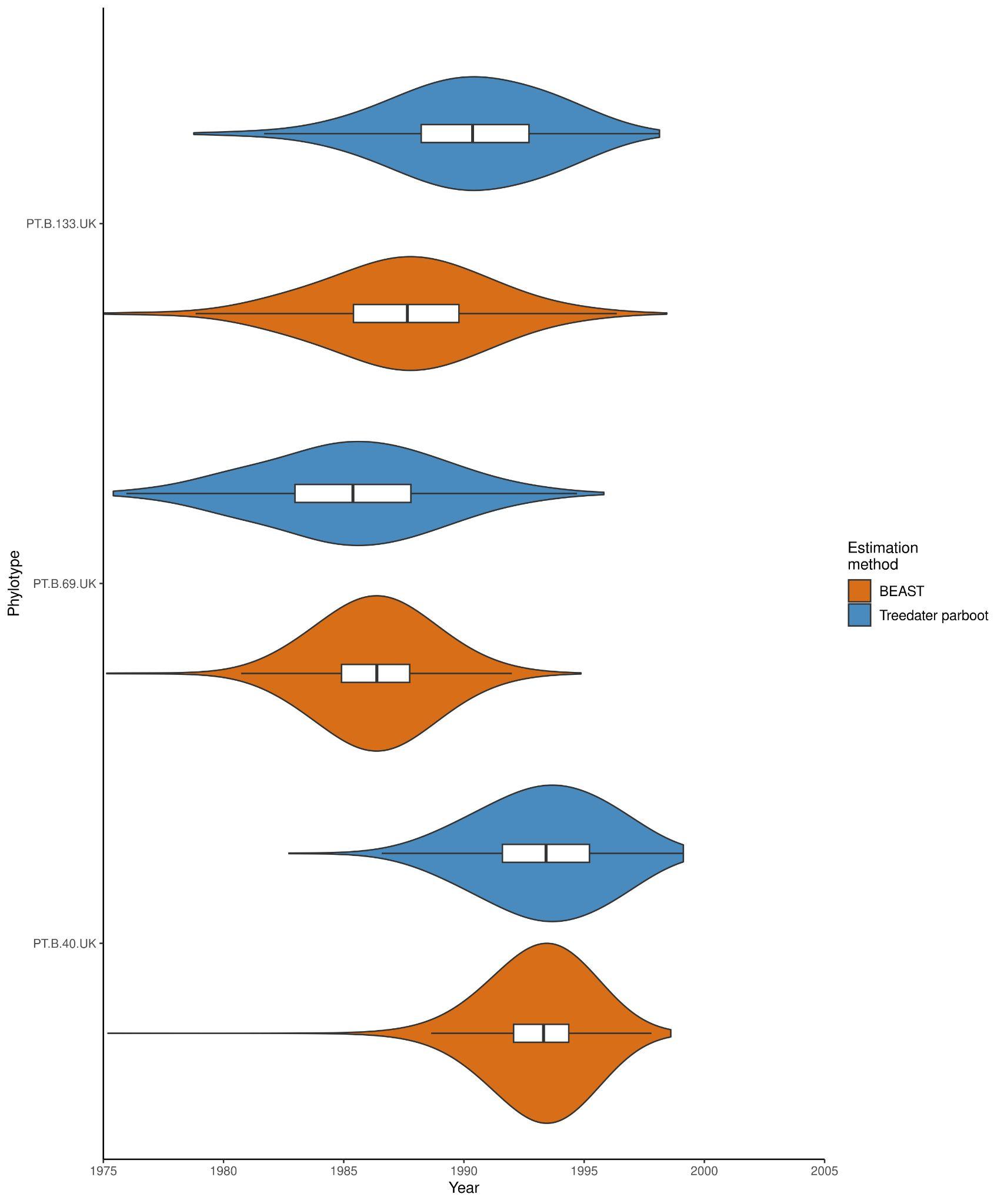


### Figure S17. Distribution of TMRCA estimates of the three identified VOIs (PT.B.40.UK, PT.B.69.UK, and PT.B.133.UK) alongside 95% identity global matches for 10000 trees resulting from both Bayesian (BEAST) and ML (treedater parametric bootstrap) estimations. The central line of the boxplot represents the median; the left and right edges of the box represent the first (25%) and third (75%) quartiles; and the whiskers (straight lines) extend from the ends of the box to the minimum and maximum values, excluding outliers.


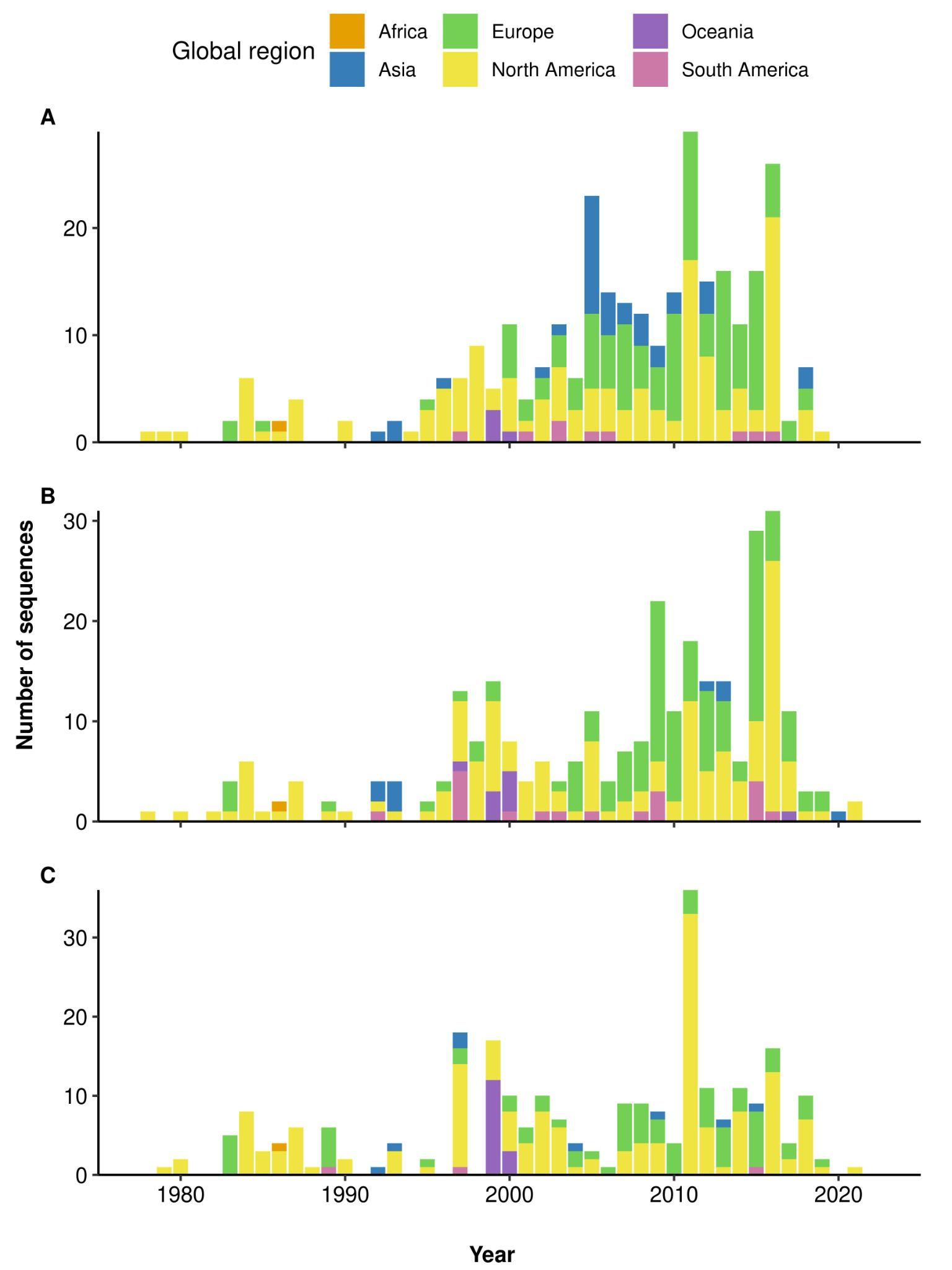


### Figure S18. Global distribution over time of the VOI phylotypes (A) PT.B.40.UK, (B) PT.B.69.UK, and (C) PT.B.133.UK combined with their respective 250 random BLAST global matches at an identity threshold of 95%. This figure is related to the tree presented in Fig. 4A and C.


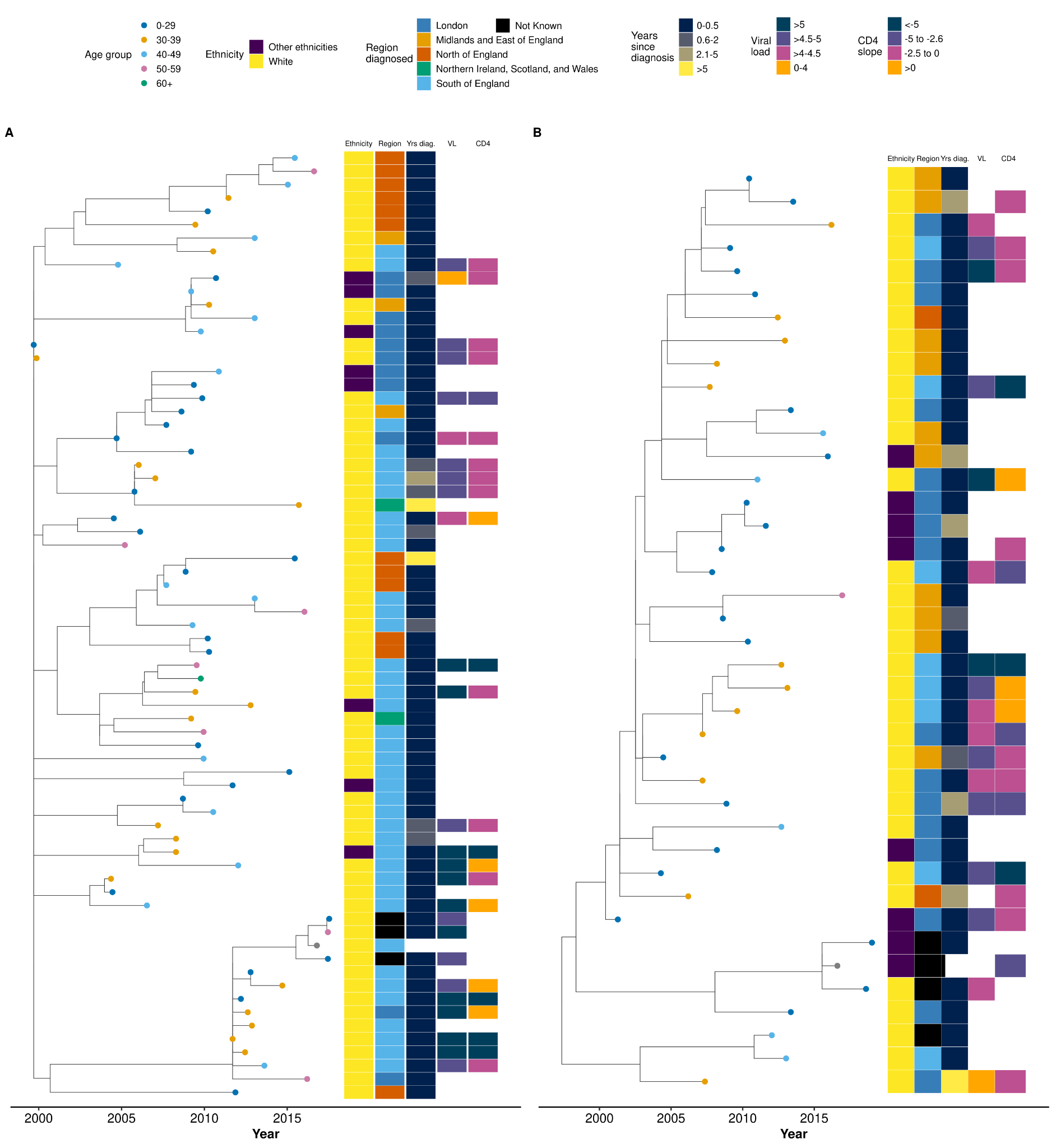


### Figure S19. Time-scaled tree of the pol gene for the main VOI phylotypes (PT.B.69.UK and PT.B.133.UK) similar to Figure 4A and C but also including ethnicity and years since diagnosis annotations. Points are coloured by age group at diagnosis and side panel variables are labelled.

###


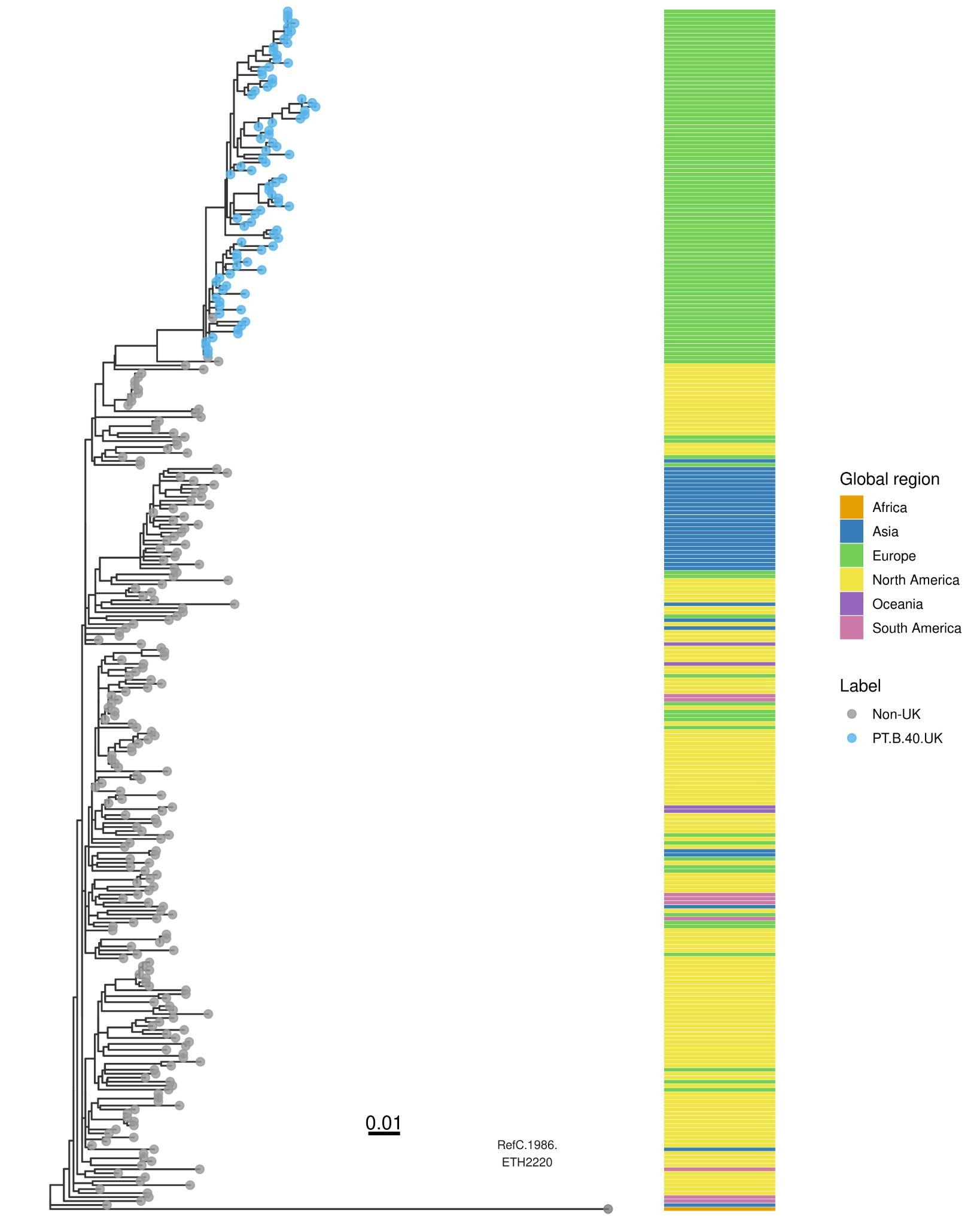


### Figure S20. Maximum likelihood tree of VOI PT.B.40.UK combined with 250 random BLAST global matches at an identity threshold of 95%. The side panel depicts global regions of sampling.

###


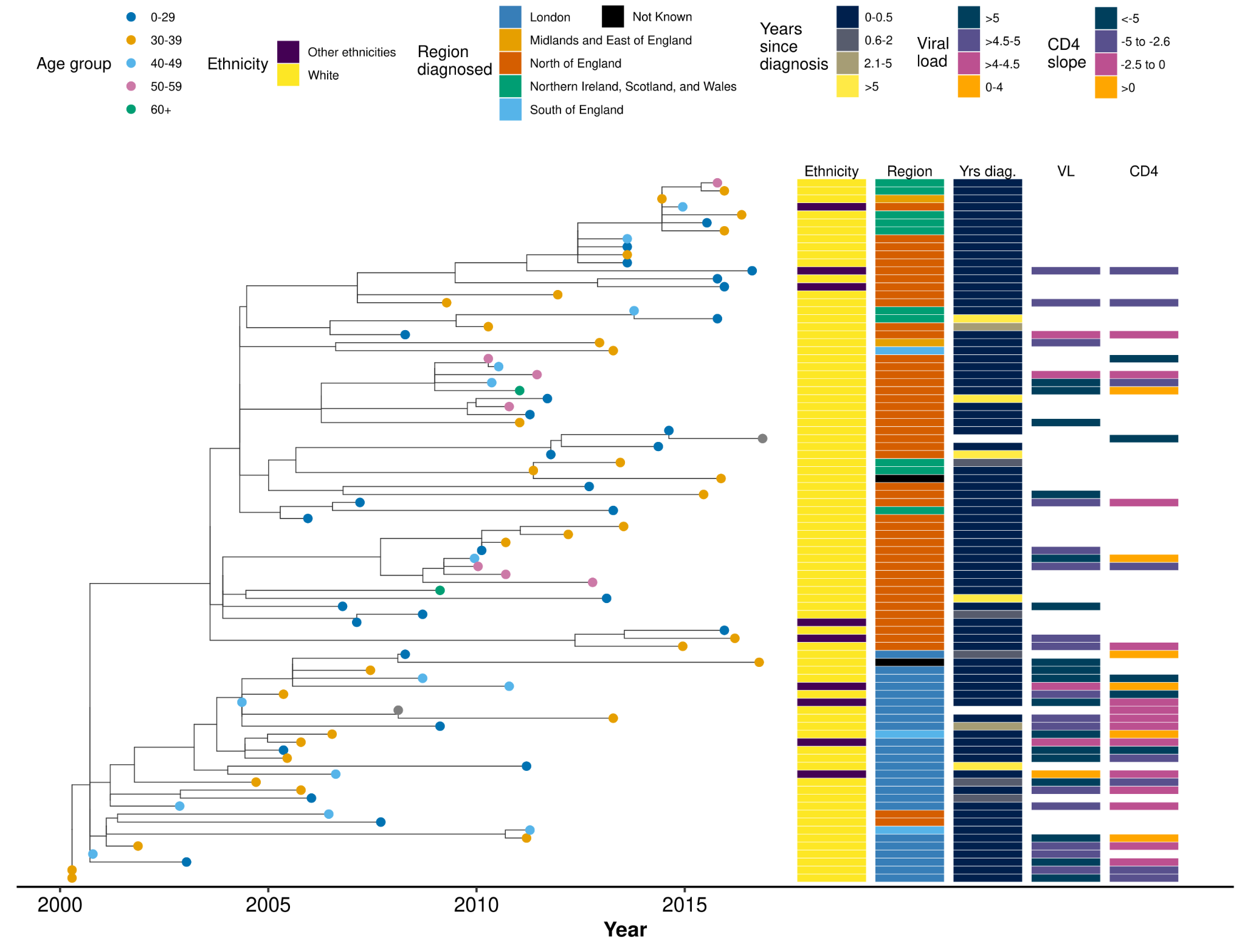


### Figure S21. Time-scaled tree of phylotypes from partial pol gene of VOI PT.B.40.UK. Tip points are coloured by age group at diagnosis and side panels represent ethnicity, region of diagnosis, years since diagnosis, mean viral load intervals (in log_10_ copies/mL), and CD4 slope ranges (in cells/mm^3^/year) per individual.

## **Supplementary tables**

### Table S1. Demographic characteristics of all individuals from the UKRDB database for each of the more represented subtypes (B, C, A1, and CRF02_AG) and all others combined. Percentages refer to different categories within each subtype (columns).

|  |  | **HIV-1 subtypes** | | | | |  |
| --- | --- | --- | --- | --- | --- | --- | --- |
|  |  | **B** | **C** | **A1** | **CRF02_AG** | **Others** | **Total** |
| **Sex** | Male | 34,486 (76.8%) | 6999 (30.3) | 2384 (37.7) | 1969 (38.1) | 4806 (47.6) | 50,644 (56.5) |
|  | Female | 2727 (6.1) | 10,905 (47.2) | 2395 (37.9) | 2215 (42.8) | 2438 (24.1) | 20,680 (23.1) |
|  | Other/NA | 7686 (17.1) | 5215 (22.6) | 1543 (24.4) | 987 (19.1) | 2856 (28.3) | 18,287 (20.4) |
| **Ethnicity** | White | 31061 (69.2) | 2664 (10.0) | 1618 (25.6) | 817 (15.8) | 3593 (35.6) | 39753 (44.4) |
|  | Black-Caribbean/African/other | 2975 (6.6) | 14248 (62.7) | 2858 (45.2) | 3101 (60.0) | 2564 (25.4) | 25746 (28.7) |
|  | Other Asian/Oriental | 834 (1.9) | 261 (1.1) | 82 (1.3) | 50 (1.0) | 466 (4.6) | 1693 (1.9) |
|  | Indian/Pakistani/Bangladeshi | 425 (0.9) | 309 (1.4) | 50 (0.8) | 21 (0.4) | 106 (1.0) | 911 (1.0) |
|  | Other/mixed/NA | 9605 (21.4) | 5637 (24.8) | 1714 (27.1) | 1182 (22.9) | 3371 (33.4) | 21509 (24.0) |
| **Risk group** | Homo/bisexual | 30516 (68.0) | 999 (4.3) | 847 (13.4) | 645 (12.5) | 2562 (25.4) | 35569 (39.7) |
|  | Heterosexual | 5127 (11.4) | 16543 (71.6) | 3580 (56.6) | 3393 (65.6) | 4304 (42.6) | 32947 (36.8) |
|  | IDU and blood products | 1366 (3.0) | 335 (1.4) | 293 (4.6) | 65 (1.3) | 256 (2.5) | 2315 (2.6) |
|  | Other/NA | 7891 (17.6) | 5242 (22.7) | 1602 (25.3) | 1068 (20.6) | 2978 (29.5) | 18781 (21.0) |
| **Region of diagnosis** | London | 18890 (42.1) | 6062 (26.2) | 2746 (43.4) | 2667 (51.6) | 3669 (36.3) | 34034 (38.0) |
|  | North of England | 5863 (13.1) | 3556 (15.4) | 639 (10.1) | 450 (8.7) | 894 (8.8) | 11402 (12.7) |
|  | Midlands and East of England | 4427 (9.9) | 4708 (20.4) | 641 (10.1) | 541 (10.5) | 980 (9.7) | 11297 (12.6) |
|  | South of England | 4735 (10.5) | 2491 (10.8) | 409 (6.5) | 275 (5.3) | 885 (8.8) | 8795 (9.8) |
|  | Northern Ireland, Scotland, and Wales | 2647 (5.9) | 955 (4.1) | 231 (3.6) | 131 (2.5) | 498 (4.9) | 4462 (5.0) |
|  | Other/NA | 8338 (10.5) | 5347 (23.1) | 1656 (26.2) | 1107 (21.4) | 3174 (31.4) | 19622 (21.9) |
| **Age at diagnosis** | <29 | 12039 (26.8) | 4833 (20.9) | 1318 (20.8) | 1172 (22.7) | 2166 (21.4) | 21528 (24.0) |
|  | 30-39 | 13511 (30.1) | 7233 (31.3) | 1857 (29.4) | 1573 (30.4) | 2660 (26.3) | 26834 (29.9) |
|  | 40-49 | 7590 (16.9) | 3791 (16.4) | 993 (15.7) | 871 (16.8) | 1430 (14.6) | 14675 (16.4) |
|  | 50-59 | 2719 (6.1) | 1364 (5.9) | 369 (5.8) | 366 (7.1) | 629 (6.2) | 5447 (6.1) |
|  | >60 | 938 (2.1) | 441 (1.9) | 140 (2.2) | 156 (3.0) | 310 (3.1) | 1985 (2.2) |
|  | NA | 8103 (18.0) | 5457 (23.6) | 1645 (26.0) | 1033 (20.0) | 2905 (28.8) | 19,143 (21.4) |

### Table S2. Demographic characteristics of all individuals from the UKRDB database for each of the more represented subtypes (B, C, A1, and CRF02_AG) and all others combined after performing (i) sequence selection considering quality, pol minimum length, only for individuals while treatment naive, and non-molecular-clock outliers and (ii) CD4 data selection for individuals with matched sequences with at least two measurements before treatment initiation and passing additional CD4 filtering for outlying measurements and individuals.

|  |  |  | **HIV-1 subtypes** | | | |  |
| --- | --- | --- | --- | --- | --- | --- | --- |
|  |  |  | **B** | **C** | **A1** | **CRF02_AG** | **Total** |
| **After sequence quality, treatment naive, and molecular clock filters** | **Sex** | Male | 22,341 (92.7) | 4453 (39.3) | 1442 (53.1) | 1345 (49.0) | 29,581 (72.4) |
|  |  | Female | 1551 (6.4) | 6742 (59.5) | 1255 (46.2) | 1380 (50.3) | 10,928 (26.7) |
|  |  | Other/NA | 208 (0.9) | 136 (1.2) | 17 (0.6) | 18 (0.7) | 379 (0.9) |
|  | **Ethnicity** | White | 19,804 (82.2) | 1795 (15.8) | 1103 (40.6) | 564 (20.6) | 23,266 (56.9) |
|  |  | Black-Caribbean/African/other | 1872 (7.8) | 8697 (76.8) | 1407 (51.8) | 1983 (72.3) | 13,959 (34.1) |
|  |  | Other Asian/Oriental | 610 (2.5) | 166 (1.5) | 38 (1.4) | 28 (1) | 842 (2.1) |
|  |  | Indian/Pakistani/Bangladeshi | 293 (1.2) | 214 (1.9) | 34 (1.3) | 15 (0.5) | 556 (1.4) |
|  |  | Other/mixed/NA | 1521 (6.3) | 459 (4.1) | 132 (4.9) | 153 (5.6) | 2265 (5.5) |
|  | **Risk group** | Homo/bisexual | 20,215 (83.9) | 704 (6.2) | 592 (21.8) | 467 (17.0) | 21,978 (53.8) |
|  |  | Heterosexual | 3285 (13.6) | 10,402 (91.8) | 1926 (71.0) | 2227 (81.2) | 17,840 (43.6) |
|  |  | IDU and blood products | 587 (2.4) | 207 (1.8) | 189 (7.0) | 41 (1.5) | 1024 (2.5) |
|  | **Region of diagnosis** | London | 11,535 (47.9) | 3451 (30.5) | 1367 (50.4) | 1696 (61.8) | 18,049 (44.2) |
|  |  | North of England | 3837 (15.9) | 2249 (19.8) | 428 (15.8) | 303 (11.0) | 6817 (16.7) |
|  |  | Midlands and East of England | 3151 (13.1) | 3118 (27.5) | 421 (15.5) | 385 (14.0) | 7075 (17.3) |
|  |  | South of England | 3376 (14) | 1718 (15.2) | 268 (9.9) | 191 (7.0) | 5553 (13.6) |
|  |  | Northern Ireland, Scotland, and Wales | 1661 (6.9) | 614 (5.4) | 153 (5.6) | 84 (3.1) | 2512 (6.2) |
|  |  | Other/NA | 540 (2.2) | 181 (1.6) | 77 (2.8) | 84 (3.1) | 882 (2.2) |
|  | **Age at diagnosis** | <29 | 7284 (30.2) | 2760 (24.4) | 719 (26.5) | 699 (25.5) | 11,462 (28.0) |
|  |  | 30-39 | 8538 (35.4) | 4468 (39.4) | 987 (36.4) | 987 (36) | 14980 (36.7) |
|  |  | 40-49 | 5281 (21.9) | 2568 (22.7) | 624 (23.0) | 618 (22.5) | 9091 (22.2) |
|  |  | 50-59 | 1978 (8.2) | 977 (8.6) | 226 (8.3) | 269 (9.8) | 3450 (8.4) |
|  |  | >60 | 691 (2.9) | 306 (2.7) | 97 (3.6) | 118 (4.3) | 1212 (3.0) |
|  |  | NA | 328 (1.4) | 252 (2.2) | 61 (2.2) | 52 (1.9) | 693 (1.7) |
| **CD4 analysis** | **Sex** | Male | 8537 (95.4) | 856 (40.8) | 355 (50.7) | 321 (51.9) | 10,069 (81.5) |
|  |  | Female | 409 (4.6) | 1241 (59.2) | 345 (49.3) | 298 (48.1) | 2293 (18.5) |
|  | **Ethnicity** | White | 7353 (82.2) | 349 (16.6) | 258 (36.9) | 150 (24.2) | 8110 (65.6) |
|  |  | Black-Caribbean/African/other | 643 (7.2) | 1563 (74.5) | 397 (56.7) | 430 (69.5) | 3033 (24.5) |
|  |  | Other Asian/Oriental | 207 (2.3) | 19 (0.9) | 4 (0.6) | 5 (0.8) | 235 (1.9) |
|  |  | Indian/Pakistani/Bangladeshi | 126 (1.4) | 46 (2.2) | 15 (2.1) | 3 (0.5) | 190 (1.5) |
|  |  | Other/mixed/NA | 617 (6.9) | 121 (5.8) | 31 (5.0) | 26 (3.7) | 795 (6.4) |
|  | **Risk group** | Homo/bisexual | 8033 (89.8) | 201 (9.6) | 165 (23.6) | 146 (23.6) | 8545 (69.2) |
|  |  | Heterosexual | 713 (8.0) | 1852 (88.3) | 494 (70.6) | 458 (74.0) | 3517 (28.5) |
|  |  | IDU and blood products | 197 (2.2) | 35 (1.7) | 36 (5.1) | 11 (1.8) | 279 (2.3) |
|  | **Region of diagnosis** | London | 6644 (74.3) | 1150 (54.8) | 499 (71.3) | 490 (79.2) | 8783 (71.0) |
|  |  | North of England | 444 (5.0) | 157 (7.5) | 49 (7.0) | 32 (5.2) | 682 (5.5) |
|  |  | Midlands and East of England | 401 (4.5) | 404 (19.3) | 66 (9.4) | 48 (7.8) | 919 (7.4) |
|  |  | South of England | 1229 (13.7) | 313 (14.9) | 61 (8.7) | 30 (4.8) | 1633 (13.2) |
|  |  | Northern Ireland, Scotland, and Wales | 143 (1.6) | 42 (2.0) | 12 (1.7) | 3 (0.5) | 200 (1.6) |
|  |  | Other/NA | 85 (1.0) | 32 (1.5) | 13 (1.9) | 16 (2.6) | 146 (1.2) |
|  | **Age at diagnosis** | <29 | 2883 (32.2) | 620 (29.6) | 223 (31.9) | 188 (30.4) | 3914 (31.7) |
|  |  | 30-39 | 3521 (39.4) | 811 (38.7) | 256 (36.6) | 221 (35.7) | 4809 (38.9) |
|  |  | 40-49 | 1804 (20.2) | 427 (20.4) | 141 (20.1) | 126 (20.4) | 2498 (20.2) |
|  |  | 50-59 | 484 (5.4) | 134 (6.4) | 46 (6.6) | 45 (7.3) | 709 (5.7) |
|  |  | >60 | 126 (1.4) | 49 (2.3) | 14 (2.0) | 21 (3.4) | 210 (1.7) |
|  |  | NA | 128 (1.4) | 57 (2.7) | 20 (2.9) | 18 (2.9) | 223 (1.8) |

### Table S3. Individual viral load and CD4 measurements over time in the absence of treatment for the defined VOIs. The table is ordered by increasing phylotype ID and measurement date. In cases where both viral load and CD4 measurements are aligned in time (same month), they are displayed in the same row, otherwise only the one measure taken at that time point is shown. Sequence IDs are not necessarily in order because measurement date ordering precedes it.

| **Subtype** | **Phylotype**  **ID** | **Sequence**  **ID** | **Antiretroviral**  **therapy start date**  **(month/year)** | **Measurement**  **date**  **(month/year)** | **Viral load**  **(log_10_**  **copies/mL)** | **CD4 count**  **(cells/mm^3^)** |
| --- | --- | --- | --- | --- | --- | --- |
| B | 40 | t.89638 | 10/2000 | 03/2000 | 5.699 | 230 |
| B | 40 | t.130439 | 03/2003 | 04/2000 | 5.122 | 780 |
| B | 40 | t.89638 | 10/2000 | 04/2000 | 6.017 | 270 |
| B | 40 | t.130439 | 03/2003 | 05/2000 | 4.371 | 560 |
| B | 40 | t.89638 | 10/2000 | 05/2000 | 5.645 | 230 |
| B | 40 | t.130439 | 03/2003 | 06/2000 | 4.449 |  |
| B | 40 | t.89638 | 10/2000 | 06/2000 | 5.284 |  |
| B | 40 | t.130439 | 03/2003 | 07/2000 | 3.863 |  |
| B | 40 | t.89638 | 10/2000 | 07/2000 | 5.257 |  |
| B | 40 | t.130439 | 03/2003 | 10/2000 | 4.49 |  |
| B | 40 | t.79063 | 10/2000 | 10/2000 | 4.91 |  |
| B | 40 | t.89638 | 10/2000 | 10/2000 | 5.137 | 170 |
| B | 40 | t.130439 | 03/2003 | 01/2001 | 4.486 |  |
| B | 40 | t.130439 | 03/2003 | 03/2001 | 4.588 |  |
| B | 40 | t.130439 | 03/2003 | 07/2001 | 4.511 |  |
| B | 40 | t.130439 | 03/2003 | 08/2001 | 4.754 |  |
| B | 40 | t.130439 | 03/2003 | 09/2001 | 4.886 |  |
| B | 40 | t.120998 | 02/2008 | 11/2001 | 5.4 | 368 |
| B | 40 | t.130439 | 03/2003 | 11/2001 | 5.155 |  |
| B | 40 | t.130439 | 03/2003 | 12/2001 | 5.414 |  |
| B | 40 | t.120998 | 02/2008 | 01/2002 | 5.019 | 406 |
| B | 40 | t.130439 | 03/2003 | 02/2002 | 5.167 |  |
| B | 40 | t.130439 | 03/2003 | 04/2002 | 5.166 |  |
| B | 40 | t.120998 | 02/2008 | 05/2002 | 4.749 | 329 |
| B | 40 | t.120998 | 02/2008 | 08/2002 | 4.445 |  |
| B | 40 | t.123094 | 03/2006 | 10/2002 | 5.46 | 320 |
| B | 40 | t.123094 | 03/2006 | 11/2002 | 4.934 | 540 |
| B | 40 | t.120998 | 02/2008 | 01/2003 | 4.818 |  |
| B | 40 | t.56741 | 02/2011 | 01/2003 | 5.452 |  |
| B | 40 | t.123094 | 03/2006 | 02/2003 | 4.721 | 640 |
| B | 40 | t.56741 | 02/2011 | 02/2003 | 5.446 | 521 |
| B | 40 | t.120998 | 02/2008 | 03/2003 | 4.786 |  |
| B | 40 | t.130439 | 03/2003 | 03/2003 |  | 190 |
| B | 40 | t.123094 | 03/2006 | 04/2003 | 4.808 |  |
| B | 40 | t.56741 | 02/2011 | 04/2003 | 5.569 | 477 |
| B | 40 | t.120998 | 02/2008 | 07/2003 | 4.928 |  |
| B | 40 | t.56741 | 02/2011 | 07/2003 | 5.138 | 483 |
| B | 40 | t.123094 | 03/2006 | 09/2003 | 4.401 |  |
| B | 40 | t.56741 | 02/2011 | 11/2003 | 5.176 |  |
| B | 40 | t.10889 | 02/2009 | 12/2003 | 5.538 | 430 |
| B | 40 | t.140449 | 03/2008 | 12/2003 |  | 460 |
| B | 40 | t.10889 | 02/2009 | 01/2004 | 4.981 | 330 |
| B | 40 | t.10889 | 02/2009 | 03/2004 | 3.845 | 350 |
| B | 40 | t.2729 | 09/2004 | 03/2004 |  | 580 |
| B | 40 | t.123094 | 03/2006 | 04/2004 | 4.843 |  |
| B | 40 | t.10889 | 02/2009 | 05/2004 | 5.019 |  |
| B | 40 | t.2729 | 09/2004 | 05/2004 | 5.544 | 540 |
| B | 40 | t.56741 | 02/2011 | 05/2004 | 4.813 |  |
| B | 40 | t.140449 | 03/2008 | 06/2004 |  | 440 |
| B | 40 | t.2729 | 09/2004 | 06/2004 | 5.352 | 420 |
| B | 40 | t.2729 | 09/2004 | 09/2004 | 5.24 | 570 |
| B | 40 | t.43732 | 06/2005 | 09/2004 | 5.097 | 284 |
| B | 40 | t.43732 | 06/2005 | 10/2004 |  | 361 |
| B | 40 | t.56741 | 02/2011 | 11/2004 | 4.946 |  |
| B | 40 | t.43732 | 06/2005 | 12/2004 |  | 345 |
| B | 40 | t.10889 | 02/2009 | 02/2005 | 4.554 |  |
| B | 40 | t.140449 | 03/2008 | 02/2005 |  | 490 |
| B | 40 | t.42344 | 10/2005 | 04/2005 | 5.21 | 340 |
| B | 40 | t.42344 | 10/2005 | 05/2005 | 4.698 |  |
| B | 40 | t.43732 | 06/2005 | 05/2005 |  | 220 |
| B | 40 | t.64224 | 05/2006 | 05/2005 |  | 288 |
| B | 40 | t.52864 | 12/2005 | 06/2005 |  | 282 |
| B | 40 | t.64224 | 05/2006 | 06/2005 | 5.299 | 341 |
| B | 40 | t.42344 | 10/2005 | 07/2005 | 5.445 | 270 |
| B | 40 | t.42344 | 10/2005 | 09/2005 | 5.699 | 140 |
| B | 40 | t.52864 | 12/2005 | 09/2005 | 4.613 | 231 |
| B | 40 | t.64224 | 05/2006 | 09/2005 | 4.886 | 286 |
| B | 40 | t.110159 | 11/2008 | 10/2005 | 5.101 | 500 |
| B | 40 | t.52864 | 12/2005 | 10/2005 | 4.571 | 201 |
| B | 40 | t.82620 | 10/2008 | 10/2005 |  | 415 |
| B | 40 | t.110159 | 11/2008 | 12/2005 | 4.614 | 540 |
| B | 40 | t.64224 | 05/2006 | 12/2005 | 5.121 |  |
| B | 40 | t.82620 | 10/2008 | 01/2006 | 4.376 | 914 |
| B | 40 | t.110159 | 11/2008 | 02/2006 | 4.691 | 420 |
| B | 40 | t.123094 | 03/2006 | 03/2006 |  | 210 |
| B | 40 | t.64224 | 05/2006 | 03/2006 | 5.09 | 192 |
| B | 40 | t.82620 | 10/2008 | 04/2006 | 4.449 | 529 |
| B | 40 | t.82620 | 10/2008 | 05/2006 |  | 671 |
| B | 40 | t.110159 | 11/2008 | 06/2006 | 4.852 |  |
| B | 40 | t.86292 | 08/2006 | 07/2006 | 5.178 | 195 |
| B | 40 | t.19766 | 08/2012 | 08/2006 | 3.967 | 320 |
| B | 40 | t.86292 | 08/2006 | 08/2006 | 4.965 | 225 |
| B | 40 | t.110159 | 11/2008 | 09/2006 | 5.126 |  |
| B | 40 | t.19766 | 08/2012 | 09/2006 |  | 320 |
| B | 40 | t.82620 | 10/2008 | 09/2006 | 4.41 |  |
| B | 40 | t.103053 | 06/2013 | 10/2006 | 4.974 |  |
| B | 40 | t.134052 | 11/2006 | 10/2006 | 5.546 |  |
| B | 40 | t.103053 | 06/2013 | 11/2006 |  | 743 |
| B | 40 | t.19766 | 08/2012 | 11/2006 | 3.492 | 390 |
| B | 40 | t.82620 | 10/2008 | 11/2006 | 4.581 |  |
| B | 40 | t.110159 | 11/2008 | 12/2006 | 4.656 |  |
| B | 40 | t.82620 | 10/2008 | 02/2007 | 4.627 |  |
| B | 40 | t.103053 | 06/2013 | 03/2007 | 4.881 | 529 |
| B | 40 | t.19766 | 08/2012 | 03/2007 | 3.453 |  |
| B | 40 | t.110159 | 11/2008 | 04/2007 | 4.447 |  |
| B | 40 | t.82620 | 10/2008 | 05/2007 | 4.169 |  |
| B | 40 | t.28358 | 06/2007 | 06/2007 | 6.773 |  |
| B | 40 | t.103053 | 06/2013 | 07/2007 |  | 555 |
| B | 40 | t.110159 | 11/2008 | 07/2007 | 4.939 |  |
| B | 40 | t.19766 | 08/2012 | 07/2007 | 3.365 |  |
| B | 40 | t.82620 | 10/2008 | 08/2007 | 4.391 |  |
| B | 40 | t.82620 | 10/2008 | 09/2007 | 4.511 |  |
| B | 40 | t.110159 | 11/2008 | 10/2007 | 4.76 |  |
| B | 40 | t.19766 | 08/2012 | 11/2007 | 2.782 |  |
| B | 40 | t.120998 | 02/2008 | 01/2008 |  | 175 |
| B | 40 | t.140449 | 03/2008 | 01/2008 |  | 170 |
| B | 40 | t.78669 | 06/2008 | 03/2008 |  | 140 |
| B | 40 | t.103053 | 06/2013 | 04/2008 | 4.786 |  |
| B | 40 | t.19766 | 08/2012 | 04/2008 | 3.136 |  |
| B | 40 | t.78669 | 06/2008 | 04/2008 |  | 290 |
| B | 40 | t.97781 | 11/2011 | 04/2008 | 4.484 | 242 |
| B | 40 | t.78669 | 06/2008 | 05/2008 |  | 140 |
| B | 40 | t.97781 | 11/2011 | 06/2008 |  | 240 |
| B | 40 | t.19766 | 08/2012 | 07/2008 | 3.106 |  |
| B | 40 | t.103053 | 06/2013 | 09/2008 | 4.504 |  |
| B | 40 | t.74135 | 10/2008 | 09/2008 | 5.223 | 827 |
| B | 40 | t.82620 | 10/2008 | 09/2008 |  | 363 |
| B | 40 | t.97781 | 11/2011 | 09/2008 |  | 211 |
| B | 40 | t.110159 | 11/2008 | 10/2008 |  | 260 |
| B | 40 | t.74135 | 10/2008 | 10/2008 |  | 452 |
| B | 40 | t.10889 | 02/2009 | 01/2009 |  | 290 |
| B | 40 | t.103053 | 06/2013 | 03/2009 |  | 411 |
| B | 40 | t.9288 | 06/2011 | 04/2009 | 4.583 | 608 |
| B | 40 | t.9288 | 06/2011 | 09/2009 |  | 588 |
| B | 40 | t.76078 | 10/2010 | 12/2009 | 5.544 | 359 |
| B | 40 | t.3698 | 11/2010 | 01/2010 | 5.19 | 444 |
| B | 40 | t.76078 | 10/2010 | 01/2010 |  | 504 |
| B | 40 | t.9288 | 06/2011 | 01/2010 | 5.114 | 644 |
| B | 40 | t.3698 | 11/2010 | 02/2010 | 4.613 | 427 |
| B | 40 | t.76078 | 10/2010 | 03/2010 | 5.681 |  |
| B | 40 | t.133815 | 07/2010 | 04/2010 |  | 166 |
| B | 40 | t.133815 | 07/2010 | 05/2010 | 6.605 | 166 |
| B | 40 | t.76078 | 10/2010 | 05/2010 | 5.778 | 400 |
| B | 40 | t.9288 | 06/2011 | 05/2010 | 4.703 | 427 |
| B | 40 | t.3698 | 11/2010 | 06/2010 | 4.633 |  |
| B | 40 | t.133815 | 07/2010 | 07/2010 | 6.359 | 148 |
| B | 40 | t.3698 | 11/2010 | 07/2010 |  | 307 |
| B | 40 | t.76078 | 10/2010 | 08/2010 |  | 430 |
| B | 40 | t.115048 | 11/2010 | 09/2010 | 4.146 | 220 |
| B | 40 | t.115048 | 11/2010 | 10/2010 | 4.255 | 240 |
| B | 40 | t.3698 | 11/2010 | 10/2010 |  | 353 |
| B | 40 | t.15152 | 12/2011 | 11/2010 | 4.949 |  |
| B | 40 | t.15152 | 12/2011 | 12/2010 | 4.929 |  |
| B | 40 | t.120970 | 08/2014 | 01/2011 |  | 709 |
| B | 40 | t.56741 | 02/2011 | 01/2011 |  | 303 |
| B | 40 | t.59537 | 02/2011 | 01/2011 | 5.049 |  |
| B | 40 | t.60254 | 02/2011 | 01/2011 | 6.413 | 53 |
| B | 40 | t.15152 | 12/2011 | 02/2011 | 5.38 |  |
| B | 40 | t.60254 | 02/2011 | 02/2011 |  | 250 |
| B | 40 | t.15152 | 12/2011 | 03/2011 | 5.204 |  |
| B | 40 | t.40179 | 05/2011 | 03/2011 | 5.72 | 247 |
| B | 40 | t.15152 | 12/2011 | 04/2011 | 5.204 |  |
| B | 40 | t.40179 | 05/2011 | 04/2011 |  | 351 |
| B | 40 | t.15152 | 12/2011 | 06/2011 | 2.851 |  |
| B | 40 | t.97781 | 11/2011 | 11/2011 |  | 176 |
| B | 40 | t.120970 | 08/2014 | 06/2012 |  | 484 |
| B | 40 | t.19766 | 08/2012 | 06/2012 |  | 340 |
| B | 40 | t.120970 | 08/2014 | 10/2012 | 4.491 |  |
| B | 40 | t.140498 | 04/2013 | 12/2012 | 4.908 |  |
| B | 40 | t.140498 | 04/2013 | 02/2013 | 5.083 |  |
| B | 40 | t.1804 | 11/2013 | 04/2013 | 4.727 | 340 |
| B | 40 | t.120970 | 08/2014 | 06/2013 |  | 633 |
| B | 40 | t.1804 | 11/2013 | 08/2013 | 4.592 | 320 |
| B | 40 | t.120970 | 08/2014 | 09/2013 |  | 387 |
| B | 40 | t.120970 | 08/2014 | 08/2014 |  | 443 |
| B | 40 | t.133940 | 02/2017 | 11/2014 | 4.778 |  |
| B | 40 | t.133940 | 02/2017 | 12/2014 | 4.573 | 715 |
| B | 40 | t.133940 | 02/2017 | 01/2015 |  | 592 |
| B | 40 | t.133940 | 02/2017 | 02/2015 |  | 630 |
| B | 40 | t.17083 | 06/2015 | 06/2015 | 6.875 |  |
| B | 40 | t.28794 | 03/2016 | 02/2016 | 4.854 |  |
| B | 40 | t.133940 | 02/2017 | 03/2016 | 4.519 |  |
| B | 40 | t.106704 | 05/2018 | 05/2016 |  | 964 |
| B | 40 | t.106704 | 05/2018 | 08/2016 | 4.542 | 695 |
| B | 40 | t.10368 | 10/2016 | 10/2016 | 6.19 |  |
| B | 40 | t.100208 | 07/2017 | 11/2016 |  | 550 |
| B | 40 | t.133940 | 02/2017 | 11/2016 |  | 551 |
| B | 40 | t.106704 | 05/2018 | 12/2016 | 4.912 | 574 |
| B | 40 | t.106704 | 05/2018 | 03/2017 | 4.853 | 548 |
| B | 40 | t.100208 | 07/2017 | 05/2017 |  | 398 |
| B | 40 | t.106704 | 05/2018 | 09/2017 | 4.328 |  |
| B | 40 | t.106704 | 05/2018 | 05/2018 | 5.346 | 406 |
| B | 69 | t.31931 | 09/2003 | 07/1999 | 4.442 | 280 |
| B | 69 | t.31931 | 09/2003 | 08/1999 | 4.786 | 220 |
| B | 69 | t.31931 | 09/2003 | 09/1999 | 4.017 | 330 |
| B | 69 | t.31931 | 09/2003 | 10/1999 | 4.897 |  |
| B | 69 | t.31261 | 02/2002 | 11/1999 | 5.311 | 387 |
| B | 69 | t.31931 | 09/2003 | 11/1999 | 4.594 |  |
| B | 69 | t.31261 | 02/2002 | 01/2000 | 4.464 | 368 |
| B | 69 | t.31931 | 09/2003 | 01/2000 | 4.784 |  |
| B | 69 | t.31261 | 02/2002 | 03/2000 | 4.658 |  |
| B | 69 | t.31931 | 09/2003 | 04/2000 | 4.599 |  |
| B | 69 | t.31261 | 02/2002 | 06/2000 | 4.221 | 612 |
| B | 69 | t.31931 | 09/2003 | 08/2000 | 4.507 |  |
| B | 69 | t.31261 | 02/2002 | 10/2000 | 4.666 | 404 |
| B | 69 | t.31931 | 09/2003 | 11/2000 | 4.623 |  |
| B | 69 | t.31261 | 02/2002 | 01/2001 | 4.895 |  |
| B | 69 | t.31931 | 09/2003 | 02/2001 | 4.929 |  |
| B | 69 | t.31261 | 02/2002 | 04/2001 | 4.504 |  |
| B | 69 | t.31931 | 09/2003 | 04/2001 | 4.613 |  |
| B | 69 | t.31261 | 02/2002 | 07/2001 | 4.429 |  |
| B | 69 | t.31931 | 09/2003 | 07/2001 | 4.859 |  |
| B | 69 | t.31261 | 02/2002 | 10/2001 | 4.729 |  |
| B | 69 | t.31261 | 02/2002 | 01/2002 |  | 258 |
| B | 69 | t.31931 | 09/2003 | 09/2003 |  | 150 |
| B | 69 | t.20500 | 05/2007 | 01/2004 |  | 646 |
| B | 69 | t.20500 | 05/2007 | 03/2004 | 5.168 | 482 |
| B | 69 | t.135311 | 11/2010 | 05/2004 |  | 369 |
| B | 69 | t.70916 | 10/2004 | 05/2004 | 4.487 | 356 |
| B | 69 | t.20500 | 05/2007 | 06/2004 | 4.887 | 581 |
| B | 69 | t.70916 | 10/2004 | 06/2004 | 4.452 | 390 |
| B | 69 | t.70916 | 10/2004 | 07/2004 | 4.418 | 373 |
| B | 69 | t.142066 | 01/2009 | 09/2004 | 4.557 | 410 |
| B | 69 | t.20500 | 05/2007 | 09/2004 | 5.21 | 481 |
| B | 69 | t.127900 | 08/2007 | 10/2004 | 5.307 | 537 |
| B | 69 | t.142066 | 01/2009 | 10/2004 | 4.548 | 480 |
| B | 69 | t.46198 | 04/2007 | 10/2004 | 4.74 | 380 |
| B | 69 | t.127900 | 08/2007 | 11/2004 | 5.368 | 347 |
| B | 69 | t.142066 | 01/2009 | 12/2004 | 4.303 | 470 |
| B | 69 | t.127900 | 08/2007 | 01/2005 | 5.112 | 266 |
| B | 69 | t.20500 | 05/2007 | 01/2005 | 4.7 |  |
| B | 69 | t.46198 | 04/2007 | 01/2005 | 4.322 | 410 |
| B | 69 | t.127900 | 08/2007 | 03/2005 | 5.168 |  |
| B | 69 | t.125103 | 01/2010 | 04/2005 | 4.667 | 567 |
| B | 69 | t.46198 | 04/2007 | 04/2005 | 5.146 | 420 |
| B | 69 | t.127900 | 08/2007 | 05/2005 | 4.573 |  |
| B | 69 | t.20500 | 05/2007 | 05/2005 | 5.007 |  |
| B | 69 | t.73045 | 06/2007 | 05/2005 | 5.496 | 524 |
| B | 69 | t.73045 | 06/2007 | 06/2005 | 5.875 | 640 |
| B | 69 | t.127900 | 08/2007 | 07/2005 | 4.725 |  |
| B | 69 | t.46198 | 04/2007 | 07/2005 | 4.732 |  |
| B | 69 | t.73045 | 06/2007 | 07/2005 | 5.227 | 540 |
| B | 69 | t.142066 | 01/2009 | 08/2005 | 4.606 |  |
| B | 69 | t.73045 | 06/2007 | 08/2005 | 5.251 |  |
| B | 69 | t.127900 | 08/2007 | 09/2005 | 4.5 |  |
| B | 69 | t.73045 | 06/2007 | 09/2005 | 5.083 |  |
| B | 69 | t.125103 | 01/2010 | 10/2005 | 5.077 | 524 |
| B | 69 | t.20500 | 05/2007 | 10/2005 | 4.627 |  |
| B | 69 | t.46198 | 04/2007 | 10/2005 | 4.623 |  |
| B | 69 | t.73045 | 06/2007 | 10/2005 | 4.952 |  |
| B | 69 | t.127900 | 08/2007 | 12/2005 | 4.182 |  |
| B | 69 | t.46198 | 04/2007 | 01/2006 | 3.653 |  |
| B | 69 | t.73045 | 06/2007 | 01/2006 | 2.567 |  |
| B | 69 | t.135311 | 11/2010 | 02/2006 | 4.684 | 307 |
| B | 69 | t.142066 | 01/2009 | 02/2006 | 4.089 |  |
| B | 69 | t.73045 | 06/2007 | 03/2006 | 4.428 |  |
| B | 69 | t.127900 | 08/2007 | 04/2006 | 4.38 |  |
| B | 69 | t.135311 | 11/2010 | 04/2006 | 5.593 | 299 |
| B | 69 | t.73045 | 06/2007 | 04/2006 | 4.508 |  |
| B | 69 | t.135311 | 11/2010 | 05/2006 | 4.899 | 307 |
| B | 69 | t.46198 | 04/2007 | 05/2006 | 4.114 |  |
| B | 69 | t.73045 | 06/2007 | 05/2006 | 4.549 |  |
| B | 69 | t.142066 | 01/2009 | 06/2006 | 4.321 |  |
| B | 69 | t.26184 | 10/2006 | 07/2006 | 6.053 | 451 |
| B | 69 | t.73045 | 06/2007 | 07/2006 | 4.56 |  |
| B | 69 | t.127900 | 08/2007 | 08/2006 | 4.066 |  |
| B | 69 | t.26184 | 10/2006 | 08/2006 | 5.646 | 484 |
| B | 69 | t.125103 | 01/2010 | 09/2006 | 4.163 | 399 |
| B | 69 | t.142066 | 01/2009 | 09/2006 | 4.593 |  |
| B | 69 | t.26184 | 10/2006 | 09/2006 | 5.264 |  |
| B | 69 | t.46198 | 04/2007 | 09/2006 | 6.204 |  |
| B | 69 | t.73045 | 06/2007 | 09/2006 | 4.387 |  |
| B | 69 | t.26184 | 10/2006 | 10/2006 | 5.237 | 506 |
| B | 69 | t.73045 | 06/2007 | 12/2006 | 4.429 |  |
| B | 69 | t.125103 | 01/2010 | 02/2007 | 5.346 |  |
| B | 69 | t.20500 | 05/2007 | 03/2007 |  | 410 |
| B | 69 | t.46198 | 04/2007 | 03/2007 |  | 210 |
| B | 69 | t.73045 | 06/2007 | 03/2007 | 4.607 |  |
| B | 69 | t.73045 | 06/2007 | 06/2007 |  | 390 |
| B | 69 | t.127900 | 08/2007 | 07/2007 |  | 225 |
| B | 69 | t.57018 | 11/2008 | 04/2008 | 6.453 | 304 |
| B | 69 | t.57018 | 11/2008 | 06/2008 | 6.076 | 300 |
| B | 69 | t.57018 | 11/2008 | 08/2008 | 5.82 | 164 |
| B | 69 | t.142066 | 01/2009 | 11/2008 |  | 260 |
| B | 69 | t.113928 | 05/2013 | 06/2009 | 2.81 | 460 |
| B | 69 | t.86952 | 03/2010 | 06/2009 |  | 375 |
| B | 69 | t.69553 | 07/2010 | 07/2009 | 5.248 | 514 |
| B | 69 | t.69553 | 07/2010 | 09/2009 | 5.22 | 597 |
| B | 69 | t.113928 | 05/2013 | 10/2009 | 2.362 | 680 |
| B | 69 | t.86952 | 03/2010 | 10/2009 |  | 279 |
| B | 69 | t.86952 | 03/2010 | 11/2009 |  | 307 |
| B | 69 | t.93318 | 04/2010 | 11/2009 |  | 444 |
| B | 69 | t.125103 | 01/2010 | 12/2009 |  | 214 |
| B | 69 | t.69553 | 07/2010 | 12/2009 | 5.331 | 423 |
| B | 69 | t.86952 | 03/2010 | 12/2009 | 5.1 | 352 |
| B | 69 | t.113928 | 05/2013 | 02/2010 | 2.748 |  |
| B | 69 | t.93318 | 04/2010 | 03/2010 | 4.729 | 378 |
| B | 69 | t.69553 | 07/2010 | 05/2010 | 6.24 |  |
| B | 69 | t.69553 | 07/2010 | 07/2010 |  | 75 |
| B | 69 | t.113928 | 05/2013 | 09/2010 | 2.764 |  |
| B | 69 | t.135311 | 11/2010 | 10/2010 |  | 152 |
| B | 69 | t.113928 | 05/2013 | 02/2011 | 2.995 |  |
| B | 69 | t.44213 | 11/2012 | 09/2011 | 5.827 | 567 |
| B | 69 | t.44213 | 11/2012 | 10/2011 | 5.766 | 748 |
| B | 69 | t.27894 | 02/2012 | 01/2012 | 5.609 | 226 |
| B | 69 | t.27894 | 02/2012 | 02/2012 |  | 527 |
| B | 69 | t.141863 | 06/2012 | 03/2012 | 6.027 | 507 |
| B | 69 | t.141863 | 06/2012 | 04/2012 | 6.603 | 372 |
| B | 69 | t.141863 | 06/2012 | 06/2012 |  | 209 |
| B | 69 | t.76346 | 09/2012 | 06/2012 | 6.936 | 538 |
| B | 69 | t.46273 | 02/2013 | 08/2012 | 5.283 | 476 |
| B | 69 | t.76346 | 09/2012 | 08/2012 | 5.066 | 499 |
| B | 69 | t.46273 | 02/2013 | 09/2012 | 5.111 | 408 |
| B | 69 | t.46273 | 02/2013 | 10/2012 | 5.639 | 522 |
| B | 69 | t.44213 | 11/2012 | 11/2012 | 6.758 | 96 |
| B | 69 | t.46273 | 02/2013 | 01/2013 | 4.787 | 478 |
| B | 69 | t.46273 | 02/2013 | 02/2013 | 4.989 |  |
| B | 69 | t.113928 | 05/2013 | 04/2013 |  | 260 |
| B | 69 | t.97832 | 10/2013 | 08/2013 | 4.671 | 423 |
| B | 69 | t.97832 | 10/2013 | 10/2013 | 4.653 | 418 |
| B | 69 | t.19932 | 02/2015 | 09/2014 |  | 495 |
| B | 69 | t.19932 | 02/2015 | 10/2014 | 4.906 | 524 |
| B | 69 | t.19932 | 02/2015 | 02/2015 |  | 522 |
| B | 69 | t.58507 | 06/2017 | 06/2017 | 6.712 |  |
| B | 69 | t.60617 | 06/2017 | 06/2017 | 4.708 |  |
| B | 69 | t.78475 | 08/2017 | 07/2017 | 4.915 |  |
| B | 133 | t.95736 | 06/2007 | 11/1998 | 3.505 |  |
| B | 133 | t.95736 | 06/2007 | 08/2000 | 3.114 | 360 |
| B | 133 | t.95736 | 06/2007 | 01/2001 |  | 320 |
| B | 133 | t.105933 | 01/2007 | 04/2001 | 4.746 | 510 |
| B | 133 | t.53609 | 02/2011 | 04/2001 |  | 430 |
| B | 133 | t.95736 | 06/2007 | 07/2001 |  | 320 |
| B | 133 | t.105933 | 01/2007 | 08/2001 | 4.318 | 610 |
| B | 133 | t.105933 | 01/2007 | 10/2001 | 4.539 | 670 |
| B | 133 | t.105933 | 01/2007 | 01/2002 | 4.541 | 640 |
| B | 133 | t.105933 | 01/2007 | 04/2002 | 4.762 |  |
| B | 133 | t.105933 | 01/2007 | 09/2002 | 4.321 |  |
| B | 133 | t.53609 | 02/2011 | 11/2003 |  | 669 |
| B | 133 | t.56164 | 05/2007 | 12/2003 |  | 610 |
| B | 133 | t.56164 | 05/2007 | 03/2004 |  | 530 |
| B | 133 | t.102754 | 10/2005 | 04/2004 | 5.265 | 815 |
| B | 133 | t.102754 | 10/2005 | 05/2004 | 5.249 | 950 |
| B | 133 | t.102754 | 10/2005 | 06/2004 | 5.098 |  |
| B | 133 | t.56164 | 05/2007 | 06/2004 | 4.777 |  |
| B | 133 | t.102754 | 10/2005 | 08/2004 | 4.689 |  |
| B | 133 | t.56164 | 05/2007 | 09/2004 | 4.694 | 380 |
| B | 133 | t.102754 | 10/2005 | 11/2004 | 4.679 |  |
| B | 133 | t.53609 | 02/2011 | 11/2004 |  | 406 |
| B | 133 | t.56164 | 05/2007 | 12/2004 | 4.63 |  |
| B | 133 | t.102754 | 10/2005 | 02/2005 | 4.764 |  |
| B | 133 | t.53609 | 02/2011 | 02/2005 |  | 392 |
| B | 133 | t.102754 | 10/2005 | 05/2005 | 4.848 |  |
| B | 133 | t.102754 | 10/2005 | 07/2005 | 4.833 |  |
| B | 133 | t.102754 | 10/2005 | 09/2005 |  | 254 |
| B | 133 | t.102754 | 10/2005 | 10/2005 | 5.267 |  |
| B | 133 | t.56164 | 05/2007 | 11/2005 | 4.273 |  |
| B | 133 | t.56164 | 05/2007 | 12/2005 | 4.648 |  |
| B | 133 | t.29227 | 11/2008 | 04/2006 | 4.588 | 720 |
| B | 133 | t.29227 | 11/2008 | 07/2006 | 3.968 | 800 |
| B | 133 | t.29227 | 11/2008 | 11/2006 | 4.417 | 560 |
| B | 133 | t.105933 | 01/2007 | 01/2007 |  | 200 |
| B | 133 | t.127477 | 05/2009 | 02/2007 | 4.104 | 660 |
| B | 133 | t.29227 | 11/2008 | 03/2007 | 4.534 |  |
| B | 133 | t.54629 | 05/2008 | 03/2007 | 4.362 | 540 |
| B | 133 | t.54629 | 05/2008 | 04/2007 |  | 340 |
| B | 133 | t.56164 | 05/2007 | 04/2007 |  | 330 |
| B | 133 | t.95736 | 06/2007 | 05/2007 |  | 29 |
| B | 133 | t.127477 | 05/2009 | 06/2007 | 4.305 | 590 |
| B | 133 | t.29227 | 11/2008 | 08/2007 | 5.23 |  |
| B | 133 | t.127477 | 05/2009 | 09/2007 |  | 660 |
| B | 133 | t.4817 | 10/2010 | 09/2007 | 5.23 | 530 |
| B | 133 | t.54629 | 05/2008 | 09/2007 |  | 310 |
| B | 133 | t.38916 | 07/2008 | 11/2007 | 4.207 | 229 |
| B | 133 | t.38916 | 07/2008 | 12/2007 | 3.592 | 458 |
| B | 133 | t.4817 | 10/2010 | 12/2007 | 4.663 | 560 |
| B | 133 | t.54629 | 05/2008 | 01/2008 | 4.38 |  |
| B | 133 | t.29227 | 11/2008 | 03/2008 | 5.204 |  |
| B | 133 | t.38916 | 07/2008 | 03/2008 | 4.399 | 312 |
| B | 133 | t.54629 | 05/2008 | 03/2008 |  | 370 |
| B | 133 | t.38916 | 07/2008 | 05/2008 | 3.978 | 224 |
| B | 133 | t.23269 | 03/2012 | 07/2008 |  | 300 |
| B | 133 | t.29227 | 11/2008 | 11/2008 |  | 230 |
| B | 133 | t.133070 | 12/2010 | 02/2009 | 4.072 | 560 |
| B | 133 | t.133070 | 12/2010 | 04/2009 | 4.746 | 321 |
| B | 133 | t.127477 | 05/2009 | 05/2009 |  | 243 |
| B | 133 | t.133070 | 12/2010 | 06/2009 | 4.718 | 464 |
| B | 133 | t.4817 | 10/2010 | 07/2009 | 4.708 |  |
| B | 133 | t.44955 | 02/2014 | 08/2009 | 5.163 | 484 |
| B | 133 | t.99386 | 12/2009 | 08/2009 | 4.207 | 546 |
| B | 133 | t.133070 | 12/2010 | 09/2009 | 4.286 |  |
| B | 133 | t.99386 | 12/2009 | 09/2009 |  | 521 |
| B | 133 | t.99386 | 12/2009 | 10/2009 |  | 576 |
| B | 133 | t.23269 | 03/2012 | 11/2009 |  | 230 |
| B | 133 | t.99386 | 12/2009 | 11/2009 | 4.398 | 542 |
| B | 133 | t.133070 | 12/2010 | 12/2009 | 4.395 |  |
| B | 133 | t.133070 | 12/2010 | 03/2010 | 4.895 |  |
| B | 133 | t.27251 | 08/2013 | 06/2010 |  | 679 |
| B | 133 | t.133070 | 12/2010 | 07/2010 | 5.328 |  |
| B | 133 | t.4817 | 10/2010 | 10/2010 |  | 30 |
| B | 133 | t.133070 | 12/2010 | 11/2010 | 5.055 | 337 |
| B | 133 | t.80498 | 03/2011 | 01/2011 | 5.489 |  |
| B | 133 | t.80498 | 03/2011 | 02/2011 |  | 70 |
| B | 133 | t.80498 | 03/2011 | 03/2011 |  | 340 |
| B | 133 | t.110602 | 10/2013 | 09/2012 | 5.036 | 534 |
| B | 133 | t.110602 | 10/2013 | 10/2012 | 5.17 | 626 |
| B | 133 | t.110602 | 10/2013 | 02/2013 | 5.058 | 430 |
| B | 133 | t.43084 | 05/2013 | 02/2013 | 4.755 | 353 |
| B | 133 | t.43084 | 05/2013 | 03/2013 | 4.407 | 427 |
| B | 133 | t.43084 | 05/2013 | 04/2013 | 4.41 | 396 |
| B | 133 | t.110602 | 10/2013 | 06/2013 | 5.189 |  |
| B | 133 | t.27251 | 08/2013 | 07/2013 |  | 420 |
| B | 133 | t.110602 | 10/2013 | 08/2013 | 5.429 |  |
| B | 133 | t.110602 | 10/2013 | 10/2013 | 5.775 | 322 |
| B | 133 | t.44955 | 02/2014 | 01/2014 |  | 220 |
| B | 133 | t.26223 | 04/2016 | 03/2016 | 4.049 |  |
| B | 133 | t.47926 | 12/2016 | 05/2016 |  | 690 |
| B | 133 | t.47926 | 12/2016 | 07/2016 |  | 590 |
| B | 133 | t.47926 | 12/2016 | 08/2016 |  | 913 |
| B | 133 | t.47926 | 12/2016 | 12/2016 |  | 534 |
| B | 133 | t.88208 | 08/2018 | 08/2018 | 4.041 |  |

### Table S4. Number of *treestructure* phylotype assignments for different minimum clade sizes (30, 50, and 100) and bootstrap branch support values (90, 80, and without considering it) for the four analysed subtypes. The number of clusters resulting from the selected parameter choices of other partitioning methods (*fastbaps* and *treecluster*) are also presented.

|  | **Treestructure** | | | | | | | | | **Fastbaps**  **(optimise.symmetric)** | **Treecluster**  **(avg_clade method, genetic distance = 7.5%, bootstrap support = 80)** |
| --- | --- | --- | --- | --- | --- | --- | --- | --- | --- | --- | --- |
| **Subtype** | **Minimum clade size** | | | | | | | | |  |  |
|  | **30** | | | **50** | | | **100** | | |  |  |
|  | **Bootstrap support threshold (BT)** | | | | | | | | |  |  |
|  | **90** | **80** | **No BT** | **90** | **80** | **No BT** | **90** | **80** | **No BT** |  |  |
| **B** | 114 | 154 | 262 | 56 | 87 | 181 | 15 | 39 | 91 | 126 | 201 |
| **C** | 21 | 30 | 62 | 18 | 22 | 39 | 10 | 12 | 30 | 1 | 67 |
| **A1** | 12 | 12 | 25 | 11 | 11 | 14 | 5 | 6 | 9 | 26 | 54 |
| **CRF02AG** | 10 | 11 | 19 | 7 | 7 | 18 | 2 | 3 | 9 | 1 | 14 |

###

### Table S5. *Treestructure* paraphyletic phylotype identifiers for the three minimum clades sizes and four subtypes tested. These were resolved by tracing the MRCA of all sequences in the phylotype until a monophyletic group was formed. The backbone phylotypes are underlined and bolded. Quantity is indicated when the number of phylotypes is ≥ 5.

| **Min. clade size** | **Subtype** | | | |
| --- | --- | --- | --- | --- |
|  | **B** | **C** | **A1** | **CRF02AG** |
| **30** | 3,8,9,10,13,18,19,20,27,30,32,35,47,50,63,67,84,126,136,140,151,**153** (n=22) | 15,16,17,22,25,**29** (n=6) | 4,8,**11** | 4,9,**10** |
| **50** | 3,7,12,13,14,17,36,39,49,**86** (n=10) | 10,11,15,16,19,**21** (n=6) | 4,5,**8**,9 | **6**,7 |
| **100** | 3,6,12,14,15,26,**38** (n=7) | 3,6,7,**11**,12 (n=5) | 2,**5** | **2** |

###

### Table S6. Welch’s one-sided t-test comparing the pre-treatment mean viral load (in log_10_ copies per ml) for individuals within their respective phylotypes against the backbone phylotype based on the minimum clade size = 30 *treestructure* designations. Phylotypes with less than 10 individuals with viral load data available were excluded from this analysis. Estimates are in log_10_ copies/mL units. The table is ordered by increasing p-value (FDR-adjusted for multiple testing) and decreasing mean viral load estimate. Statistically significant FDR-adjusted (p<0.05) phylotypes with viral load estimates exceeding the backbone mean are bolded and underlined.

| **Subtype** | **Phylotype ID** | **Individuals with**  **pre-treatment**  **viral load** | **Viral load within**  **phylotype** | | **Viral load in backbone phylotype** | | **p-value**  **(unadjusted)** | **P-value**  **(Bonferroni)** | **p-value**  **(FDR)** |
| --- | --- | --- | --- | --- | --- | --- | --- | --- | --- |
|  |  |  | **Mean** | **SD** | **Mean** | **SD** |  |  |  |
| **B** | **40** | 38 | **5.110** | 0.713 | 4.570 | 0.783 | 0.00002 | 0.0023 | **0.0023** |
| **B** | **20** | 69 | **4.883** | 0.678 | 4.570 | 0.783 | 0.00016 | 0.018 | **0.0092** |
| **B** | **79** | 18 | **5.102** | 0.609 | 4.570 | 0.783 | 0.00088 | 0.1 | **0.034** |
| **B** | **125** | 11 | **5.287** | 0.621 | 4.570 | 0.783 | 0.0017 | 0.2 | **0.038** |
| **B** | **69** | 26 | **5.076** | 0.789 | 4.570 | 0.783 | 0.0016 | 0.18 | **0.038** |
| **B** | **137** | 19 | **4.955** | 0.507 | 4.570 | 0.783 | 0.002 | 0.23 | **0.038** |
| **C** | **18** | 12 | **5.076** | 0.669 | 4.462 | 0.866 | 0.0044 | 0.092 | **0.046** |
| **C** | **14** | 26 | **4.981** | 0.845 | 4.462 | 0.866 | 0.0023 | 0.048 | **0.046** |
| **B** | **4** | 13 | **5.112** | 0.585 | 4.570 | 0.783 | 0.0029 | 0.33 | **0.048** |
| CRF_02_AG | 5 | 22 | 5.143 | 0.943 | 4.564 | 0.905 | 0.0047 | 0.052 | 0.052 |
| B | 101 | 20 | 5.237 | 1.001 | 4.570 | 0.783 | 0.0039 | 0.45 | 0.056 |
| B | 122 | 13 | 5.177 | 0.740 | 4.570 | 0.783 | 0.006 | 0.69 | 0.077 |
| C | 13 | 12 | 4.823 | 0.468 | 4.462 | 0.866 | 0.011 | 0.23 | 0.077 |
| B | 103 | 13 | 5.011 | 0.554 | 4.570 | 0.783 | 0.0071 | 0.82 | 0.082 |
| C | 22 | 242 | 4.584 | 0.827 | 4.462 | 0.866 | 0.018 | 0.38 | 0.094 |
| C | 9 | 27 | 4.725 | 0.644 | 4.462 | 0.866 | 0.023 | 0.48 | 0.097 |
| B | 71 | 21 | 4.922 | 0.658 | 4.570 | 0.783 | 0.012 | 1.0 | 0.11 |
| B | 126 | 30 | 4.875 | 0.699 | 4.570 | 0.783 | 0.012 | 1.0 | 0.11 |
| B | 53 | 57 | 4.793 | 0.707 | 4.570 | 0.783 | 0.011 | 1.0 | 0.11 |
| B | 24 | 28 | 4.820 | 0.556 | 4.570 | 0.783 | 0.013 | 1.0 | 0.11 |
| B | 138 | 16 | 5.049 | 0.787 | 4.570 | 0.783 | 0.014 | 1.0 | 0.11 |
| B | 144 | 15 | 4.935 | 0.588 | 4.570 | 0.783 | 0.015 | 1.0 | 0.11 |
| B | 15 | 26 | 4.842 | 0.637 | 4.570 | 0.783 | 0.02 | 1.0 | 0.14 |
| B | 88 | 17 | 4.917 | 0.662 | 4.570 | 0.783 | 0.023 | 1.0 | 0.15 |
| B | 44 | 36 | 4.825 | 0.741 | 4.570 | 0.783 | 0.024 | 1.0 | 0.15 |
| B | 89 | 20 | 4.936 | 0.840 | 4.570 | 0.783 | 0.033 | 1.0 | 0.19 |
| B | 134 | 17 | 4.911 | 0.728 | 4.570 | 0.783 | 0.036 | 1.0 | 0.19 |
| B | 58 | 20 | 4.897 | 0.761 | 4.570 | 0.783 | 0.035 | 1.0 | 0.19 |
| B | 18 | 32 | 4.855 | 0.891 | 4.570 | 0.783 | 0.04 | 1.0 | 0.19 |
| B | 84 | 17 | 4.821 | 0.554 | 4.570 | 0.783 | 0.041 | 1.0 | 0.19 |
| B | 62 | 33 | 4.783 | 0.671 | 4.570 | 0.783 | 0.039 | 1.0 | 0.19 |
| B | 22 | 16 | 4.839 | 0.591 | 4.570 | 0.783 | 0.045 | 1.0 | 0.19 |
| B | 105 | 35 | 4.771 | 0.698 | 4.570 | 0.783 | 0.049 | 1.0 | 0.19 |
| B | 81 | 30 | 4.763 | 0.611 | 4.570 | 0.783 | 0.048 | 1.0 | 0.19 |
| B | 43 | 78 | 4.732 | 0.837 | 4.570 | 0.783 | 0.046 | 1.0 | 0.19 |
| B | 97 | 15 | 4.881 | 0.701 | 4.570 | 0.783 | 0.054 | 1.0 | 0.21 |
| B | 37 | 40 | 4.774 | 0.798 | 4.570 | 0.783 | 0.058 | 1.0 | 0.22 |
| C | 21 | 18 | 4.718 | 0.702 | 4.462 | 0.866 | 0.071 | 1.0 | 0.25 |
| B | 99 | 17 | 4.834 | 0.708 | 4.570 | 0.783 | 0.072 | 1.0 | 0.26 |
| B | 61 | 36 | 4.740 | 0.689 | 4.570 | 0.783 | 0.074 | 1.0 | 0.26 |
| B | 64 | 13 | 4.814 | 0.588 | 4.570 | 0.783 | 0.081 | 1.0 | 0.27 |
| B | 132 | 23 | 4.729 | 0.580 | 4.570 | 0.783 | 0.1 | 1.0 | 0.33 |
| C | 25 | 31 | 4.578 | 0.506 | 4.462 | 0.866 | 0.11 | 1.0 | 0.33 |
| B | 113 | 12 | 4.881 | 0.833 | 4.570 | 0.783 | 0.11 | 1.0 | 0.34 |
| B | 26 | 69 | 4.673 | 0.685 | 4.570 | 0.783 | 0.11 | 1.0 | 0.34 |
| CRF_02_AG | 6 | 19 | 4.877 | 0.901 | 4.564 | 0.905 | 0.076 | 0.84 | 0.42 |
| B | 68 | 13 | 4.741 | 0.548 | 4.570 | 0.783 | 0.14 | 1.0 | 0.42 |
| CRF_02_AG | 2 | 29 | 4.760 | 0.871 | 4.564 | 0.905 | 0.12 | 1.0 | 0.44 |
| B | 57 | 47 | 4.706 | 0.933 | 4.570 | 0.783 | 0.16 | 1.0 | 0.46 |
| B | 72 | 29 | 4.695 | 0.672 | 4.570 | 0.783 | 0.16 | 1.0 | 0.46 |
| C | 17 | 109 | 4.534 | 0.815 | 4.462 | 0.866 | 0.19 | 1.0 | 0.5 |
| CRF_02_AG | 1 | 63 | 4.678 | 0.978 | 4.564 | 0.905 | 0.19 | 1.0 | 0.52 |
| C | 15 | 32 | 4.536 | 0.588 | 4.462 | 0.866 | 0.25 | 1.0 | 0.58 |
| B | 10 | 21 | 4.732 | 0.951 | 4.570 | 0.783 | 0.22 | 1.0 | 0.59 |
| B | 45 | 24 | 4.706 | 0.797 | 4.570 | 0.783 | 0.21 | 1.0 | 0.59 |
| B | 140 | 16 | 4.706 | 0.687 | 4.570 | 0.783 | 0.22 | 1.0 | 0.59 |
| C | 16 | 67 | 4.526 | 0.956 | 4.462 | 0.866 | 0.3 | 1.0 | 0.63 |
| B | 128 | 11 | 4.828 | 1.223 | 4.570 | 0.783 | 0.25 | 1.0 | 0.64 |
| B | 117 | 23 | 4.704 | 0.949 | 4.570 | 0.783 | 0.25 | 1.0 | 0.64 |
| B | 5 | 133 | 4.611 | 0.718 | 4.570 | 0.783 | 0.26 | 1.0 | 0.65 |
| B | 77 | 12 | 4.693 | 0.669 | 4.570 | 0.783 | 0.27 | 1.0 | 0.66 |
| C | 28 | 15 | 4.510 | 0.733 | 4.462 | 0.866 | 0.4 | 1.0 | 0.7 |
| C | 3 | 35 | 4.495 | 0.784 | 4.462 | 0.866 | 0.4 | 1.0 | 0.7 |
| B | 48 | 14 | 4.668 | 0.700 | 4.570 | 0.783 | 0.3 | 1.0 | 0.72 |
| A_A1 | 6 | 22 | 4.553 | 1.021 | 4.402 | 0.891 | 0.25 | 1.0 | 0.72 |
| A_A1 | 4 | 60 | 4.495 | 0.772 | 4.402 | 0.891 | 0.19 | 1.0 | 0.72 |
| A_A1 | 5 | 10 | 4.399 | 0.462 | 4.402 | 0.891 | 0.51 | 1.0 | 0.72 |
| A_A1 | 8 | 40 | 4.373 | 0.877 | 4.402 | 0.891 | 0.58 | 1.0 | 0.72 |
| A_A1 | 3 | 77 | 4.369 | 0.746 | 4.402 | 0.891 | 0.64 | 1.0 | 0.72 |
| A_A1 | 11 | 17 | 4.347 | 0.757 | 4.402 | 0.891 | 0.61 | 1.0 | 0.72 |
| A_A1 | 12 | 17 | 4.347 | 0.757 | 4.402 | 0.891 | 0.61 | 1.0 | 0.72 |
| A_A1 | 1 | 12 | 4.344 | 0.852 | 4.402 | 0.891 | 0.59 | 1.0 | 0.72 |
| B | 123 | 23 | 4.640 | 0.694 | 4.570 | 0.783 | 0.32 | 1.0 | 0.74 |
| B | 90 | 19 | 4.627 | 0.511 | 4.570 | 0.783 | 0.32 | 1.0 | 0.74 |
| B | 115 | 21 | 4.634 | 0.649 | 4.570 | 0.783 | 0.33 | 1.0 | 0.74 |
| CRF_02_AG | 4 | 18 | 4.692 | 1.302 | 4.564 | 0.905 | 0.34 | 1.0 | 0.75 |
| A_A1 | 10 | 16 | 4.273 | 0.697 | 4.402 | 0.891 | 0.76 | 1.0 | 0.76 |
| B | 36 | 14 | 4.647 | 0.867 | 4.570 | 0.783 | 0.37 | 1.0 | 0.79 |
| B | 124 | 24 | 4.625 | 0.709 | 4.570 | 0.783 | 0.36 | 1.0 | 0.79 |
| B | 85 | 29 | 4.609 | 0.607 | 4.570 | 0.783 | 0.37 | 1.0 | 0.79 |
| C | 6 | 33 | 4.450 | 0.674 | 4.462 | 0.866 | 0.54 | 1.0 | 0.81 |
| C | 2 | 14 | 4.447 | 0.577 | 4.462 | 0.866 | 0.54 | 1.0 | 0.81 |
| C | 24 | 19 | 4.411 | 0.848 | 4.462 | 0.866 | 0.6 | 1.0 | 0.81 |
| C | 23 | 17 | 4.393 | 0.873 | 4.462 | 0.866 | 0.62 | 1.0 | 0.81 |
| B | 116 | 16 | 4.644 | 1.013 | 4.570 | 0.783 | 0.39 | 1.0 | 0.82 |
| C | 11 | 28 | 4.390 | 0.826 | 4.462 | 0.866 | 0.67 | 1.0 | 0.83 |
| B | 67 | 21 | 4.610 | 0.878 | 4.570 | 0.783 | 0.42 | 1.0 | 0.85 |
| B | 112 | 20 | 4.597 | 0.609 | 4.570 | 0.783 | 0.42 | 1.0 | 0.85 |
| B | 65 | 10 | 4.616 | 0.781 | 4.570 | 0.783 | 0.43 | 1.0 | 0.85 |
| B | 52 | 30 | 4.590 | 0.730 | 4.570 | 0.783 | 0.44 | 1.0 | 0.86 |
| B | 151 | 25 | 4.587 | 0.684 | 4.570 | 0.783 | 0.45 | 1.0 | 0.86 |
| B | 118 | 23 | 4.584 | 0.682 | 4.570 | 0.783 | 0.46 | 1.0 | 0.87 |
| B | 19 | 37 | 4.577 | 0.678 | 4.570 | 0.783 | 0.48 | 1.0 | 0.87 |
| B | 16 | 13 | 4.573 | 1.240 | 4.570 | 0.783 | 0.5 | 1.0 | 0.87 |
| B | 39 | 22 | 4.573 | 0.779 | 4.570 | 0.783 | 0.49 | 1.0 | 0.87 |
| B | 147 | 22 | 4.571 | 0.745 | 4.570 | 0.783 | 0.5 | 1.0 | 0.87 |
| B | 104 | 22 | 4.569 | 0.798 | 4.570 | 0.783 | 0.5 | 1.0 | 0.87 |
| B | 21 | 20 | 4.566 | 0.844 | 4.570 | 0.783 | 0.51 | 1.0 | 0.87 |
| B | 54 | 41 | 4.563 | 0.568 | 4.570 | 0.783 | 0.53 | 1.0 | 0.87 |
| B | 34 | 120 | 4.560 | 0.710 | 4.570 | 0.783 | 0.56 | 1.0 | 0.87 |
| B | 93 | 21 | 4.559 | 0.595 | 4.570 | 0.783 | 0.53 | 1.0 | 0.87 |
| B | 28 | 22 | 4.556 | 0.660 | 4.570 | 0.783 | 0.54 | 1.0 | 0.87 |
| B | 121 | 22 | 4.552 | 0.609 | 4.570 | 0.783 | 0.55 | 1.0 | 0.87 |
| B | 130 | 11 | 4.551 | 0.726 | 4.570 | 0.783 | 0.53 | 1.0 | 0.87 |
| B | 35 | 24 | 4.542 | 0.871 | 4.570 | 0.783 | 0.56 | 1.0 | 0.87 |
| B | 9 | 14 | 4.535 | 0.665 | 4.570 | 0.783 | 0.58 | 1.0 | 0.89 |
| B | 14 | 41 | 4.543 | 0.803 | 4.570 | 0.783 | 0.59 | 1.0 | 0.89 |
| B | 133 | 17 | 4.535 | 0.536 | 4.570 | 0.783 | 0.6 | 1.0 | 0.9 |
| C | 27 | 10 | 4.261 | 0.826 | 4.462 | 0.866 | 0.77 | 1.0 | 0.9 |
| B | 47 | 38 | 4.534 | 0.645 | 4.570 | 0.783 | 0.63 | 1.0 | 0.91 |
| B | 154 | 15 | 4.510 | 0.776 | 4.570 | 0.783 | 0.62 | 1.0 | 0.91 |
| B | 98 | 12 | 4.502 | 0.688 | 4.570 | 0.783 | 0.63 | 1.0 | 0.91 |
| B | 17 | 21 | 4.496 | 0.925 | 4.570 | 0.783 | 0.64 | 1.0 | 0.91 |
| CRF_02_AG | 9 | 41 | 4.560 | 0.776 | 4.564 | 0.905 | 0.51 | 1.0 | 0.91 |
| CRF_02_AG | 8 | 20 | 4.360 | 0.864 | 4.564 | 0.905 | 0.84 | 1.0 | 0.91 |
| CRF_02_AG | 3 | 17 | 4.316 | 0.744 | 4.564 | 0.905 | 0.9 | 1.0 | 0.91 |
| CRF_02_AG | 7 | 12 | 4.305 | 0.992 | 4.564 | 0.905 | 0.81 | 1.0 | 0.91 |
| CRF_02_AG | 10 | 12 | 4.175 | 0.936 | 4.564 | 0.905 | 0.91 | 1.0 | 0.91 |
| CRF_02_AG | 11 | 12 | 4.175 | 0.936 | 4.564 | 0.905 | 0.91 | 1.0 | 0.91 |
| B | 95 | 18 | 4.517 | 0.575 | 4.570 | 0.783 | 0.65 | 1.0 | 0.91 |
| B | 110 | 27 | 4.482 | 0.707 | 4.570 | 0.783 | 0.74 | 1.0 | 0.97 |
| B | 78 | 23 | 4.469 | 0.855 | 4.570 | 0.783 | 0.71 | 1.0 | 0.97 |
| B | 152 | 12 | 4.461 | 0.600 | 4.570 | 0.783 | 0.73 | 1.0 | 0.97 |
| B | 49 | 17 | 4.460 | 0.836 | 4.570 | 0.783 | 0.7 | 1.0 | 0.97 |
| B | 96 | 19 | 4.458 | 0.757 | 4.570 | 0.783 | 0.74 | 1.0 | 0.97 |
| B | 148 | 19 | 4.450 | 0.814 | 4.570 | 0.783 | 0.74 | 1.0 | 0.97 |
| B | 51 | 33 | 4.475 | 0.813 | 4.570 | 0.783 | 0.75 | 1.0 | 0.97 |
| B | 114 | 16 | 4.418 | 0.796 | 4.570 | 0.783 | 0.77 | 1.0 | 0.98 |
| B | 27 | 186 | 4.491 | 0.885 | 4.570 | 0.783 | 0.88 | 1.0 | 0.99 |
| B | 50 | 90 | 4.463 | 0.748 | 4.570 | 0.783 | 0.91 | 1.0 | 0.99 |
| B | 41 | 58 | 4.459 | 0.800 | 4.570 | 0.783 | 0.85 | 1.0 | 0.99 |
| B | 135 | 20 | 4.428 | 0.734 | 4.570 | 0.783 | 0.8 | 1.0 | 0.99 |
| B | 80 | 20 | 4.423 | 0.594 | 4.570 | 0.783 | 0.86 | 1.0 | 0.99 |
| B | 143 | 16 | 4.408 | 0.718 | 4.570 | 0.783 | 0.81 | 1.0 | 0.99 |
| B | 23 | 24 | 4.406 | 0.612 | 4.570 | 0.783 | 0.9 | 1.0 | 0.99 |
| B | 120 | 14 | 4.367 | 0.598 | 4.570 | 0.783 | 0.89 | 1.0 | 0.99 |
| B | 102 | 23 | 4.366 | 0.720 | 4.570 | 0.783 | 0.91 | 1.0 | 0.99 |
| B | 55 | 27 | 4.361 | 0.786 | 4.570 | 0.783 | 0.91 | 1.0 | 0.99 |
| B | 56 | 19 | 4.357 | 0.808 | 4.570 | 0.783 | 0.87 | 1.0 | 0.99 |
| B | 11 | 32 | 4.352 | 0.789 | 4.570 | 0.783 | 0.94 | 1.0 | 0.99 |
| B | 76 | 21 | 4.352 | 0.829 | 4.570 | 0.783 | 0.88 | 1.0 | 0.99 |
| B | 149 | 11 | 4.347 | 0.756 | 4.570 | 0.783 | 0.82 | 1.0 | 0.99 |
| B | 82 | 11 | 4.341 | 0.687 | 4.570 | 0.783 | 0.85 | 1.0 | 0.99 |
| B | 74 | 24 | 4.318 | 0.754 | 4.570 | 0.783 | 0.94 | 1.0 | 0.99 |
| B | 86 | 24 | 4.308 | 0.825 | 4.570 | 0.783 | 0.93 | 1.0 | 0.99 |
| B | 150 | 12 | 4.288 | 0.702 | 4.570 | 0.783 | 0.9 | 1.0 | 0.99 |
| B | 145 | 18 | 4.231 | 0.913 | 4.570 | 0.783 | 0.93 | 1.0 | 0.99 |
| B | 70 | 27 | 4.388 | 0.554 | 4.570 | 0.783 | 0.95 | 1.0 | 0.99 |
| B | 92 | 21 | 4.238 | 0.836 | 4.570 | 0.783 | 0.96 | 1.0 | 0.99 |
| B | 3 | 19 | 4.375 | 0.421 | 4.570 | 0.783 | 0.97 | 1.0 | 1.0 |
| B | 83 | 57 | 4.383 | 0.684 | 4.570 | 0.783 | 0.98 | 1.0 | 1.0 |
| B | 8 | 119 | 4.358 | 0.712 | 4.570 | 0.783 | 1.0 | 1.0 | 1.0 |
| C | 1 | 146 | 4.317 | 0.900 | 4.462 | 0.866 | 0.97 | 1.0 | 1.0 |
| B | 12 | 47 | 4.246 | 0.576 | 4.570 | 0.783 | 1.0 | 1.0 | 1.0 |
| C | 4 | 21 | 4.162 | 0.704 | 4.462 | 0.866 | 0.97 | 1.0 | 1.0 |
| C | 8 | 13 | 4.097 | 0.406 | 4.462 | 0.866 | 1.0 | 1.0 | 1.0 |

### Table S7. Estimates of a Bayesian model that jointly estimates differences in viral load between *treestructure* (minimum clade size = 30) phylotypes considering all the data (*i.e.* not against the backbone phylotype). The difference between all groups is assumed as following the same normal distribution. Estimates are in log_10_ copies/mL units. The table is ordered by increasing p-value and decreasing Bayesian estimate. Statistically significant (p<0.05) phylotypes with viral load estimates exceeding the population mean are bolded and underlined. A separate run was performed for each subtype and results merged for comparison only.

| **Subtype** | **Phylotype**  **ID** | **Lower**  **95% CI** | **Bayesian**  **estimate** | **Upper**  **95% CI** | **Mean**  **population**  **estimate** | **p-value** |
| --- | --- | --- | --- | --- | --- | --- |
| B | 8 | 4.276 | 4.403 | 4.528 | 4.588 | 0.0005 |
| **B** | **40** | 4.732 | **4.927** | 5.130 | 4.588 | **0.004** |
| B | 153 | 4.549 | 4.571 | 4.591 | 4.588 | 0.0075 |
| B | 12 | 4.180 | 4.374 | 4.564 | 4.588 | 0.0085 |
| **B** | **20** | 4.664 | **4.819** | 4.980 | 4.588 | **0.018** |
| **B** | **101** | 4.676 | **4.914** | 5.166 | 4.588 | **0.019** |
| B | 27 | 4.397 | 4.507 | 4.612 | 4.588 | 0.032 |
| **B** | **69** | 4.650 | **4.866** | 5.097 | 4.588 | **0.033** |
| B | 83 | 4.286 | 4.456 | 4.621 | 4.588 | 0.044 |
| C | 1 | 4.245 | 4.388 | 4.514 | 4.487 | 0.062 |
| B | 50 | 4.350 | 4.495 | 4.637 | 4.588 | 0.068 |
| B | 79 | 4.603 | 4.840 | 5.092 | 4.588 | 0.088 |
| B | 92 | 4.215 | 4.445 | 4.667 | 4.588 | 0.1 |
| B | 125 | 4.584 | 4.843 | 5.118 | 4.588 | 0.11 |
| B | 29 | 4.131 | 4.426 | 4.685 | 4.588 | 0.13 |
| B | 11 | 4.258 | 4.467 | 4.675 | 4.588 | 0.13 |
| B | 122 | 4.572 | 4.825 | 5.100 | 4.588 | 0.13 |
| B | 145 | 4.207 | 4.458 | 4.694 | 4.588 | 0.14 |
| B | 86 | 4.237 | 4.466 | 4.680 | 4.588 | 0.14 |
| B | 138 | 4.571 | 4.800 | 5.048 | 4.588 | 0.15 |
| B | 142 | 4.550 | 4.823 | 5.116 | 4.588 | 0.16 |
| C | 29 | 4.420 | 4.466 | 4.510 | 4.487 | 0.17 |
| B | 53 | 4.585 | 4.748 | 4.909 | 4.588 | 0.17 |
| B | 41 | 4.334 | 4.510 | 4.673 | 4.588 | 0.17 |
| B | 74 | 4.235 | 4.471 | 4.693 | 4.588 | 0.17 |
| B | 55 | 4.267 | 4.483 | 4.686 | 4.588 | 0.18 |
| B | 4 | 4.552 | 4.804 | 5.074 | 4.588 | 0.18 |
| B | 126 | 4.563 | 4.766 | 4.979 | 4.588 | 0.19 |
| C | 14 | 4.463 | 4.651 | 4.952 | 4.487 | 0.19 |
| B | 108 | 4.137 | 4.449 | 4.730 | 4.588 | 0.2 |
| B | 137 | 4.540 | 4.777 | 5.020 | 4.588 | 0.22 |
| B | 18 | 4.553 | 4.760 | 4.968 | 4.588 | 0.22 |
| CRF_02_AG | 5 | 4.533 | 4.763 | 5.194 | 4.583 | 0.23 |
| B | 119 | 4.534 | 4.783 | 5.070 | 4.588 | 0.23 |
| B | 70 | 4.285 | 4.499 | 4.703 | 4.588 | 0.24 |
| B | 71 | 4.533 | 4.768 | 5.006 | 4.588 | 0.24 |
| B | 89 | 4.535 | 4.771 | 5.017 | 4.588 | 0.24 |
| B | 44 | 4.546 | 4.749 | 4.950 | 4.588 | 0.24 |
| B | 102 | 4.267 | 4.499 | 4.720 | 4.588 | 0.25 |
| B | 76 | 4.245 | 4.498 | 4.733 | 4.588 | 0.27 |
| B | 103 | 4.511 | 4.768 | 5.039 | 4.588 | 0.29 |
| B | 56 | 4.267 | 4.504 | 4.741 | 4.588 | 0.29 |
| B | 58 | 4.523 | 4.753 | 4.988 | 4.588 | 0.3 |
| B | 23 | 4.29 | 4.514 | 4.735 | 4.588 | 0.31 |
| B | 15 | 4.526 | 4.743 | 4.968 | 4.588 | 0.31 |
| B | 88 | 4.514 | 4.751 | 4.994 | 4.588 | 0.31 |
| B | 43 | 4.564 | 4.708 | 4.854 | 4.588 | 0.31 |
| B | 3 | 4.266 | 4.517 | 4.760 | 4.588 | 0.34 |
| B | 134 | 4.511 | 4.749 | 4.991 | 4.588 | 0.34 |
| B | 24 | 4.512 | 4.735 | 4.967 | 4.588 | 0.35 |
| B | 37 | 4.527 | 4.723 | 4.915 | 4.588 | 0.35 |
| B | 144 | 4.505 | 4.752 | 5.003 | 4.588 | 0.36 |
| B | 51 | 4.334 | 4.539 | 4.742 | 4.588 | 0.37 |
| C | 4 | 4.18 | 4.437 | 4.611 | 4.487 | 0.37 |
| B | 150 | 4.247 | 4.512 | 4.762 | 4.588 | 0.38 |
| B | 34 | 4.444 | 4.572 | 4.692 | 4.588 | 0.38 |
| C | 18 | 4.416 | 4.606 | 4.921 | 4.487 | 0.39 |
| B | 105 | 4.509 | 4.718 | 4.923 | 4.588 | 0.39 |
| B | 46 | 4.469 | 4.752 | 5.061 | 4.588 | 0.4 |
| B | 62 | 4.518 | 4.719 | 4.932 | 4.588 | 0.41 |
| B | 30 | 4.465 | 4.748 | 5.065 | 4.588 | 0.41 |
| B | 127 | 4.203 | 4.512 | 4.796 | 4.588 | 0.42 |
| B | 120 | 4.272 | 4.529 | 4.776 | 4.588 | 0.42 |
| B | 25 | 4.474 | 4.741 | 5.020 | 4.588 | 0.42 |
| B | 7 | 4.459 | 4.744 | 5.061 | 4.588 | 0.42 |
| B | 80 | 4.297 | 4.534 | 4.764 | 4.588 | 0.42 |
| B | 135 | 4.300 | 4.536 | 4.778 | 4.588 | 0.42 |
| B | 97 | 4.487 | 4.728 | 4.969 | 4.588 | 0.43 |
| C | 8 | 4.175 | 4.453 | 4.641 | 4.487 | 0.44 |
| B | 81 | 4.500 | 4.706 | 4.905 | 4.588 | 0.45 |
| C | 22 | 4.473 | 4.567 | 4.667 | 4.487 | 0.46 |
| B | 99 | 4.478 | 4.715 | 4.961 | 4.588 | 0.46 |
| B | 110 | 4.332 | 4.551 | 4.765 | 4.588 | 0.46 |
| B | 82 | 4.27 | 4.534 | 4.782 | 4.588 | 0.47 |
| B | 143 | 4.291 | 4.541 | 4.780 | 4.588 | 0.47 |
| B | 22 | 4.475 | 4.714 | 4.963 | 4.588 | 0.47 |
| B | 100 | 4.246 | 4.528 | 4.806 | 4.588 | 0.47 |
| B | 114 | 4.300 | 4.543 | 4.773 | 4.588 | 0.48 |
| B | 149 | 4.260 | 4.540 | 4.803 | 4.588 | 0.49 |
| B | 78 | 4.320 | 4.553 | 4.777 | 4.588 | 0.49 |
| B | 148 | 4.310 | 4.549 | 4.780 | 4.588 | 0.49 |
| B | 113 | 4.470 | 4.72 | 4.985 | 4.588 | 0.5 |
| B | 84 | 4.473 | 4.708 | 4.954 | 4.588 | 0.5 |
| B | 59 | 4.442 | 4.726 | 5.032 | 4.588 | 0.5 |
| B | 61 | 4.499 | 4.698 | 4.900 | 4.588 | 0.5 |
| B | 38 | 4.440 | 4.727 | 5.025 | 4.588 | 0.51 |
| CRF_02_AG | 11 | 4.111 | 4.530 | 4.765 | 4.583 | 0.51 |
| CRF_02_AG | 6 | 4.470 | 4.665 | 5.019 | 4.583 | 0.51 |
| B | 96 | 4.308 | 4.557 | 4.785 | 4.588 | 0.52 |
| C | 9 | 4.415 | 4.575 | 4.808 | 4.487 | 0.53 |
| B | 66 | 4.257 | 4.547 | 4.811 | 4.588 | 0.55 |
| B | 107 | 4.215 | 4.537 | 4.851 | 4.588 | 0.56 |
| CRF_02_AG | 3 | 4.185 | 4.540 | 4.757 | 4.583 | 0.56 |
| B | 47 | 4.364 | 4.569 | 4.771 | 4.588 | 0.56 |
| B | 17 | 4.342 | 4.563 | 4.791 | 4.588 | 0.56 |
| B | 49 | 4.318 | 4.556 | 4.790 | 4.588 | 0.56 |
| B | 57 | 4.503 | 4.683 | 4.854 | 4.588 | 0.57 |
| B | 14 | 4.388 | 4.577 | 4.758 | 4.588 | 0.58 |
| C | 13 | 4.375 | 4.569 | 4.844 | 4.487 | 0.59 |
| CRF_02_AG | 8 | 4.207 | 4.544 | 4.766 | 4.583 | 0.59 |
| C | 12 | 4.380 | 4.570 | 4.886 | 4.487 | 0.6 |
| B | 64 | 4.439 | 4.696 | 4.954 | 4.588 | 0.61 |
| B | 75 | 4.409 | 4.702 | 5.006 | 4.588 | 0.62 |
| CRF_02_AG | 2 | 4.463 | 4.647 | 4.932 | 4.583 | 0.62 |
| B | 128 | 4.434 | 4.694 | 4.964 | 4.588 | 0.63 |
| B | 73 | 4.268 | 4.560 | 4.838 | 4.588 | 0.63 |
| C | 5 | 4.370 | 4.565 | 4.901 | 4.487 | 0.63 |
| CRF_02_AG | 7 | 4.178 | 4.550 | 4.787 | 4.583 | 0.64 |
| C | 7 | 4.368 | 4.568 | 4.893 | 4.487 | 0.64 |
| B | 54 | 4.397 | 4.588 | 4.774 | 4.588 | 0.65 |
| CRF_02_AG | 10 | 4.503 | 4.568 | 4.635 | 4.583 | 0.65 |
| B | 131 | 4.420 | 4.692 | 4.982 | 4.588 | 0.65 |
| C | 11 | 4.276 | 4.487 | 4.662 | 4.487 | 0.66 |
| B | 109 | 4.267 | 4.563 | 4.845 | 4.588 | 0.66 |
| B | 152 | 4.311 | 4.573 | 4.839 | 4.588 | 0.67 |
| B | 132 | 4.450 | 4.679 | 4.905 | 4.588 | 0.67 |
| C | 21 | 4.380 | 4.560 | 4.819 | 4.487 | 0.67 |
| B | 146 | 4.280 | 4.568 | 4.850 | 4.588 | 0.67 |
| B | 10 | 4.451 | 4.678 | 4.910 | 4.588 | 0.68 |
| B | 154 | 4.330 | 4.580 | 4.826 | 4.588 | 0.68 |
| B | 60 | 4.266 | 4.570 | 4.852 | 4.588 | 0.69 |
| CRF_02_AG | 1 | 4.464 | 4.633 | 4.835 | 4.583 | 0.69 |
| B | 42 | 4.305 | 4.574 | 4.834 | 4.588 | 0.7 |
| B | 26 | 4.501 | 4.660 | 4.821 | 4.588 | 0.7 |
| B | 35 | 4.363 | 4.588 | 4.810 | 4.588 | 0.7 |
| B | 95 | 4.346 | 4.583 | 4.817 | 4.588 | 0.71 |
| C | 27 | 4.242 | 4.489 | 4.696 | 4.487 | 0.71 |
| B | 133 | 4.338 | 4.588 | 4.828 | 4.588 | 0.72 |
| B | 13 | 4.279 | 4.578 | 4.869 | 4.588 | 0.73 |
| B | 94 | 4.279 | 4.577 | 4.860 | 4.588 | 0.73 |
| B | 45 | 4.444 | 4.669 | 4.893 | 4.588 | 0.73 |
| B | 121 | 4.363 | 4.590 | 4.815 | 4.588 | 0.74 |
| B | 87 | 4.307 | 4.587 | 4.877 | 4.588 | 0.74 |
| B | 72 | 4.464 | 4.664 | 4.873 | 4.588 | 0.74 |
| B | 98 | 4.331 | 4.587 | 4.841 | 4.588 | 0.74 |
| C | 23 | 4.272 | 4.493 | 4.699 | 4.487 | 0.75 |
| C | 6 | 4.306 | 4.496 | 4.673 | 4.487 | 0.75 |
| B | 28 | 4.374 | 4.594 | 4.816 | 4.588 | 0.75 |
| B | 117 | 4.438 | 4.666 | 4.899 | 4.588 | 0.76 |
| C | 24 | 4.285 | 4.497 | 4.689 | 4.487 | 0.76 |
| B | 19 | 4.389 | 4.600 | 4.800 | 4.588 | 0.76 |
| B | 129 | 4.284 | 4.585 | 4.890 | 4.588 | 0.77 |
| B | 68 | 4.417 | 4.667 | 4.932 | 4.588 | 0.77 |
| B | 21 | 4.364 | 4.598 | 4.828 | 4.588 | 0.78 |
| B | 104 | 4.369 | 4.600 | 4.822 | 4.588 | 0.78 |
| B | 9 | 4.342 | 4.593 | 4.845 | 4.588 | 0.79 |
| B | 39 | 4.381 | 4.600 | 4.832 | 4.588 | 0.79 |
| B | 93 | 4.365 | 4.597 | 4.823 | 4.588 | 0.79 |
| B | 63 | 4.287 | 4.591 | 4.883 | 4.588 | 0.79 |
| B | 140 | 4.418 | 4.662 | 4.909 | 4.588 | 0.79 |
| B | 147 | 4.374 | 4.601 | 4.820 | 4.588 | 0.8 |
| A_A1 | 4 | 4.324 | 4.422 | 4.571 | 4.409 | 0.8 |
| A_A1 | 7 | 4.300 | 4.423 | 4.646 | 4.409 | 0.81 |
| B | 31 | 4.358 | 4.667 | 4.984 | 4.588 | 0.81 |
| B | 52 | 4.392 | 4.606 | 4.811 | 4.588 | 0.82 |
| B | 5 | 4.496 | 4.615 | 4.735 | 4.588 | 0.83 |
| B | 111 | 4.355 | 4.662 | 4.966 | 4.588 | 0.84 |
| CRF_02_AG | 9 | 4.366 | 4.581 | 4.784 | 4.583 | 0.85 |
| B | 130 | 4.335 | 4.605 | 4.868 | 4.588 | 0.85 |
| B | 118 | 4.377 | 4.607 | 4.831 | 4.588 | 0.85 |
| A_A1 | 3 | 4.282 | 4.404 | 4.515 | 4.409 | 0.86 |
| B | 16 | 4.345 | 4.607 | 4.862 | 4.588 | 0.86 |
| CRF_02_AG | 4 | 4.376 | 4.615 | 4.920 | 4.583 | 0.86 |
| B | 151 | 4.385 | 4.611 | 4.820 | 4.588 | 0.86 |
| C | 2 | 4.284 | 4.508 | 4.723 | 4.487 | 0.86 |
| B | 91 | 4.305 | 4.604 | 4.896 | 4.588 | 0.86 |
| C | 26 | 4.249 | 4.508 | 4.753 | 4.487 | 0.87 |
| A_A1 | 6 | 4.293 | 4.419 | 4.609 | 4.409 | 0.87 |
| A_A1 | 10 | 4.237 | 4.406 | 4.544 | 4.409 | 0.87 |
| B | 77 | 4.400 | 4.650 | 4.909 | 4.588 | 0.88 |
| C | 3 | 4.331 | 4.512 | 4.689 | 4.487 | 0.88 |
| C | 25 | 4.367 | 4.536 | 4.741 | 4.487 | 0.88 |
| B | 32 | 4.381 | 4.650 | 4.918 | 4.588 | 0.89 |
| B | 112 | 4.399 | 4.614 | 4.841 | 4.588 | 0.9 |
| C | 20 | 4.278 | 4.510 | 4.763 | 4.487 | 0.9 |
| A_A1 | 11 | 4.340 | 4.407 | 4.473 | 4.409 | 0.91 |
| B | 48 | 4.393 | 4.642 | 4.895 | 4.588 | 0.91 |
| B | 141 | 4.369 | 4.644 | 4.921 | 4.588 | 0.91 |
| B | 85 | 4.403 | 4.618 | 4.831 | 4.588 | 0.91 |
| A_A1 | 8 | 4.273 | 4.408 | 4.535 | 4.409 | 0.92 |
| B | 136 | 4.352 | 4.641 | 4.926 | 4.588 | 0.92 |
| B | 67 | 4.391 | 4.619 | 4.842 | 4.588 | 0.93 |
| A_A1 | 1 | 4.253 | 4.409 | 4.557 | 4.409 | 0.93 |
| B | 123 | 4.422 | 4.637 | 4.851 | 4.588 | 0.93 |
| B | 33 | 4.319 | 4.618 | 4.921 | 4.588 | 0.94 |
| A_A1 | 12 | 4.261 | 4.409 | 4.548 | 4.409 | 0.94 |
| B | 106 | 4.345 | 4.619 | 4.889 | 4.588 | 0.94 |
| B | 1 | 4.324 | 4.642 | 4.967 | 4.588 | 0.94 |
| C | 17 | 4.405 | 4.527 | 4.662 | 4.487 | 0.95 |
| B | 65 | 4.348 | 4.622 | 4.899 | 4.588 | 0.95 |
| C | 19 | 4.281 | 4.515 | 4.768 | 4.487 | 0.96 |
| C | 28 | 4.307 | 4.518 | 4.745 | 4.487 | 0.96 |
| C | 16 | 4.375 | 4.519 | 4.675 | 4.487 | 0.97 |
| A_A1 | 9 | 4.247 | 4.411 | 4.568 | 4.409 | 0.97 |
| B | 116 | 4.390 | 4.634 | 4.882 | 4.588 | 0.97 |
| B | 36 | 4.383 | 4.633 | 4.889 | 4.588 | 0.97 |
| C | 30 | 4.280 | 4.517 | 4.764 | 4.487 | 0.97 |
| C | 10 | 4.275 | 4.522 | 4.790 | 4.487 | 0.98 |
| B | 2 | 4.326 | 4.627 | 4.930 | 4.588 | 0.99 |
| B | 115 | 4.407 | 4.632 | 4.853 | 4.588 | 0.99 |
| B | 124 | 4.401 | 4.629 | 4.857 | 4.588 | 0.99 |
| B | 90 | 4.388 | 4.627 | 4.863 | 4.588 | 0.99 |
| C | 15 | 4.347 | 4.524 | 4.719 | 4.487 | 0.99 |
| A_A1 | 2 | 4.262 | 4.413 | 4.586 | 4.409 | 0.99 |
| A_A1 | 5 | 4.265 | 4.412 | 4.568 | 4.409 | 0.99 |
| B | 6 | 4.294 | 4.629 | 4.970 | 4.588 | 1.0 |
| B | 139 | 4.299 | 4.628 | 4.969 | 4.588 | 1.0 |

###

### Table S8. Phylotype regression coefficients (effect on slope, *i.e.* rate of CD4 decline per year) from the maximum likelihood mixed-effects model of CD4 cell decline with a random effect across different phylotypes (estimated using *treestructure*, minimum clade size = 30) and different individuals and fixed effects on risk group, age group, years since first CD4, sex, and relevant interactions of each phylotype against respective backbone phylotype. The reference category for fixed effects is: MSMs in the backbone phylotype in their thirties (30-39 age group). The model was run for 1000 iterations using bootstrap resampled data to estimate phylotype-specific random effect p-values. Estimates are in cells/mm^3^/year units. The table is ordered by increasing p-value and CD4 regression estimate (CD4 decline). Statistically significant (p<0.05) phylotypes associated with a decline from a two-tailed test of CD4 declines are bolded and underlined. A separate run was performed for each subtype and results merged for comparison only. Random effect variability and correlation structure parameters are shown at the bottom of this table.

| **Subtype** | **Phylotype ID** | **Regression coefficient (cells/mm^3^/year)** | **Standard error** | **T-value** | **p-value** |
| --- | --- | --- | --- | --- | --- |
| **B** | **133** | **-10.182** | 2.479 | -4.108 | **0.00004** |
| B | 74 | 14.595 | 3.765 | 3.876 | 0.00011 |
| B | 57 | 12.321 | 3.379 | 3.647 | 0.00027 |
| **B** | **69** | **-7.978** | 2.626 | -3.038 | **0.0024** |
| **B** | **90** | **-7.987** | 2.72 | -2.937 | **0.0033** |
| **B** | **118** | **-8.773** | 3.039 | -2.886 | **0.0039** |
| CRF_02_AG | 9 | 8.066 | 2.838 | 2.842 | 0.0045 |
| A_A1 | 11 | 15.020 | 5.335 | 2.815 | 0.0049 |
| B | 149 | 5.662 | 2.023 | 2.799 | 0.0051 |
| **CRF_02_AG** | **7** | **-9.919** | 3.611 | -2.747 | **0.006** |
| B | 45 | 8.786 | 3.573 | 2.459 | 0.014 |
| **A_A1** | **3** | **-15.564** | 6.658 | -2.338 | **0.019** |
| **A_A1** | **8** | **-7.336** | 3.117 | -2.353 | **0.019** |
| **B** | **137** | **-8.610** | 3.713 | -2.319 | **0.02** |
| B | 44 | 4.677 | 2.143 | 2.183 | 0.029 |
| **B** | **84** | **-8.173** | 3.764 | -2.172 | **0.03** |
| **B** | **24** | **-7.638** | 3.59 | -2.128 | **0.033** |
| **B** | **77** | **-3.687** | 1.735 | -2.126 | **0.034** |
| A_A1 | 6 | 10.875 | 5.211 | 2.087 | 0.037 |
| B | 50 | 6.055 | 2.974 | 2.036 | 0.042 |
| B | 39 | 4.227 | 2.132 | 1.983 | 0.047 |
| **B** | **62** | **-5.907** | 3.014 | -1.96 | **0.05** |
| B | 140 | 4.551 | 2.391 | 1.904 | 0.057 |
| B | 56 | 6.419 | 3.376 | 1.902 | 0.057 |
| B | 153 | 5.420 | 2.898 | 1.87 | 0.061 |
| B | 16 | 6.988 | 3.736 | 1.871 | 0.061 |
| B | 8 | 7.660 | 4.095 | 1.871 | 0.061 |
| B | 26 | -2.729 | 1.471 | -1.855 | 0.064 |
| B | 28 | -8.170 | 4.51 | -1.811 | 0.07 |
| B | 97 | 5.092 | 2.809 | 1.812 | 0.07 |
| B | 65 | -4.887 | 2.724 | -1.794 | 0.073 |
| B | 147 | 8.634 | 4.913 | 1.757 | 0.079 |
| B | 49 | 5.485 | 3.132 | 1.751 | 0.08 |
| B | 48 | 5.207 | 3.029 | 1.719 | 0.086 |
| B | 151 | -9.320 | 5.538 | -1.683 | 0.092 |
| B | 132 | -8.518 | 5.141 | -1.657 | 0.098 |
| B | 76 | 4.356 | 2.638 | 1.652 | 0.099 |
| B | 83 | -8.561 | 5.221 | -1.64 | 0.1 |
| B | 40 | -6.159 | 3.772 | -1.633 | 0.1 |
| B | 92 | 5.724 | 3.527 | 1.623 | 0.1 |
| CRF_02_AG | 4 | -5.519 | 3.678 | -1.501 | 0.13 |
| B | 14 | 6.270 | 4.104 | 1.528 | 0.13 |
| B | 54 | -7.057 | 4.939 | -1.429 | 0.15 |
| B | 21 | -4.217 | 2.915 | -1.447 | 0.15 |
| B | 102 | 4.474 | 3.137 | 1.426 | 0.15 |
| B | 10 | 8.822 | 6.323 | 1.395 | 0.16 |
| B | 43 | -4.716 | 3.517 | -1.341 | 0.18 |
| CRF_02_AG | 10 | 8.929 | 6.629 | 1.347 | 0.18 |
| B | 36 | 5.719 | 4.708 | 1.215 | 0.22 |
| B | 106 | -4.722 | 3.911 | -1.207 | 0.23 |
| B | 12 | 3.851 | 3.193 | 1.206 | 0.23 |
| A_A1 | 5 | -4.852 | 4.163 | -1.165 | 0.24 |
| B | 145 | 3.058 | 2.595 | 1.178 | 0.24 |
| B | 23 | 3.249 | 2.739 | 1.186 | 0.24 |
| B | 27 | 5.669 | 4.857 | 1.167 | 0.24 |
| B | 25 | -3.696 | 3.216 | -1.149 | 0.25 |
| B | 58 | 5.561 | 4.809 | 1.156 | 0.25 |
| B | 37 | -6.102 | 5.403 | -1.129 | 0.26 |
| B | 72 | 3.392 | 3.038 | 1.117 | 0.26 |
| A_A1 | 4 | -4.616 | 4.159 | -1.11 | 0.27 |
| B | 127 | 5.458 | 4.932 | 1.107 | 0.27 |
| B | 22 | -3.889 | 3.569 | -1.09 | 0.28 |
| B | 67 | -5.540 | 5.263 | -1.053 | 0.29 |
| B | 99 | -3.220 | 3.063 | -1.051 | 0.29 |
| B | 80 | 2.434 | 2.321 | 1.049 | 0.29 |
| B | 95 | -3.554 | 3.44 | -1.033 | 0.3 |
| B | 142 | -3.112 | 3.004 | -1.036 | 0.3 |
| B | 52 | 4.289 | 4.346 | 0.987 | 0.32 |
| B | 66 | 3.438 | 3.65 | 0.942 | 0.35 |
| B | 148 | -4.644 | 5.085 | -0.913 | 0.36 |
| B | 124 | -4.096 | 4.454 | -0.92 | 0.36 |
| B | 125 | -4.051 | 4.452 | -0.91 | 0.36 |
| B | 109 | 3.920 | 4.398 | 0.891 | 0.37 |
| B | 144 | 4.526 | 5.092 | 0.889 | 0.37 |
| B | 64 | 2.964 | 3.395 | 0.873 | 0.38 |
| B | 51 | -1.482 | 1.769 | -0.838 | 0.4 |
| B | 101 | 1.432 | 1.718 | 0.833 | 0.4 |
| B | 114 | 3.479 | 4.216 | 0.825 | 0.41 |
| CRF_02_AG | 11 | 4.060 | 4.926 | 0.824 | 0.41 |
| B | 55 | -3.186 | 4.076 | -0.782 | 0.43 |
| B | 60 | 4.173 | 5.521 | 0.756 | 0.45 |
| B | 94 | 1.592 | 2.152 | 0.74 | 0.46 |
| CRF_02_AG | 6 | -1.965 | 2.719 | -0.723 | 0.47 |
| B | 120 | 3.220 | 4.488 | 0.717 | 0.47 |
| B | 116 | -2.368 | 3.431 | -0.69 | 0.49 |
| B | 100 | 2.851 | 4.146 | 0.688 | 0.49 |
| B | 87 | 2.509 | 3.716 | 0.675 | 0.5 |
| B | 96 | -3.849 | 5.789 | -0.665 | 0.51 |
| B | 3 | -2.055 | 3.133 | -0.656 | 0.51 |
| B | 13 | 0.921 | 1.406 | 0.655 | 0.51 |
| B | 128 | 1.895 | 2.849 | 0.665 | 0.51 |
| B | 5 | -3.690 | 5.706 | -0.647 | 0.52 |
| B | 138 | -1.954 | 3.142 | -0.622 | 0.53 |
| B | 11 | 2.094 | 3.342 | 0.627 | 0.53 |
| B | 82 | 2.171 | 3.463 | 0.627 | 0.53 |
| B | 152 | -1.654 | 2.715 | -0.609 | 0.54 |
| B | 15 | 2.082 | 3.403 | 0.612 | 0.54 |
| B | 20 | -3.415 | 5.677 | -0.602 | 0.55 |
| B | 59 | -3.021 | 5.026 | -0.601 | 0.55 |
| B | 19 | -1.773 | 2.934 | -0.604 | 0.55 |
| C | 22 | -0.785 | 1.325 | -0.592 | 0.55 |
| A_A1 | 1 | 2.499 | 4.224 | 0.592 | 0.55 |
| B | 2 | 0.848 | 1.453 | 0.584 | 0.56 |
| B | 61 | 2.367 | 4.09 | 0.578 | 0.56 |
| CRF_02_AG | 8 | 2.605 | 4.469 | 0.583 | 0.56 |
| B | 18 | 1.323 | 2.373 | 0.558 | 0.58 |
| B | 121 | 1.733 | 3.107 | 0.558 | 0.58 |
| B | 71 | 2.450 | 4.459 | 0.549 | 0.58 |
| B | 129 | 2.892 | 5.208 | 0.555 | 0.58 |
| B | 35 | 2.754 | 5.144 | 0.535 | 0.59 |
| B | 143 | -2.067 | 3.998 | -0.517 | 0.61 |
| B | 47 | -1.356 | 2.751 | -0.493 | 0.62 |
| A_A1 | 7 | 1.627 | 3.273 | 0.497 | 0.62 |
| B | 103 | -2.560 | 5.33 | -0.48 | 0.63 |
| B | 119 | 1.765 | 3.617 | 0.488 | 0.63 |
| B | 98 | 2.436 | 5.014 | 0.486 | 0.63 |
| B | 85 | -3.194 | 7.039 | -0.454 | 0.65 |
| B | 110 | -2.203 | 4.84 | -0.455 | 0.65 |
| B | 88 | -1.497 | 3.321 | -0.451 | 0.65 |
| B | 122 | -2.518 | 5.755 | -0.438 | 0.66 |
| CRF_02_AG | 1 | -1.707 | 4.074 | -0.419 | 0.68 |
| B | 112 | -3.147 | 8.203 | -0.384 | 0.7 |
| B | 131 | -2.298 | 6.092 | -0.377 | 0.71 |
| B | 53 | -2.157 | 5.786 | -0.373 | 0.71 |
| B | 89 | -0.431 | 1.175 | -0.367 | 0.71 |
| B | 135 | 1.298 | 3.611 | 0.359 | 0.72 |
| B | 107 | 1.145 | 3.373 | 0.339 | 0.73 |
| B | 141 | -1.220 | 3.663 | -0.333 | 0.74 |
| CRF_02_AG | 3 | -2.930 | 9.658 | -0.303 | 0.76 |
| CRF_02_AG | 5 | -2.217 | 7.132 | -0.311 | 0.76 |
| B | 63 | 1.177 | 4.042 | 0.291 | 0.77 |
| A_A1 | 10 | 1.195 | 4.068 | 0.294 | 0.77 |
| B | 115 | 1.482 | 5.243 | 0.283 | 0.78 |
| C | 24 | -0.082 | 0.313 | -0.261 | 0.79 |
| B | 136 | -1.906 | 7.351 | -0.259 | 0.8 |
| B | 42 | -1.744 | 6.853 | -0.255 | 0.8 |
| B | 104 | 1.600 | 6.397 | 0.25 | 0.8 |
| C | 26 | 0.100 | 0.448 | 0.223 | 0.82 |
| B | 68 | 0.938 | 4.022 | 0.233 | 0.82 |
| B | 130 | -1.003 | 4.716 | -0.213 | 0.83 |
| C | 29 | 1.062 | 5 | 0.212 | 0.83 |
| B | 111 | 0.629 | 3.382 | 0.186 | 0.85 |
| B | 31 | -1.341 | 7.515 | -0.178 | 0.86 |
| C | 16 | -0.167 | 0.954 | -0.175 | 0.86 |
| B | 7 | -0.847 | 4.984 | -0.17 | 0.87 |
| B | 34 | -0.728 | 4.314 | -0.169 | 0.87 |
| B | 117 | -0.600 | 3.641 | -0.165 | 0.87 |
| C | 11 | 0.190 | 1.147 | 0.166 | 0.87 |
| CRF_02_AG | 2 | 0.598 | 3.662 | 0.163 | 0.87 |
| B | 73 | 0.600 | 3.893 | 0.154 | 0.88 |
| B | 38 | 0.824 | 5.367 | 0.153 | 0.88 |
| A_A1 | 2 | 0.889 | 5.878 | 0.151 | 0.88 |
| B | 70 | -0.707 | 4.9 | -0.144 | 0.89 |
| C | 14 | -0.168 | 1.177 | -0.143 | 0.89 |
| C | 27 | 0.145 | 1.031 | 0.141 | 0.89 |
| C | 21 | -0.275 | 2.307 | -0.119 | 0.91 |
| C | 6 | -0.090 | 0.757 | -0.119 | 0.91 |
| C | 12 | 0.223 | 2.024 | 0.11 | 0.91 |
| C | 1 | -0.223 | 2.164 | -0.103 | 0.92 |
| B | 79 | -0.185 | 1.732 | -0.107 | 0.92 |
| B | 29 | 0.280 | 2.634 | 0.106 | 0.92 |
| B | 134 | 0.377 | 3.744 | 0.101 | 0.92 |
| B | 30 | 0.477 | 4.788 | 0.1 | 0.92 |
| B | 108 | -0.350 | 3.993 | -0.088 | 0.93 |
| B | 9 | -0.334 | 3.597 | -0.093 | 0.93 |
| B | 46 | -0.314 | 3.712 | -0.085 | 0.93 |
| C | 17 | 0.105 | 1.136 | 0.093 | 0.93 |
| B | 123 | -0.250 | 3.618 | -0.069 | 0.94 |
| B | 78 | -0.246 | 3.074 | -0.08 | 0.94 |
| C | 15 | -0.166 | 2.386 | -0.07 | 0.94 |
| C | 23 | 0.060 | 0.945 | 0.063 | 0.95 |
| B | 17 | -0.257 | 4.637 | -0.055 | 0.96 |
| C | 2 | -0.095 | 1.925 | -0.049 | 0.96 |
| C | 19 | 0.073 | 1.61 | 0.046 | 0.96 |
| C | 4 | 0.163 | 3.714 | 0.044 | 0.96 |
| A_A1 | 12 | 0.237 | 4.681 | 0.051 | 0.96 |
| B | 32 | -0.150 | 4.627 | -0.032 | 0.97 |
| C | 7 | 0.038 | 0.935 | 0.041 | 0.97 |
| C | 10 | 0.038 | 1.02 | 0.037 | 0.97 |
| C | 8 | 0.103 | 2.847 | 0.036 | 0.97 |
| B | 105 | 0.172 | 4.834 | 0.036 | 0.97 |
| C | 18 | -0.037 | 1.62 | -0.023 | 0.98 |
| C | 20 | -0.018 | 0.929 | -0.019 | 0.98 |
| C | 13 | -0.016 | 0.828 | -0.02 | 0.98 |
| B | 93 | 0.180 | 6.821 | 0.026 | 0.98 |
| B | 41 | -0.024 | 2.743 | -0.009 | 0.99 |
| C | 30 | -0.018 | 0.959 | -0.019 | 0.99 |
| C | 5 | -0.013 | 1.568 | -0.008 | 0.99 |
| C | 9 | 0.020 | 1.182 | 0.017 | 0.99 |
| A_A1 | 9 | 0.026 | 3.021 | 0.009 | 0.99 |
| C | 3 | 0.032 | 2.921 | 0.011 | 0.99 |
| B | 113 | 0.038 | 4.589 | 0.008 | 0.99 |
| B | 91 | 0.051 | 4.107 | 0.012 | 1.0 |
| B | 126 | 0.128 | 7.058 | 0.018 | 0.99 |
| **Random Effect Variability and Correlation Structure** | | | | | |
| **Subtype** | **Parameter** | | **Mean across replicates** | **SD across replicates** |  |
| A_A1 | Phylotype Intercept SD | | 46.613 | 4.862 |  |
|  | Phylotype Slope SD | | 14.192 | 3.730 |  |
|  | Phylotype Intercept–Slope Correlation | | -0.996 | 0.071 |  |
|  | Individual Intercept SD | | 212.274 | 4.095 |  |
|  | Individual Slope SD | | 51.990 | 9.029 |  |
|  | Individual Intercept–Slope Correlation | | -0.441 | 0.050 |  |
|  | Residual Standard Deviation | | 71.516 | 4.253 |  |
| CRF_02_AG | Phylotype Intercept SD | | 64.819 | 9.521 |  |
|  | Phylotype Slope SD | | 8.332 | 5.676 |  |
|  | Phylotype Intercept–Slope Correlation | | -0.818 | 0.555 |  |
|  | Individual Intercept SD | | 220.452 | 4.087 |  |
|  | Individual Slope SD | | 58.84 | 7.332 |  |
|  | Individual Intercept–Slope Correlation | | -0.473 | 0.051 |  |
|  | Residual Standard Deviation | | 77.791 | 4.127 |  |

| C | Phylotype Intercept SD | 31.524 | 11.413 |  |
| --- | --- | --- | --- | --- |
|  | Phylotype Slope SD | 3.119 | 2.012 |  |
|  | Phylotype Intercept–Slope Correlation | -0.068 | 0.832 |  |
|  | Individual Intercept SD | 201.247 | 2.217 |  |
|  | Individual Slope SD | 41.463 | 4.249 |  |
|  | Individual Intercept–Slope Correlation | -0.453 | 0.03 |  |
|  | Residual Standard Deviation | 69.108 | 3.035 |  |
| B | Phylotype Intercept SD | 26.249 | 2.466 |  |
|  | Phylotype Slope SD | 11.425 | 1.771 |  |
|  | Phylotype Intercept–Slope Correlation | 0.572 | 0.127 |  |
|  | Individual Intercept SD | 217.265 | 1.096 |  |
|  | Individual Slope SD | 57.827 | 1.92 |  |
|  | Individual Intercept–Slope Correlation | -0.444 | 0.013 |  |
|  | Residual Standard Deviation | 82.752 | 1.164 |  |

### Table S9. Phylotype regression coefficients (effect on slope, *i.e.* rate of CD4 decline per year) from the sensitivity analysis using a Bayesian mixed-effects model of CD4 cell decline with a random effect across different phylotypes (estimated using *treestructure*, minimum clade size = 30) and different individuals and fixed effects on risk group, age group, years since first CD4, sex, and relevant interactions of each phylotype against respective backbone phylotype. The reference category for fixed effects is: MSMs in the backbone phylotype in their thirties (30-39 age group). Estimates are in cells/mm^3^/year units. The table is ordered by decreasing posterior probability of having a CD4 slope more negative than the backbone (PP). Phylotypes with PP > 80% are bolded and underlined. A separate run was performed for each subtype and results merged for comparison only. Random effect variability and correlation structure parameters are shown at the bottom of this table.

| **Subtype** | **Phylotype ID** | **Lower 95% CI** | **Regression coefficient (cells/mm^3^/year)** | **Upper 95% CI** | **Estimated error** | **Bayesian p-value** | **PP** |
| --- | --- | --- | --- | --- | --- | --- | --- |
| **A_A1** | **3** | -26.850 | **-11.003** | 2.002 | 7.199 | 0.088 | **0.956** |
| **B** | **133** | -26.288 | **-10.94** | 1.565 | 7.37 | 0.11 | **0.945** |
| **B** | **83** | -19.544 | **-8.475** | 2.65 | 5.572 | 0.14 | **0.928** |
| **B** | **90** | -23.974 | **-9.161** | 4.372 | 7.11 | 0.17 | **0.915** |
| **B** | **118** | -21.983 | **-8.946** | 3.153 | 6.608 | 0.17 | **0.915** |
| **B** | **28** | -21.085 | **-7.858** | 4.137 | 6.635 | 0.21 | **0.896** |
| **B** | **24** | -21.953 | **-7.902** | 5.268 | 6.81 | 0.21 | **0.895** |
| **B** | **54** | -22.375 | **-7.552** | 4.556 | 6.678 | 0.23 | **0.887** |
| **B** | **137** | -23.161 | **-8.381** | 5.01 | 6.917 | 0.23 | **0.885** |
| **B** | **151** | -22.585 | **-8.294** | 5.407 | 7.229 | 0.25 | **0.877** |
| **A_A1** | **8** | -25.615 | **-8.565** | 6.766 | 8.016 | 0.27 | **0.865** |
| **B** | **69** | -23.046 | **-7.97** | 4.938 | 7.224 | 0.27 | **0.865** |
| **B** | **132** | -24.714 | **-8.2** | 6.089 | 7.654 | 0.27 | **0.863** |
| **B** | **84** | -22.443 | **-7.651** | 5.698 | 7.234 | 0.28 | **0.86** |
| **B** | **150** | -20.437 | **-6.951** | 5.185 | 6.814 | 0.3 | **0.848** |
| **B** | **62** | -20.256 | **-6.464** | 6.497 | 6.608 | 0.31 | **0.847** |
| **B** | **5** | -11.916 | **-3.905** | 3.99 | 4.053 | 0.33 | **0.833** |
| **B** | **37** | -20.597 | **-5.949** | 6.197 | 6.696 | 0.36 | **0.819** |
| **B** | **40** | -18.784 | **-5.353** | 8.15 | 6.818 | 0.39 | **0.806** |
| **B** | **154** | -20.068 | **-5.86** | 7.594 | 6.981 | 0.39 | **0.805** |
| B | 67 | -17.069 | -5.185 | 7.089 | 6.338 | 0.41 | 0.796 |
| CRF_02_AG | 1 | -25.865 | -5.96 | 5.245 | 7.951 | 0.42 | 0.789 |
| CRF_02_AG | 7 | -40.851 | -8.59 | 7.304 | 11.942 | 0.42 | 0.788 |
| B | 43 | -15.893 | -4.51 | 6.21 | 5.568 | 0.42 | 0.788 |
| B | 65 | -20.339 | -5.373 | 8.716 | 7.705 | 0.48 | 0.758 |
| B | 95 | -18.145 | -4.707 | 7.724 | 6.702 | 0.49 | 0.756 |
| B | 148 | -19.986 | -4.718 | 10.189 | 7.298 | 0.49 | 0.753 |
| B | 21 | -18.366 | -4.789 | 7.952 | 6.869 | 0.5 | 0.749 |
| B | 124 | -17.626 | -4.57 | 7.416 | 6.479 | 0.51 | 0.747 |
| B | 85 | -16.725 | -4.004 | 7.517 | 6.248 | 0.53 | 0.733 |
| B | 55 | -17.609 | -4.185 | 8.253 | 6.735 | 0.54 | 0.729 |
| B | 112 | -16.388 | -3.601 | 8.625 | 6.208 | 0.55 | 0.727 |
| B | 22 | -18.658 | -4.333 | 9.788 | 7.232 | 0.55 | 0.726 |
| C | 22 | -7.352 | -1.386 | 2.056 | 2.241 | 0.57 | 0.717 |
| B | 106 | -21.304 | -4.482 | 9.949 | 7.803 | 0.59 | 0.707 |
| B | 96 | -16.379 | -3.696 | 9.436 | 6.536 | 0.59 | 0.707 |
| A_A1 | 5 | -23.563 | -4.62 | 10.358 | 8.586 | 0.59 | 0.705 |
| B | 77 | -18.841 | -3.797 | 9.849 | 7.346 | 0.6 | 0.702 |
| B | 99 | -18.020 | -3.452 | 10.239 | 7.079 | 0.61 | 0.696 |
| B | 25 | -17.371 | -3.322 | 11.099 | 7.284 | 0.63 | 0.687 |
| B | 142 | -19.940 | -3.58 | 10.88 | 7.661 | 0.63 | 0.685 |
| B | 125 | -18.602 | -3.353 | 12.128 | 7.952 | 0.67 | 0.667 |
| B | 26 | -13.586 | -2.281 | 9.093 | 5.663 | 0.67 | 0.667 |
| B | 131 | -18.103 | -3.202 | 10.661 | 7.537 | 0.67 | 0.663 |
| A_A1 | 4 | -16.079 | -2.675 | 10.431 | 6.931 | 0.7 | 0.651 |
| B | 20 | -14.840 | -2.481 | 9.436 | 6.144 | 0.7 | 0.648 |
| B | 19 | -13.711 | -2.083 | 8.612 | 5.676 | 0.72 | 0.641 |
| B | 116 | -17.303 | -2.688 | 11.905 | 7.176 | 0.72 | 0.638 |
| B | 81 | -14.038 | -2.355 | 8.134 | 5.756 | 0.72 | 0.638 |
| B | 42 | -20.028 | -2.877 | 10.504 | 7.603 | 0.73 | 0.634 |
| C | 21 | -8.899 | -1.012 | 3.886 | 3.114 | 0.76 | 0.621 |
| B | 59 | -19.834 | -2.781 | 12.93 | 8.29 | 0.76 | 0.62 |
| B | 88 | -15.973 | -1.912 | 11.063 | 6.859 | 0.77 | 0.616 |
| B | 3 | -16.661 | -2.407 | 9.511 | 6.595 | 0.77 | 0.614 |
| CRF_02_AG | 3 | -21.722 | -2.524 | 11.487 | 8.29 | 0.79 | 0.606 |
| B | 34 | -8.999 | -0.971 | 7.236 | 4.1 | 0.79 | 0.606 |
| B | 110 | -12.724 | -1.438 | 9.824 | 5.725 | 0.79 | 0.605 |
| B | 136 | -17.881 | -1.925 | 14.034 | 7.841 | 0.79 | 0.604 |
| B | 103 | -15.603 | -1.856 | 12.833 | 7.004 | 0.8 | 0.601 |
| B | 75 | -16.329 | -1.579 | 13.186 | 7.795 | 0.8 | 0.6 |
| B | 130 | -14.206 | -1.389 | 12.024 | 6.704 | 0.81 | 0.594 |
| B | 143 | -16.867 | -1.963 | 12.462 | 7.333 | 0.82 | 0.592 |
| B | 122 | -16.615 | -1.579 | 13.414 | 7.458 | 0.82 | 0.591 |
| B | 117 | -14.008 | -1.479 | 9.944 | 5.933 | 0.82 | 0.588 |
| B | 47 | -12.304 | -1.209 | 9.671 | 5.622 | 0.83 | 0.586 |
| B | 152 | -14.604 | -1.215 | 12.536 | 6.743 | 0.83 | 0.585 |
| C | 25 | -7.073 | -0.657 | 4.679 | 2.787 | 0.83 | 0.585 |
| C | 14 | -7.281 | -0.631 | 4.581 | 2.678 | 0.83 | 0.583 |
| B | 4 | -15.880 | -1.488 | 12.691 | 7.57 | 0.84 | 0.582 |
| B | 138 | -17.592 | -1.633 | 14.063 | 7.484 | 0.84 | 0.579 |
| B | 146 | -17.333 | -1.605 | 13.689 | 7.821 | 0.85 | 0.576 |
| B | 53 | -12.673 | -0.897 | 11.412 | 5.874 | 0.86 | 0.569 |
| B | 141 | -15.063 | -0.828 | 14.231 | 7.527 | 0.86 | 0.568 |
| B | 7 | -17.105 | -1.04 | 15.059 | 8.143 | 0.88 | 0.56 |
| B | 70 | -12.860 | -0.851 | 11.425 | 6.178 | 0.88 | 0.56 |
| C | 2 | -6.738 | -0.38 | 4.642 | 2.798 | 0.88 | 0.56 |
| B | 51 | -12.651 | -0.899 | 10.708 | 5.925 | 0.89 | 0.557 |
| B | 73 | -18.131 | -1.158 | 15.296 | 8.356 | 0.89 | 0.555 |
| B | 31 | -18.267 | -1.123 | 15.735 | 8.639 | 0.9 | 0.548 |
| C | 15 | -5.540 | -0.164 | 5.198 | 2.523 | 0.91 | 0.545 |
| B | 41 | -11.013 | -0.594 | 9.537 | 5.077 | 0.91 | 0.544 |
| A_A1 | 12 | -17.868 | -1.052 | 16.361 | 8.402 | 0.92 | 0.54 |
| C | 6 | -6.955 | -0.311 | 5.074 | 2.709 | 0.92 | 0.538 |
| A_A1 | 9 | -21.282 | -0.878 | 18.074 | 10.011 | 0.94 | 0.528 |
| C | 30 | -5.877 | -0.132 | 5.667 | 2.711 | 0.94 | 0.528 |
| B | 79 | -15.296 | -0.52 | 13.938 | 7.513 | 0.95 | 0.525 |
| C | 3 | -6.415 | -0.194 | 5.347 | 2.71 | 0.96 | 0.519 |
| B | 29 | -15.570 | -0.586 | 13.627 | 7.15 | 0.96 | 0.518 |
| C | 24 | -6.287 | -0.292 | 4.927 | 2.591 | 0.96 | 0.518 |
| C | 28 | -6.148 | -0.172 | 5.255 | 2.786 | 0.96 | 0.518 |
| B | 108 | -12.347 | -0.09 | 13.514 | 6.708 | 0.96 | 0.518 |
| C | 20 | -5.827 | 0.068 | 6.216 | 2.848 | 0.97 | 0.514 |
| B | 89 | -13.443 | -0.488 | 11.769 | 6.293 | 0.98 | 0.51 |
| C | 18 | -7.020 | -0.125 | 6.193 | 3 | 0.98 | 0.509 |
| C | 7 | -6.290 | -0.063 | 6.108 | 2.979 | 0.99 | 0.507 |
| B | 9 | -15.150 | -0.187 | 14.951 | 7.649 | 0.99 | 0.506 |
| C | 5 | -6.090 | 0 | 6.575 | 3.095 | 0.99 | 0.503 |
| B | 134 | -13.997 | -0.195 | 13.758 | 7.063 | 1 | 0.502 |
| CRF_02_AG | 5 | -19.475 | -0.247 | 18.676 | 8.64 | 1 | 0.499 |
| B | 46 | -16.641 | -0.191 | 14.695 | 8.152 | 1 | 0.499 |
| C | 16 | -6.202 | 0.074 | 6.299 | 2.789 | 1 | 0.499 |
| C | 4 | -6.185 | -0.035 | 6.339 | 3.052 | 0.99 | 0.496 |
| B | 32 | -14.591 | 0.339 | 14.589 | 7.558 | 0.99 | 0.495 |
| C | 19 | -4.986 | 0.22 | 7.108 | 2.807 | 0.97 | 0.487 |
| B | 91 | -15.067 | 0.287 | 15.624 | 7.817 | 0.96 | 0.482 |
| C | 1 | -4.819 | 0.102 | 4.795 | 2.213 | 0.96 | 0.479 |
| B | 93 | -12.971 | 0.31 | 12.933 | 6.516 | 0.96 | 0.479 |
| C | 10 | -5.274 | 0.176 | 6.12 | 2.789 | 0.95 | 0.477 |
| C | 8 | -5.914 | 0.094 | 5.847 | 2.657 | 0.95 | 0.475 |
| B | 30 | -14.925 | 0.899 | 16.644 | 8.088 | 0.95 | 0.475 |
| B | 105 | -12.089 | 0.048 | 12.067 | 6.017 | 0.95 | 0.474 |
| B | 78 | -13.042 | 0.393 | 14.063 | 6.904 | 0.95 | 0.474 |
| B | 101 | -13.843 | 0.515 | 14.4 | 7.21 | 0.95 | 0.473 |
| C | 13 | -5.509 | 0.301 | 6.795 | 2.981 | 0.94 | 0.47 |
| B | 38 | -14.324 | 0.621 | 15.987 | 7.494 | 0.93 | 0.465 |
| B | 123 | -11.909 | 0.605 | 14.901 | 6.668 | 0.92 | 0.462 |
| B | 86 | -12.691 | 0.726 | 14.002 | 6.759 | 0.92 | 0.459 |
| C | 23 | -5.470 | 0.238 | 6.27 | 2.895 | 0.91 | 0.457 |
| A_A1 | 10 | -13.722 | 1.018 | 16.013 | 7.759 | 0.91 | 0.456 |
| C | 11 | -5.128 | 0.269 | 5.989 | 2.505 | 0.91 | 0.455 |
| CRF_02_AG | 4 | -14.919 | 1.243 | 22.092 | 8.363 | 0.9 | 0.45 |
| A_A1 | 1 | -20.848 | 1.482 | 24.656 | 10.853 | 0.89 | 0.444 |
| B | 2 | -13.701 | 1.093 | 16.629 | 7.652 | 0.88 | 0.439 |
| C | 9 | -5.204 | 0.368 | 6.832 | 2.782 | 0.88 | 0.438 |
| C | 27 | -4.697 | 0.442 | 6.554 | 2.556 | 0.87 | 0.437 |
| C | 17 | -4.060 | 0.358 | 5.506 | 2.234 | 0.87 | 0.436 |
| B | 113 | -11.857 | 1.56 | 17.301 | 7.525 | 0.87 | 0.436 |
| A_A1 | 7 | -15.476 | 1.296 | 19.99 | 8.657 | 0.87 | 0.435 |
| B | 13 | -15.036 | 1.043 | 17.345 | 8.026 | 0.87 | 0.434 |
| B | 126 | -10.850 | 1.331 | 14.642 | 6.604 | 0.87 | 0.434 |
| CRF_02_AG | 6 | -13.096 | 1.545 | 18.954 | 7.406 | 0.86 | 0.43 |
| B | 135 | -11.605 | 1.213 | 13.602 | 6.48 | 0.86 | 0.428 |
| B | 94 | -14.698 | 1.51 | 17.505 | 8.105 | 0.86 | 0.428 |
| B | 18 | -13.765 | 0.821 | 13.858 | 6.89 | 0.85 | 0.427 |
| B | 111 | -13.135 | 1.372 | 17.581 | 7.71 | 0.85 | 0.426 |
| B | 68 | -14.368 | 1.45 | 16.552 | 7.696 | 0.85 | 0.424 |
| C | 26 | -4.516 | 0.521 | 6.974 | 2.743 | 0.85 | 0.423 |
| B | 115 | -13.116 | 1.3 | 15.384 | 7.1 | 0.85 | 0.423 |
| CRF_02_AG | 8 | -12.873 | 1.824 | 20.779 | 7.81 | 0.85 | 0.423 |
| B | 104 | -11.157 | 1.42 | 13.827 | 6.381 | 0.83 | 0.416 |
| B | 121 | -10.389 | 1.299 | 12.819 | 5.82 | 0.83 | 0.415 |
| B | 107 | -14.822 | 1.376 | 17.891 | 8.272 | 0.83 | 0.415 |
| B | 63 | -12.934 | 1.664 | 16.816 | 7.852 | 0.82 | 0.412 |
| B | 17 | -10.615 | 1.577 | 14.324 | 6.706 | 0.82 | 0.41 |
| B | 119 | -12.604 | 1.769 | 16.735 | 7.392 | 0.81 | 0.403 |
| CRF_02_AG | 10 | -9.254 | 1.683 | 14.913 | 6.035 | 0.8 | 0.402 |
| A_A1 | 2 | -16.640 | 2.198 | 21.086 | 9.17 | 0.8 | 0.402 |
| B | 11 | -10.749 | 1.564 | 14.202 | 6.27 | 0.79 | 0.396 |
| B | 128 | -13.206 | 2.15 | 16.923 | 7.694 | 0.77 | 0.387 |
| C | 12 | -3.897 | 1.07 | 8.415 | 3.144 | 0.77 | 0.383 |
| B | 82 | -11.637 | 2.389 | 17.555 | 7.388 | 0.75 | 0.376 |
| B | 100 | -12.439 | 2.388 | 18.218 | 7.583 | 0.74 | 0.372 |
| B | 145 | -10.012 | 1.976 | 13.432 | 6.025 | 0.74 | 0.369 |
| B | 87 | -10.634 | 2.745 | 17.625 | 7.275 | 0.73 | 0.367 |
| B | 61 | -9.783 | 1.907 | 13.824 | 5.891 | 0.73 | 0.364 |
| B | 98 | -10.654 | 2.627 | 15.935 | 6.694 | 0.69 | 0.346 |
| B | 71 | -9.046 | 2.959 | 15.038 | 6.36 | 0.66 | 0.331 |
| B | 15 | -9.696 | 3 | 16.731 | 6.673 | 0.66 | 0.328 |
| CRF_02_AG | 2 | -9.999 | 3.055 | 20.291 | 7.09 | 0.65 | 0.326 |
| B | 35 | -8.819 | 3.033 | 15.647 | 6.241 | 0.65 | 0.325 |
| B | 129 | -11.076 | 3.286 | 19.277 | 7.537 | 0.65 | 0.325 |
| B | 23 | -10.479 | 3.206 | 17.34 | 7.047 | 0.64 | 0.318 |
| B | 80 | -7.844 | 3.165 | 15.613 | 6.125 | 0.63 | 0.315 |
| B | 64 | -10.376 | 3.346 | 18.98 | 7.296 | 0.63 | 0.313 |
| CRF_02_AG | 9 | -8.958 | 3.794 | 21.034 | 7.481 | 0.62 | 0.312 |
| B | 120 | -8.411 | 3.322 | 16.113 | 6.459 | 0.62 | 0.31 |
| B | 66 | -9.694 | 3.882 | 20.043 | 7.39 | 0.61 | 0.305 |
| B | 60 | -10.380 | 4.365 | 20.051 | 7.782 | 0.59 | 0.295 |
| CRF_02_AG | 11 | -9.226 | 4.822 | 27.407 | 8.872 | 0.58 | 0.292 |
| B | 114 | -8.375 | 3.913 | 16.933 | 6.522 | 0.57 | 0.284 |
| B | 33 | -10.186 | 4.463 | 19.965 | 7.827 | 0.57 | 0.284 |
| B | 102 | -9.453 | 3.628 | 17.629 | 6.674 | 0.56 | 0.282 |
| B | 12 | -6.586 | 3.532 | 14.167 | 5.39 | 0.54 | 0.269 |
| B | 72 | -7.580 | 3.438 | 14.914 | 5.647 | 0.53 | 0.265 |
| B | 52 | -7.438 | 3.642 | 15.314 | 5.802 | 0.52 | 0.258 |
| B | 109 | -8.917 | 4.582 | 19.414 | 7.191 | 0.51 | 0.253 |
| B | 58 | -8.751 | 4.837 | 19.084 | 7.174 | 0.5 | 0.251 |
| B | 140 | -7.223 | 5.086 | 20.162 | 7.016 | 0.5 | 0.25 |
| B | 127 | -8.438 | 5.401 | 21.293 | 7.588 | 0.49 | 0.246 |
| B | 144 | -8.023 | 4.792 | 19.616 | 6.982 | 0.47 | 0.235 |
| B | 76 | -6.759 | 4.393 | 17.284 | 6.106 | 0.47 | 0.234 |
| B | 39 | -7.546 | 4.908 | 18.214 | 6.513 | 0.44 | 0.218 |
| B | 14 | -8.239 | 5.058 | 18.815 | 6.596 | 0.42 | 0.208 |
| B | 48 | -8.105 | 5.723 | 19.906 | 7.168 | 0.42 | 0.208 |
| B | 97 | -7.234 | 5.327 | 18.422 | 6.496 | 0.41 | 0.205 |
| B | 149 | -7.955 | 5.611 | 19.825 | 6.846 | 0.39 | 0.197 |
| B | 56 | -6.887 | 5.783 | 18.974 | 6.73 | 0.39 | 0.193 |
| A_A1 | 6 | -9.555 | 8.636 | 30.378 | 10.077 | 0.38 | 0.189 |
| B | 49 | -6.023 | 5.864 | 18.993 | 6.373 | 0.36 | 0.181 |
| B | 44 | -5.526 | 5.228 | 16.342 | 5.706 | 0.35 | 0.175 |
| B | 92 | -6.256 | 5.967 | 19.781 | 6.543 | 0.35 | 0.173 |
| B | 36 | -6.162 | 6.349 | 19.974 | 6.831 | 0.34 | 0.168 |
| B | 16 | -6.271 | 7.147 | 22.559 | 7.5 | 0.33 | 0.166 |
| C | 29 | -0.931 | 1.704 | 5.969 | 1.86 | 0.31 | 0.155 |
| B | 45 | -5.485 | 7.999 | 20.523 | 6.669 | 0.2 | 0.101 |
| B | 10 | -3.418 | 8.836 | 21.44 | 6.457 | 0.17 | 0.084 |
| B | 147 | -2.765 | 8.316 | 20.348 | 5.837 | 0.15 | 0.077 |
| B | 50 | -1.577 | 6.152 | 15.029 | 4.175 | 0.13 | 0.066 |
| B | 8 | -0.333 | 7.454 | 16.17 | 4.497 | 0.068 | 0.034 |
| B | 27 | -0.274 | 5.839 | 11.975 | 3.127 | 0.06 | 0.03 |
| B | 57 | 2.920 | 12.11 | 21.788 | 4.932 | 0.012 | 0.006 |
| B | 74 | 2.510 | 15.537 | 31.042 | 7.122 | 0.012 | 0.006 |
| A_A1 | 11 | 4.973 | 14.803 | 26.802 | 5.591 | 0.006 | 0.003 |
| B | 153 | 2.493 | 5.477 | 8.464 | 1.511 | 0 | 0 |
| **Random Effect Variability and Correlation Structure** | | | | | |  |  |
| **Subtype** | **Parameter** | | **Lower 95% CI** | **Estimate** | **Upper 95% CI** |  |  |
| A_A1 | Phylotype Intercept SD | | 24.058 | 46.472 | 79.479 |  |  |
|  | Phylotype Slope SD | | 5.079 | 10.936 | 19.575 |  |  |
|  | Phylotype Intercept–Slope Correlation | | -0.947 | -0.569 | 0.08 |  |  |
|  | Individual Intercept SD | | 186.012 | 197.963 | 210.835 |  |  |
|  | Individual Slope SD | | 17.944 | 22.127 | 26.346 |  |  |
|  | Individual Intercept–Slope Correlation | | -0.662 | -0.544 | -0.409 |  |  |
|  | Residual Standard Deviation | | 99.000 | 102.945 | 107.213 |  |  |

| CRF_02_AG | Phylotype Intercept SD | 28.96 | 61.566 | 104.476 |  |  |
| --- | --- | --- | --- | --- | --- | --- |
|  | Phylotype Slope SD | 0.352 | 8.225 | 21.531 |  |  |
|  | Phylotype Intercept–Slope Correlation | -0.849 | -0.235 | 0.567 |  |  |
|  | Individual Intercept SD | 194.598 | 207.582 | 221.365 |  |  |
|  | Individual Slope SD | 27.997 | 33.821 | 40.211 |  |  |
|  | Individual Intercept–Slope Correlation | -0.579 | -0.442 | -0.292 |  |  |
|  | Residual Standard Deviation | 102.756 | 107.206 | 111.938 |  |  |
| C | Phylotype Intercept SD | 10.889 | 35.433 | 61.227 |  |  |
|  | Phylotype Slope SD | 0.171 | 2.453 | 6.136 |  |  |
|  | Phylotype Intercept–Slope Correlation | -0.809 | -0.043 | 0.723 |  |  |
|  | Individual Intercept SD | 183.502 | 189.988 | 196.711 |  |  |
|  | Individual Slope SD | 20.115 | 22.286 | 24.553 |  |  |
|  | Individual Intercept–Slope Correlation | -0.558 | -0.484 | -0.399 |  |  |
|  | Residual Standard Deviation | 92.182 | 94.487 | 96.793 |  |  |
| B | Phylotype Intercept SD | 17.701 | 28.882 | 39.09 |  |  |
|  | Phylotype Slope SD | 5.284 | 8.117 | 11.34 |  |  |
|  | Phylotype Intercept–Slope Correlation | -0.087 | 0.357 | 0.771 |  |  |
|  | Individual Intercept SD | 197.433 | 200.889 | 204.319 |  |  |
|  | Individual Slope SD | 29.627 | 31.048 | 32.385 |  |  |
|  | Individual Intercept–Slope Correlation | -0.494 | -0.457 | -0.42 |  |  |
|  | Residual Standard Deviation | 113.115 | 114.353 | 115.625 |  |  |

### Table S10. Phylotype regression coefficients (effect on slope, *i.e.* rate of CD4 decline per year) from the sensitivity analysis using a Bayesian mixed-effects model of CD4 cell decline with a random effect across different individuals and fixed effects across different phylotypes using the R2-D2 prior (estimated using *treestructure*, minimum clade size = 30) and risk group, age group, years since first CD4, sex, and relevant interactions of each phylotype against respective backbone phylotype. The reference category for fixed effects is: MSMs in the backbone phylotype in their thirties (30-39 age group). Estimates are in cells/mm^3^/year units. The table is ordered by decreasing posterior probability of having a negative slope and increasing CD4 regression estimate (CD4 decline). Phylotypes with >80% posterior probability of having a negative CD4 decline (negative coefficient) are bolded and underlined. A separate run was performed for each subtype and results merged for comparison only. Random effect variability and correlation structure parameters are shown at the bottom of this table.

| **Subtype** | **Phylotype ID** | **Lower 95% CI** | **Regression coefficient (cells/mm3/year)** | **Upper 95% CI** | **Estimated error** | **Bayesian p-value** | **Posterior probability of having negative slope** |
| --- | --- | --- | --- | --- | --- | --- | --- |
| **A_A1** | **3** | -37.517 | **-18.661** | -1.189 | 9.266 | **0.032** | **0.984** |
| **B** | **133** | -56.402 | **-29.089** | -0.319 | 14.595 | **0.033** | **0.983** |
| **A_A1** | **8** | -42.126 | **-21.265** | 0.177 | 11.421 | 0.06 | **0.97** |
| **B** | **90** | -44.396 | **-19.579** | 0.901 | 12.417 | 0.09 | **0.955** |
| **B** | **83** | -29.842 | **-12.767** | 1.285 | 8.197 | 0.12 | **0.941** |
| **B** | **54** | -36.137 | **-14.227** | 2.226 | 10.79 | 0.14 | **0.927** |
| **C** | **22** | -11.027 | **-4.475** | 1.037 | 3.338 | 0.16 | **0.917** |
| **B** | **118** | -38.923 | **-15.729** | 3.448 | 11.666 | 0.17 | **0.916** |
| **B** | **28** | -39.760 | **-14.916** | 3.052 | 11.663 | 0.17 | **0.915** |
| **B** | **5** | -15.421 | **-5.916** | 1.311 | 4.65 | 0.19 | **0.905** |
| **B** | **69** | -44.778 | **-15.745** | 2.616 | 13.369 | 0.19 | **0.903** |
| **B** | **151** | -43.730 | **-15.566** | 3.627 | 13.181 | 0.2 | **0.901** |
| **B** | **150** | -40.481 | **-13.649** | 3.947 | 11.829 | 0.22 | **0.892** |
| **B** | **137** | -40.709 | **-14.44** | 4.31 | 12.398 | 0.22 | **0.891** |
| **B** | **24** | -39.391 | **-12.867** | 5.166 | 11.584 | 0.24 | **0.882** |
| **B** | **37** | -34.405 | **-11.319** | 4.189 | 10.67 | 0.26 | **0.873** |
| **C** | **21** | -32.541 | **-10.3** | 4.077 | 9.877 | 0.26 | **0.871** |
| **B** | **132** | -51.370 | **-16.238** | 4.701 | 15.3 | 0.26 | **0.868** |
| **B** | **62** | -34.132 | **-10.562** | 4.614 | 10.315 | 0.27 | **0.864** |
| **B** | **43** | -20.553 | **-6.731** | 3.561 | 6.541 | 0.27 | **0.864** |
| **B** | **84** | -53.778 | **-15.704** | 6.156 | 15.852 | 0.29 | **0.856** |
| **B** | **21** | -42.421 | **-12.05** | 7.556 | 13.179 | 0.32 | **0.838** |
| **A_A1** | **4** | -22.909 | **-6.829** | 4.655 | 7.288 | 0.34 | **0.829** |
| **B** | **65** | -48.847 | **-13.352** | 7.399 | 15.025 | 0.35 | **0.827** |
| **B** | **124** | -29.347 | **-8.486** | 5.128 | 9.152 | 0.35 | **0.825** |
| **B** | **95** | -32.246 | **-9.569** | 5.261 | 10.322 | 0.37 | **0.814** |
| **B** | **55** | -28.871 | **-7.691** | 6.144 | 9.158 | 0.38 | **0.812** |
| **B** | **40** | -30.584 | **-7.629** | 6.342 | 9.328 | 0.38 | **0.811** |
| **B** | **67** | -27.261 | **-7.437** | 5.541 | 8.586 | 0.38 | **0.809** |
| **B** | **154** | -33.428 | **-8.371** | 5.728 | 10.014 | 0.4 | **0.802** |
| A_A1 | 12 | -31.201 | -8.482 | 9.144 | 10.339 | 0.41 | 0.797 |
| CRF_02_AG | 1 | -31.595 | -7.776 | 4.762 | 9.686 | 0.41 | 0.794 |
| A_A1 | 5 | -33.496 | -9.282 | 7.993 | 10.967 | 0.42 | 0.792 |
| B | 85 | -26.688 | -6.685 | 6.180 | 8.374 | 0.42 | 0.792 |
| C | 25 | -28.909 | -6.874 | 7.062 | 9.247 | 0.45 | 0.777 |
| B | 22 | -36.056 | -8.832 | 7.905 | 11.307 | 0.46 | 0.77 |
| B | 34 | -11.156 | -2.635 | 3.877 | 3.775 | 0.47 | 0.766 |
| CRF_02_AG | 7 | -42.643 | -9.254 | 6.388 | 12.88 | 0.48 | 0.762 |
| B | 112 | -22.212 | -5.277 | 7.033 | 7.493 | 0.49 | 0.755 |
| B | 148 | -34.368 | -6.779 | 11.183 | 10.997 | 0.5 | 0.751 |
| B | 81 | -21.727 | -4.748 | 7.576 | 7.256 | 0.5 | 0.75 |
| B | 3 | -23.594 | -5.334 | 7.761 | 8.132 | 0.5 | 0.748 |
| B | 142 | -40.442 | -8.791 | 11.193 | 12.958 | 0.52 | 0.743 |
| B | 106 | -46.265 | -10.048 | 10.708 | 14.981 | 0.52 | 0.741 |
| B | 96 | -26.368 | -5.724 | 8.746 | 8.766 | 0.54 | 0.733 |
| A_A1 | 10 | -20.939 | -5.052 | 8.157 | 7.69 | 0.54 | 0.731 |
| B | 26 | -16.467 | -3.492 | 5.42 | 5.505 | 0.54 | 0.73 |
| B | 131 | -40.896 | -8.34 | 11.846 | 13.246 | 0.56 | 0.723 |
| C | 14 | -23.924 | -4.694 | 7.484 | 7.651 | 0.55 | 0.723 |
| C | 15 | -20.625 | -4.528 | 6.655 | 7.01 | 0.56 | 0.723 |
| B | 19 | -18.740 | -3.912 | 6.61 | 6.371 | 0.56 | 0.722 |
| B | 117 | -19.684 | -3.92 | 6.333 | 6.547 | 0.56 | 0.721 |
| B | 110 | -17.461 | -3.323 | 7.03 | 6.089 | 0.57 | 0.715 |
| A_A1 | 9 | -45.931 | -8.202 | 16.178 | 15.316 | 0.58 | 0.709 |
| B | 99 | -29.296 | -5.09 | 12.396 | 10.071 | 0.59 | 0.703 |
| B | 77 | -33.911 | -5.983 | 11.993 | 11.607 | 0.61 | 0.693 |
| B | 25 | -30.348 | -5.199 | 10.147 | 10.228 | 0.63 | 0.684 |
| C | 2 | -26.175 | -4.207 | 9.435 | 8.693 | 0.63 | 0.684 |
| B | 42 | -39.843 | -6.711 | 15.057 | 13.308 | 0.65 | 0.674 |
| B | 41 | -13.749 | -2.426 | 6.596 | 5.003 | 0.66 | 0.67 |
| B | 116 | -30.042 | -5.068 | 11.295 | 10.325 | 0.66 | 0.669 |
| A_A1 | 7 | -24.925 | -3.946 | 13.946 | 9.423 | 0.68 | 0.662 |
| B | 20 | -19.958 | -2.89 | 10.009 | 7.224 | 0.7 | 0.652 |
| B | 88 | -26.275 | -3.928 | 11.049 | 8.954 | 0.7 | 0.651 |
| B | 4 | -39.384 | -5.107 | 16.099 | 13.226 | 0.71 | 0.647 |
| C | 6 | -18.932 | -3.025 | 9.407 | 6.989 | 0.71 | 0.646 |
| B | 59 | -54.386 | -7.094 | 18.932 | 16.872 | 0.71 | 0.644 |
| B | 143 | -26.902 | -3.763 | 12.667 | 9.477 | 0.72 | 0.638 |
| B | 47 | -15.326 | -2.223 | 8.488 | 5.721 | 0.73 | 0.637 |
| C | 1 | -9.132 | -1.26 | 4.631 | 3.348 | 0.74 | 0.631 |
| C | 16 | -13.983 | -1.998 | 7.963 | 5.103 | 0.76 | 0.623 |
| C | 24 | -20.585 | -2.582 | 10.901 | 7.494 | 0.76 | 0.622 |
| B | 53 | -13.896 | -1.691 | 9.728 | 5.728 | 0.76 | 0.62 |
| B | 152 | -25.838 | -2.883 | 12.096 | 8.884 | 0.77 | 0.614 |
| B | 130 | -23.179 | -2.527 | 13.762 | 8.454 | 0.78 | 0.611 |
| B | 31 | -42.126 | -4.418 | 23.987 | 15.276 | 0.79 | 0.606 |
| B | 73 | -44.344 | -4.52 | 20.295 | 15.07 | 0.8 | 0.603 |
| B | 125 | -28.645 | -2.692 | 17.96 | 10.755 | 0.8 | 0.599 |
| B | 146 | -25.486 | -2.431 | 15.408 | 9.542 | 0.81 | 0.596 |
| C | 3 | -13.605 | -1.363 | 9.021 | 5.284 | 0.81 | 0.596 |
| B | 103 | -24.394 | -2.276 | 15.575 | 9.103 | 0.81 | 0.595 |
| B | 89 | -17.775 | -1.769 | 10.146 | 6.706 | 0.81 | 0.593 |
| B | 70 | -15.642 | -1.683 | 10.415 | 6.308 | 0.81 | 0.593 |
| B | 29 | -22.378 | -2.378 | 12.731 | 8.192 | 0.82 | 0.592 |
| B | 105 | -14.778 | -1.421 | 9.741 | 5.675 | 0.82 | 0.588 |
| CRF_02_AG | 3 | -21.382 | -1.986 | 14.388 | 8.506 | 0.83 | 0.584 |
| B | 75 | -35.936 | -3.25 | 18.163 | 13.543 | 0.84 | 0.578 |
| B | 7 | -32.207 | -2.403 | 21.833 | 11.782 | 0.85 | 0.575 |
| C | 18 | -30.792 | -2.626 | 18.104 | 11.882 | 0.87 | 0.565 |
| B | 121 | -13.219 | -0.857 | 9.82 | 5.474 | 0.87 | 0.565 |
| B | 134 | -20.599 | -1.487 | 14.301 | 8.379 | 0.88 | 0.558 |
| B | 51 | -13.185 | -0.857 | 11.055 | 5.829 | 0.89 | 0.555 |
| C | 28 | -14.999 | -0.835 | 12.833 | 6.461 | 0.89 | 0.553 |
| B | 136 | -25.344 | -1.712 | 18.765 | 10.552 | 0.9 | 0.552 |
| B | 101 | -22.418 | -1.198 | 16.974 | 8.905 | 0.9 | 0.551 |
| B | 138 | -24.776 | -1.775 | 14.492 | 8.908 | 0.9 | 0.55 |
| B | 94 | -26.550 | -1.307 | 20.396 | 10.531 | 0.91 | 0.547 |
| B | 79 | -23.201 | -1.442 | 19.131 | 10.356 | 0.91 | 0.545 |
| C | 13 | -20.546 | -0.985 | 17.429 | 8.745 | 0.91 | 0.545 |
| B | 122 | -23.474 | -1.537 | 18.308 | 9.739 | 0.91 | 0.543 |
| C | 30 | -15.117 | -0.78 | 14.411 | 6.758 | 0.92 | 0.54 |
| C | 20 | -19.440 | -0.684 | 15.712 | 8.067 | 0.92 | 0.54 |
| B | 141 | -22.704 | -0.749 | 19.981 | 9.84 | 0.92 | 0.538 |
| B | 108 | -17.185 | -0.65 | 14.754 | 7.59 | 0.94 | 0.531 |
| C | 5 | -33.239 | -1.481 | 24.109 | 12.893 | 0.94 | 0.528 |
| B | 11 | -14.664 | -0.31 | 13.311 | 6.399 | 0.96 | 0.522 |
| B | 32 | -23.089 | -0.562 | 19.225 | 10.012 | 0.96 | 0.519 |
| B | 104 | -16.331 | -0.41 | 14.065 | 7.137 | 0.97 | 0.516 |
| B | 135 | -14.099 | -0.166 | 13.47 | 6.384 | 0.98 | 0.511 |
| B | 123 | -17.136 | -0.507 | 13.748 | 7.399 | 0.98 | 0.509 |
| B | 91 | -23.784 | -0.062 | 23.607 | 10.948 | 0.98 | 0.509 |
| B | 46 | -29.672 | 0.058 | 33.27 | 15.334 | 0.98 | 0.509 |
| C | 8 | -11.373 | -0.055 | 10.176 | 5.147 | 0.99 | 0.503 |
| B | 61 | -11.911 | 0.2 | 11.564 | 5.699 | 1 | 0.501 |
| B | 107 | -29.502 | 0.329 | 30.133 | 13.872 | 1 | 0.499 |
| B | 18 | -18.114 | -0.305 | 15.314 | 7.592 | 1 | 0.498 |
| B | 145 | -13.012 | 0.096 | 14.587 | 6.294 | 1 | 0.498 |
| C | 17 | -6.705 | -0.108 | 5.92 | 2.947 | 0.98 | 0.492 |
| B | 93 | -16.214 | 0.089 | 16.006 | 7.447 | 0.98 | 0.492 |
| B | 86 | -15.099 | 0.198 | 16.81 | 7.637 | 0.98 | 0.492 |
| A_A1 | 2 | -20.304 | 0.561 | 22.66 | 9.71 | 0.96 | 0.481 |
| B | 78 | -13.857 | 0.845 | 19.225 | 7.542 | 0.96 | 0.478 |
| B | 30 | -24.003 | 1.128 | 29.397 | 12.401 | 0.95 | 0.475 |
| A_A1 | 1 | -40.744 | 1.213 | 42.228 | 18.404 | 0.95 | 0.475 |
| B | 38 | -17.566 | 0.343 | 19.308 | 8.782 | 0.95 | 0.473 |
| C | 9 | -11.445 | 0.561 | 13.608 | 6.151 | 0.92 | 0.458 |
| CRF_02_AG | 5 | -19.731 | 1.128 | 24.086 | 10.426 | 0.91 | 0.455 |
| B | 9 | -19.301 | 1.781 | 27.279 | 10.547 | 0.9 | 0.452 |
| B | 82 | -17.115 | 1.084 | 20.387 | 8.851 | 0.9 | 0.451 |
| CRF_02_AG | 8 | -15.688 | 1.477 | 21.758 | 8.732 | 0.9 | 0.451 |
| A_A1 | 6 | -20.099 | 1.436 | 25.017 | 10.161 | 0.9 | 0.449 |
| C | 7 | -16.833 | 1.05 | 21.082 | 8.723 | 0.9 | 0.448 |
| B | 126 | -12.437 | 1.16 | 16.309 | 6.768 | 0.89 | 0.445 |
| B | 100 | -22.129 | 1.23 | 23.183 | 10.543 | 0.88 | 0.442 |
| B | 115 | -15.470 | 1.454 | 21.316 | 8.492 | 0.88 | 0.441 |
| B | 119 | -15.531 | 1.868 | 23.987 | 9.261 | 0.88 | 0.438 |
| B | 13 | -23.042 | 1.888 | 31.892 | 12.298 | 0.87 | 0.437 |
| B | 52 | -9.208 | 1.175 | 13.613 | 5.661 | 0.87 | 0.436 |
| C | 10 | -23.186 | 2.897 | 33.065 | 13.161 | 0.86 | 0.431 |
| B | 98 | -12.913 | 1.4 | 18.711 | 7.315 | 0.86 | 0.429 |
| B | 23 | -12.806 | 2.494 | 23.907 | 8.758 | 0.85 | 0.427 |
| B | 63 | -17.658 | 2.76 | 31.465 | 10.948 | 0.85 | 0.426 |
| C | 19 | -12.285 | 2.005 | 21.133 | 7.628 | 0.84 | 0.42 |
| B | 72 | -9.715 | 1.163 | 13.188 | 5.602 | 0.83 | 0.414 |
| B | 2 | -15.721 | 2.363 | 24.158 | 9.433 | 0.82 | 0.412 |
| B | 111 | -18.206 | 3.373 | 34.544 | 12.722 | 0.82 | 0.412 |
| B | 102 | -11.656 | 1.661 | 19.016 | 7.311 | 0.82 | 0.411 |
| C | 4 | -9.327 | 1.074 | 11.508 | 4.933 | 0.82 | 0.408 |
| C | 11 | -7.371 | 1.342 | 12.014 | 4.621 | 0.81 | 0.404 |
| B | 113 | -13.753 | 2.95 | 28.071 | 9.943 | 0.8 | 0.401 |
| B | 68 | -16.811 | 3.466 | 30.825 | 11.615 | 0.8 | 0.399 |
| CRF_02_AG | 9 | -9.953 | 1.818 | 16.754 | 6.112 | 0.78 | 0.392 |
| B | 71 | -9.974 | 2.248 | 18.712 | 6.752 | 0.77 | 0.387 |
| B | 87 | -13.546 | 2.762 | 26.562 | 9.36 | 0.77 | 0.386 |
| B | 12 | -7.756 | 1.609 | 13.99 | 5.121 | 0.76 | 0.382 |
| B | 64 | -12.365 | 3.269 | 27.893 | 9.76 | 0.76 | 0.379 |
| B | 15 | -10.207 | 3.108 | 22.476 | 8.049 | 0.76 | 0.378 |
| C | 23 | -14.200 | 2.406 | 24.808 | 8.757 | 0.75 | 0.377 |
| C | 27 | -8.974 | 2.303 | 17.901 | 6.485 | 0.75 | 0.376 |
| CRF_02_AG | 11 | -12.932 | 4.247 | 31.548 | 10.672 | 0.75 | 0.376 |
| B | 14 | -11.322 | 2.561 | 20.076 | 7.472 | 0.74 | 0.371 |
| B | 128 | -14.270 | 4.307 | 32.645 | 11.63 | 0.74 | 0.368 |
| C | 26 | -11.197 | 3.134 | 25.202 | 8.361 | 0.73 | 0.367 |
| B | 120 | -10.890 | 2.735 | 20.573 | 7.546 | 0.72 | 0.361 |
| B | 114 | -11.009 | 3.126 | 23.166 | 8.001 | 0.71 | 0.356 |
| B | 35 | -9.516 | 2.711 | 18.242 | 6.718 | 0.71 | 0.355 |
| CRF_02_AG | 6 | -10.272 | 3.225 | 23.143 | 7.955 | 0.7 | 0.349 |
| B | 80 | -8.482 | 3.094 | 18.528 | 6.601 | 0.69 | 0.343 |
| B | 17 | -9.688 | 3.327 | 21.871 | 7.648 | 0.68 | 0.342 |
| B | 129 | -11.638 | 5.24 | 32.747 | 11.178 | 0.68 | 0.342 |
| B | 33 | -13.783 | 5.691 | 36.004 | 12.586 | 0.68 | 0.339 |
| B | 76 | -8.876 | 3.124 | 18.096 | 6.761 | 0.66 | 0.329 |
| B | 56 | -9.881 | 4.27 | 24.343 | 8.506 | 0.66 | 0.328 |
| B | 58 | -11.418 | 4.765 | 28.72 | 9.735 | 0.64 | 0.32 |
| CRF_02_AG | 2 | -8.985 | 3.960 | 22.078 | 7.756 | 0.62 | 0.311 |
| B | 149 | -8.563 | 4.324 | 24.212 | 8.012 | 0.62 | 0.309 |
| B | 66 | -10.820 | 6.025 | 34.762 | 11.043 | 0.6 | 0.302 |
| CRF_02_AG | 4 | -9.630 | 5.554 | 31.967 | 10.029 | 0.59 | 0.293 |
| B | 44 | -7.149 | 3.791 | 19.103 | 6.541 | 0.57 | 0.288 |
| B | 144 | -8.799 | 5.672 | 26.587 | 8.983 | 0.55 | 0.277 |
| B | 127 | -10.002 | 6.743 | 34.86 | 11.213 | 0.55 | 0.277 |
| B | 140 | -9.232 | 5.469 | 28.604 | 9.22 | 0.55 | 0.273 |
| B | 109 | -9.875 | 7.337 | 35.843 | 11.326 | 0.54 | 0.268 |
| B | 39 | -7.267 | 4.699 | 21.974 | 7.328 | 0.53 | 0.267 |
| B | 16 | -10.403 | 8.008 | 36.545 | 12.057 | 0.53 | 0.267 |
| B | 48 | -8.456 | 7 | 30.852 | 10.109 | 0.49 | 0.243 |
| B | 92 | -7.415 | 6.16 | 26.801 | 8.599 | 0.48 | 0.242 |
| B | 60 | -9.598 | 14.218 | 70.941 | 21.332 | 0.48 | 0.24 |
| B | 97 | -7.090 | 6.172 | 26.737 | 8.719 | 0.47 | 0.237 |
| B | 49 | -6.089 | 5.86 | 24.046 | 7.851 | 0.47 | 0.235 |
| B | 36 | -7.556 | 7.364 | 31.102 | 9.991 | 0.45 | 0.225 |
| B | 27 | -2.548 | 2.437 | 9.069 | 3.022 | 0.42 | 0.209 |
| B | 45 | -6.599 | 7.672 | 28.654 | 9.2 | 0.4 | 0.2 |
| B | 50 | -3.253 | 3.577 | 12.386 | 4.222 | 0.39 | 0.197 |
| B | 147 | -4.388 | 7.015 | 24.159 | 7.648 | 0.36 | 0.182 |
| B | 8 | -3.749 | 5.025 | 15.504 | 5.096 | 0.31 | 0.156 |
| C | 12 | -5.355 | 12.921 | 47.086 | 14.161 | 0.31 | 0.153 |
| B | 10 | -3.230 | 10.118 | 31.82 | 9.563 | 0.24 | 0.122 |
| B | 57 | 0.371 | 12.367 | 24.767 | 6.316 | 0.035 | 0.018 |
| B | 74 | 10.537 | 32.052 | 52.913 | 10.886 | 0.0017 | 0.001 |
| **Random Effect Variability and Correlation Structure** | | | | | |  |  |
| **Subtype** | **Parameter** | | **Lower 95% CI** | **Estimate** | **Upper 95% CI** |  |  |
| A_A1 | Individual Intercept SD | | 187.796 | 199.162 | 211.714 |  |  |
|  | Individual Slope SD | | 18.260 | 22.519 | 26.891 |  |  |
|  | Individual Intercept–Slope Correlation | | -0.685 | -0.568 | -0.431 |  |  |
|  | Residual Standard Deviation | | 99.023 | 103.167 | 107.805 |  |  |
| CRF_02_AG | Individual Intercept SD | | 200.135 | 212.757 | 227.034 |  |  |
|  | Individual Slope SD | | 27.301 | 33.6 | 40.269 |  |  |
|  | Individual Intercept–Slope Correlation | | -0.618 | -0.47 | -0.296 |  |  |
|  | Residual Standard Deviation | | 102.913 | 107.44 | 112.125 |  |  |
| C | Individual Intercept SD | | 184.51 | 190.752 | 197.969 |  |  |
|  | Individual Slope SD | | 20.152 | 22.312 | 24.708 |  |  |
|  | Individual Intercept–Slope Correlation | | -0.575 | -0.497 | -0.416 |  |  |
|  | Residual Standard Deviation | | 92.494 | 94.65 | 96.84 |  |  |

| B | Individual Intercept SD | 198.092 | 201.477 | 205.001 |  |  |
| --- | --- | --- | --- | --- | --- | --- |
|  | Individual Slope SD | 29.704 | 31.045 | 32.556 |  |  |
|  | Individual Intercept–Slope Correlation | -0.495 | -0.46 | -0.425 |  |  |
|  | Residual Standard Deviation | 113.154 | 114.384 | 115.695 |  |  |

### Table S11. Phylotype regression coefficients (effect on slope, *i.e.* rate of CD4 decline per year) from the Bayesian linear mixed-effects model of CD4 cell decline against the respective backbone phylotype on suspected VOIs highlighted by the ML CD4 decline model with a random effect on phylotypes (Table S8) and corroborated by the sensitivity analysis (Tables S9 and S10). In this model, phylotypes (estimated using *treestructure*, minimum clade size = 30) are modelled as having fixed effects on slopes and intercepts. Estimates are in cells/mm^3^/year units. The table is ordered by increasing Bayesian p-value and CD4 regression estimate (decline).

| **Subtype** | **Phylotype ID** | **Lower 95% CI** | **Regression coefficient (cells/mm^3^/year)** | **Upper 95% CI** | **Estimated error** | **Bayesian p-value** |
| --- | --- | --- | --- | --- | --- | --- |
| B | 133 | -54.075 | -31.861 | -9.053 | 11.124 | 0.0067 |
| B | 90 | -46.171 | -25.673 | -4.703 | 10.551 | 0.012 |
| A_A1 | 8 | -38.315 | -22.834 | -6.061 | 8.535 | 0.012 |
| A_A1 | 3 | -32.183 | -18.771 | -4.536 | 7.287 | 0.012 |
| B | 118 | -40.915 | -21.484 | -2.491 | 9.805 | 0.027 |
| B | 69 | -44.504 | -22.32 | -0.138 | 11.452 | 0.047 |
| B | 137 | -42.458 | -20.907 | 0.821 | 10.936 | 0.057 |
| B | 84 | -51.163 | -23.758 | 0.768 | 13.253 | 0.062 |
| B | 24 | -39.848 | -19.249 | 1.169 | 10.484 | 0.073 |
| B | 62 | -37.133 | -17.44 | 2.727 | 10.23 | 0.09 |
| B | 40 | -32.407 | -13.00 | 6.128 | 10.024 | 0.19 |
| CRF_02_AG | 7 | -44.911 | -16.22 | 12.861 | 15.128 | 0.28 |
| B | 77 | -41.656 | -12.706 | 13.073 | 13.979 | 0.38 |
| **Random Effect Variability and Correlation Structure** | | | | |  |  |
| **Subtype** | **Parameter** | | **Mean across pairwise comparisons (suspected VOI vs backbone)** | **SD across pairwise comparisons** | |  |
| A_A1 | Individual Intercept SD | | 197.547 | 0.332 | |  |
|  | Individual Slope SD | | 22.383 | 1.004 | |  |
|  | Individual Intercept–Slope Correlation | | -0.607 | 0.029 | |  |
|  | Residual Standard Deviation | | 98.369 | 2.431 | |  |
| B | Individual Intercept SD | | 204.552 | 0.171 | |  |
|  | Individual Slope SD | | 29.33 | 0.07 | |  |
|  | Individual Intercept–Slope Correlation | | -0.449 | 0.001 | |  |
|  | Residual Standard Deviation | | 113.738 | 0.08 | |  |
| CRF_02_AG | Individual Intercept SD | | 218.965 | NA (n=1) | |  |
|  | Individual Slope SD | | 34.548 |  |  |  |
|  | Individual Intercept–Slope Correlation | | -0.427 |  |  |  |
|  | Residual Standard Deviation | | 103.345 |  |  |  |

### Table S12. Absolute number and proportion of VOIs across the 24,100 subtype B sequences analysed in this study before and after (including) 2015. Values in parenthesis represent the percentage of each VOI across all analysed sequences in the respective period (before and after 2015). The total number of sequences before 2015 is 21,069 and after 2025 is 3031. We consider sequences from this year onwards to be of particular public health relevance due to their recency. A potential confounder is the change in recommendations for initiation of therapy as soon as diagnosed (irrespective of CD4 count) in 2015 (British HIV Association 2015).

| **Phylotype ID (subtype B)** | **Before 2015** | **After 2015** |
| --- | --- | --- |
| PT.B.40.UK | 73 (0.3) | 15 (0.5) |
| PT.B.69.UK | 59 (0.3) | 12 (0.4) |
| PT.B.133.UK | 33 (0.2) | 7 (0.2) |
| All VOI sequences | 165 (0.8) | 34 (1.1) |

###

### Table S13. Results of other covariates of the Bayesian fixed effects CD4 decline model for the six subtype B variants with significantly faster CD4 decline (see table S11). The phylotype coefficients from table S11 are repeated to facilitate comparison. Variable categories presented alone (*e.g.* age_group>60, phylotype84, and sexFemale) represent the effect of that grouping on the intercept (*i.e.* baseline CD4 count, in cells/mm^3^), while variables preceded by years_since_1cd4 (*e.g.* years_since_1cd4:phylotype133 and years_since_1cd4:sexFemale) represent the effect of that grouping on the slope (*i.e.* rate of CD4 decline per year, in cells/mm^3^/year). The reference category is: MSMs in the backbone phylotype in their thirties (30-39 age group). Regression coefficients are ranked in ascending order.

| **Subtype** | **Phylotype ID** | **Covariate** | **Lower 95% CI** | **Regression coefficient (cells/mm^3^/year)** | **Upper 95% CI** | **Estimated error** |
| --- | --- | --- | --- | --- | --- | --- |
| A_A1 | 3 | age_group>60 | -225.171 | -95.947 | 27.756 | 64.107 |
| B | 118 | age_group>60 | -136.934 | -86.335 | -32.978 | 26.43 |
| B | 90 | age_group>60 | -135.261 | -85.94 | -35.593 | 24.81 |
| B | 133 | age_group>60 | -131.977 | -85.317 | -37.401 | 24.966 |
| B | 69 | age_group>60 | -132.189 | -84.264 | -36.087 | 25.362 |
| A_A1 | 8 | age_group>60 | -190.118 | -70.636 | 41.844 | 59.314 |
| B | 90 | age_group50-59 | -77.668 | -54.437 | -27.994 | 12.585 |
| B | 69 | age_group50-59 | -78.818 | -54.376 | -28.872 | 12.605 |
| B | 133 | age_group50-59 | -78.412 | -54.227 | -29.542 | 12.445 |
| B | 118 | age_group50-59 | -78.868 | -53.818 | -29.314 | 12.386 |
| A_A1 | 3 | sexFemale | -75.68 | -43.403 | -8.484 | 17.616 |
| B | 118 | age_group40-49 | -49.821 | -35.38 | -20.917 | 7.61 |
| B | 90 | age_group40-49 | -49.212 | -35.162 | -20.833 | 7.261 |
| B | 133 | age_group40-49 | -48.921 | -34.57 | -19.644 | 7.689 |
| B | 69 | age_group40-49 | -48.892 | -34.111 | -18.359 | 7.769 |
| A_A1 | 3 | age_group50-59 | -109.925 | -33.405 | 37.359 | 35.638 |
| B | 90 | sexFemale | -57.923 | -32.897 | -7.156 | 12.822 |
| B | 118 | sexFemale | -58.256 | -32.673 | -6.814 | 13.691 |
| B | 133 | years_since_1cd4:phylotype133 | -54.075 | -31.861 | -9.053 | 11.124 |
| B | 69 | sexFemale | -55.788 | -31.706 | -6.913 | 12.65 |
| B | 133 | sexFemale | -56.27 | -31.509 | -5.724 | 12.839 |
| A_A1 | 8 | sexFemale | -63.948 | -29.829 | 5.181 | 17.32 |
| B | 90 | years_since_1cd4:phylotype90 | -46.171 | -25.673 | -4.703 | 10.551 |
| A_A1 | 8 | years_since_1cd4:phylotype8 | -38.315 | -22.834 | -6.061 | 8.535 |
| B | 69 | years_since_1cd4:phylotype69 | -44.504 | -22.32 | -0.138 | 11.452 |
| B | 118 | years_since_1cd4:phylotype118 | -40.915 | -21.484 | -2.491 | 9.805 |
| A_A1 | 3 | years_since_1cd4:exposureIDU | -47.244 | -20.282 | 7.647 | 14.191 |
| A_A1 | 3 | years_since_1cd4:phylotype3 | -32.183 | -18.771 | -4.536 | 7.287 |
| A_A1 | 8 | years_since_1cd4:exposureIDU | -36.472 | -15.3 | 6.191 | 11.058 |
| A_A1 | 8 | age_group50-59 | -82.009 | -13.484 | 59.706 | 36.386 |
| B | 118 | phylotype118 | -42.504 | -6.001 | 26.937 | 17.746 |
| B | 69 | phylotype69 | -40.66 | -5.779 | 31.752 | 18.019 |
| B | 133 | years_since_1cd4:sexFemale | -13.088 | -4.728 | 3.392 | 4.201 |
| B | 118 | years_since_1cd4:sexFemale | -12.542 | -4.653 | 3.327 | 4.12 |
| B | 90 | years_since_1cd4:sexFemale | -13.23 | -4.483 | 3.95 | 4.216 |
| B | 69 | years_since_1cd4:sexFemale | -12.874 | -4.471 | 3.749 | 4.218 |
| A_A1 | 3 | years_since_1cd4:exposureHeterosexual | -15.408 | -4.19 | 7.047 | 5.627 |
| A_A1 | 8 | years_since_1cd4:exposureHeterosexual | -12.019 | -1.159 | 9.481 | 5.402 |
| A_A1 | 8 | years_since_1cd4:sexFemale | -8.167 | -0.788 | 6.78 | 3.846 |
| A_A1 | 3 | years_since_1cd4:sexFemale | -8.975 | -0.736 | 7.762 | 4.249 |
| A_A1 | 3 | years_since_1cd4:exposureBloodproducts | -40.875 | -0.312 | 38.636 | 19.842 |
| A_A1 | 8 | years_since_1cd4:exposureBloodproducts | -37.515 | 0.265 | 37.964 | 19.21 |
| B | 133 | phylotype133 | -35.185 | 0.579 | 37.569 | 18.649 |
| B | 90 | years_since_1cd4:exposureIDU | -4.401 | 3.508 | 11.411 | 4.079 |
| B | 133 | years_since_1cd4:exposureIDU | -4.31 | 3.552 | 11.287 | 3.981 |
| B | 118 | years_since_1cd4:exposureIDU | -3.926 | 3.553 | 11.799 | 3.919 |
| B | 69 | years_since_1cd4:exposureIDU | -4.337 | 3.607 | 11.478 | 4.113 |
| B | 90 | phylotype90 | -31.685 | 3.875 | 41.789 | 18.847 |
| A_A1 | 8 | age_group40-49 | -39.978 | 6.077 | 49.065 | 22.807 |
| B | 69 | years_since_1cd4:exposureHeterosexual | 0.753 | 7.268 | 13.809 | 3.348 |
| B | 133 | years_since_1cd4:exposureHeterosexual | 1.006 | 7.517 | 14.192 | 3.375 |
| B | 90 | years_since_1cd4:exposureHeterosexual | 0.86 | 7.527 | 13.966 | 3.369 |
| B | 118 | years_since_1cd4:exposureHeterosexual | 1.326 | 7.773 | 14.127 | 3.316 |
| A_A1 | 3 | age_group40-49 | -34.258 | 8.368 | 53.152 | 21.541 |
| B | 69 | years_since_1cd4:exposureBloodproducts | -13.878 | 13.152 | 39.985 | 14.114 |
| B | 118 | years_since_1cd4:exposureBloodproducts | -16.259 | 13.361 | 42.418 | 14.528 |
| B | 133 | years_since_1cd4:exposureBloodproducts | -15.114 | 14.213 | 42.04 | 14.294 |
| B | 90 | years_since_1cd4:exposureBloodproducts | -12.957 | 14.27 | 41.974 | 14.25 |
| A_A1 | 8 | phylotype8 | -20.316 | 14.901 | 49.784 | 17.87 |
| B | 90 | age_group<29 | 14.096 | 26.333 | 38.954 | 6.413 |
| B | 133 | age_group<29 | 13.787 | 26.658 | 39.955 | 6.614 |
| B | 69 | age_group<29 | 15.242 | 26.73 | 39.609 | 6.304 |
| B | 118 | age_group<29 | 14.128 | 26.83 | 39.58 | 6.672 |
| A_A1 | 3 | phylotype3 | 6.687 | 41.371 | 75.871 | 17.223 |
| A_A1 | 8 | age_group<29 | 40.275 | 77.117 | 115.676 | 19.212 |
| A_A1 | 3 | age_group<29 | 39.394 | 77.135 | 112.406 | 19.104 |

###

### Table S14. Welch’s two-sided t-test comparing the mean age at diagnosis for individuals within VOI subtype B phylotypes against the backbone phylotype (ID=153). The only significant p-value (adjusted for multiple testing using the FDR method) and the associated VOI is highlighted in bold and underlined.

|  |  | **Age at diagnosis within phylotype** | | **Age at diagnosis in backbone phylotype** | |  |
| --- | --- | --- | --- | --- | --- | --- |
| **VOI phylotype ID (subtype B)** | **Observations (n)** | **Mean** | **SD** | **Mean** | **SD** | **p-value (FDR-adjusted)** |
| PT.B.40.UK | 86 | 34.105 | 10.102 | 36.241 | 10.388 | 0.081 |
| PT.B.69.UK | 70 | 35.414 | 10.480 |  |  | 0.51 |
| **PT.B.133.UK** | **39** | **30.333** | 8.637 |  |  | **0.00038** |

### Table S15. Major drug-resistance nucleotide mutation (and respective non-synonymous amino acid change) in high frequency (≥75%) for the VOI phylotypes. The number of non-VOI phylotypes having this mutation at ≥ 75% frequency is indicated.

|  |  | **VOI phylotype ID (subtype B)** | | | |
| --- | --- | --- | --- | --- | --- |
| **Pol nucleotide**  **(amino acid) mutation** | **Number of other phylotypes (total=154) with mutation at ≥ 75% frequency** | **40** | **69** | **133** | **153**  **(backbone)** |
| T268A (PR:L90M) | 0 | 86.36 |  |  |  |

### Table S16. Percentage of sequences within subtype B phylotypes with high and intermediate scores of drug-resistance (red and yellow, respectively) according to the Stanford University HIV Drug Resistance Database (HIVdb) searched using sierra-local for eight PR and 12 NRTI/NNRTI inhibitors. Percentages were included in the table only if present in ≥10% sequences of the phylotype at these two levels of resistance.

| **Phylotype ID**  **(subtype B)** | **PR inhibitors** | | | | | | | | **NRTIs / NNRTIs inhibitors** | | | | | | | | | | | |
| --- | --- | --- | --- | --- | --- | --- | --- | --- | --- | --- | --- | --- | --- | --- | --- | --- | --- | --- | --- | --- |
|  | **ATV** | **DRV** | **FPV** | **IDV** | **LPV** | **NFV** | **SQV** | **TPV** | **ABC** | **AZT** | **D4T** | **DDI** | **DOR** | **EFV** | **ETR** | **FTC** | **LMV** | **NVP** | **RPV** | **TDF** |
| 2 |  |  |  |  |  |  |  |  |  |  |  |  |  | 70.60 |  |  |  | 72.55 |  |  |
| 20 |  |  |  | 30.13 |  | 30.13 | 30.13 |  |  |  |  |  |  |  |  |  |  |  |  |  |
| 21 |  |  |  | 78.18 |  | 78.18 | 78.18 |  |  |  |  |  |  |  |  |  |  |  |  |  |
| 40ᵃ (VOI) |  |  |  | 86.36 |  | 87.50 | 86.36 |  |  |  |  |  |  |  |  |  |  |  |  |  |
| 43 |  |  |  |  |  |  |  |  |  |  |  |  |  | 11.92 |  |  |  | 11.92 |  |  |
| 55 |  |  |  |  |  |  |  |  |  |  |  |  |  | 100 |  |  |  | 100 |  |  |
| 57 |  |  |  |  |  |  |  |  |  |  |  |  |  | 25.48 |  |  |  | 25.48 |  |  |
| 69ᵇ (VOI) |  |  |  |  |  |  |  |  |  |  |  |  |  | 11.27 |  |  |  | 11.27 |  |  |
| 82 |  |  |  |  |  |  |  |  |  |  |  |  |  | 71.70 |  |  |  | 71.70 |  |  |
| 104 |  |  |  |  |  |  |  |  |  |  |  |  |  |  | 17.46 |  |  | 17.46 | 14.29 |  |
| 110 |  |  |  |  |  |  |  |  |  |  |  |  |  | 26.79 |  |  |  | 26.79 |  |  |
| 111 |  |  |  |  |  |  |  |  |  |  |  |  |  | 14.29 |  |  |  | 14.29 |  |  |
| 128 |  |  |  |  |  |  |  |  |  |  |  |  |  | 26.47 |  |  |  | 38.23 |  |  |
| 140 |  |  |  | 10.00 | 16.67 | 13.33 |  |  | 13.33 | 16.67 | 16.67 | 13.33 | 13.33 | 20.00 | 10.00 |  |  | 20.00 | 10.00 | 13.33 |
| 148 |  |  |  |  |  |  |  |  |  |  |  |  |  |  |  |  |  | 15.38 | 17.31 |  |
| 151 |  |  |  |  |  |  |  |  |  |  |  |  |  | 10.81 |  |  |  | 10.81 |  |  |

PI inhibitor abbreviations: atazanavir (ATV), darunavir (DRV), fosamprenavir (FPV), indinavir (IDV), lopinavir (LPV), nelfinavir (NFV), saquinavir (SQV), tipranavir (TPV).

NRTI/NNRTI inhibitors: abacavir (ABC), azidothymidine (AZT), stavudine (D4T), didanosine (DDI), emtricitabine (FTC), lamivudine (LMV), tenofovir disoproxil fumarate (TDF), doravirine (DOR), efavirenz (EFV), etravirine (ETR), nevirapine (NVP), and rilpivirine (RPV).

ᵃ The intermediate and high levels of resistance to IDV, NFV, and SQV in >86% of PT.B.40.UK sequences is mostly due to the PR:L90M DRM.

ᵇ The high-level of resistance to EFV and NVP in 11.27% of PT.B.69.UK sequences is mostly due to the RT:K103N DRM.

###

### Table S17. Bayesian model of CD4 post-treatment measurements with a fixed effect on phylotype. The key difference (when compared to the model described in Methods: Linkage between genetic data and CD4 counts and presented in Table S11) is that in this case we are investigating post-treatment CD4 increase (replenishment). Intercept represents the baseline CD4 in the reference category. Variable categories presented alone (*e.g.* age_group>60 and sexFemale) represent the effect of that grouping on the intercept (*i.e.* baseline CD4 count), while variables preceded by years_since_1cd4 (*e.g.* years_since_1cd4:VOI and years_since_1cd4:sexFemale) represent the effect of that grouping on the slope (*i.e.* rate of CD4 decline per year). Due to the limited sample sizes, we grouped all three VOIs into one category and compared it against the backbone. The table is ordered by decreasing regression coefficient.

| **Covariate** | **Lower 95% CI** | **Regression coefficient (cells/mm^3^/year)** | **Upper 95% CI** | **Estimated error** |
| --- | --- | --- | --- | --- |
| Intercept | 356.739 | 381.16 | 406.658 | 12.750 |
| years_since_1cd4 | 70.784 | 95.029 | 120.056 | 12.627 |
| years_since_1cd4:sexFemale | -30.171 | 69.215 | 166.61 | 49.728 |
| age_group0-29 | 28.786 | 68.686 | 110.287 | 21.266 |
| phylotypeVOI | -89.288 | 16.765 | 124.594 | 55.079 |
| years_since_1cd4:phylotypeVOI | -32.767 | 5.344 | 45.257 | 19.942 |
| years_since_1cd4:exposureHeterosexual | -94.649 | -28.187 | 54.700 | 36.932 |
| age_group40-49 | -69.401 | -30.388 | 8.670 | 19.696 |
| age_group50-59 | -90.430 | -41.64 | 9.840 | 26.631 |
| sexFemale | -119.179 | -63.844 | -9.711 | 27.041 |
| age_group>60 | -153.278 | -75.934 | -1.009 | 36.577 |

###

### Table S18. BLAST similarity search of subtype B VOI phylotypes against a reference database of 167,710 HIV-1 subtype B sequences from the LANL database. For each percent identity threshold ranging from 90 to 100%, the quantity of all global matches and non-UK-only matches are shown.

| **VOI phylotype ID (subtype B)** | **Total sequences in phylotype** | **All matches (including the UK)** | | | | | | | **Non-UK matches only** | | | | | | |
| --- | --- | --- | --- | --- | --- | --- | --- | --- | --- | --- | --- | --- | --- | --- | --- |
|  |  | **Percent identity threshold (%)** | | | | | | | | | | | | | |
|  |  | **90** | **95** | **96** | **97** | **98** | **99** | **100** | **90** | **95** | **96** | **97** | **98** | **99** | **100** |
|  |  |  |  |  |  |  |  |  |  |  |  |  |  |  |  |
| 40 | 88 | 3180 | 2372 | 526 | 51 | 49 | 36 | 21 | 3065 | 2280 | 475 | 8 | 8 | 6 | 1 |
| 69 | 71 | 3073 | 2807 | 1609 | 20 | 8 | 6 | 6 | 2991 | 2735 | 1556 | 11 | 0 | 0 | 0 |
| 133 | 40 | 2232 | 1967 | 1239 | 157 | 7 | 6 | 5 | 2199 | 1940 | 1220 | 149 | 0 | 0 | 0 |

##

## **References**

Altschul, S. F., Gish, W., Miller, W., Myers, E. W., & Lipman, D. J. (1990). ‘Basic local alignment search tool’, *Journal of Molecular Biology*, 215/3: 403–10. DOI: 10.1016/S0022-2836(05)80360-2

British HIV Association. (2015). ‘BHIVA guidelines for the treatment of HIV-1-positive adults with antiretroviral therapy 2015’. British HIV Association (BHIVA).

Carlson, J. M., Brumme, C. J., Martin, E., Listgarten, J., Brockman, M. A., Le, A. Q., Chui, C. K. S., et al. (2012). ‘Correlates of Protective Cellular Immunity Revealed by Analysis of Population-Level Immune Escape Pathways in HIV-1’, *Journal of Virology*, 86/24: 13202–16. American Society for Microbiology. DOI: 10.1128/jvi.01998-12

Connor, R. I., Sheridan, K. E., Ceradini, D., Choe, S., & Landau, N. R. (1997). ‘Change in Coreceptor Use Correlates with Disease Progression in HIV-1–Infected Individuals’, *The Journal of Experimental Medicine*, 185/4: 621–8.

Ho, J. C., Ng, G. T., Renaud, M., & Poon, A. F. y. (2019). ‘sierra-local: A lightweight standalone application for drug resistance prediction’, *Journal of Open Source Software*, 4/33: 1186. DOI: 10.21105/joss.01186

Jackson, B. (2022). ‘gofasta: command-line utilities for genomic epidemiology research’, *Bioinformatics*, 38/16: 4033–5. DOI: 10.1093/bioinformatics/btac424

Katoh, K., & Standley, D. M. (2013). ‘MAFFT Multiple Sequence Alignment Software Version 7: Improvements in Performance and Usability’, *Molecular Biology and Evolution*, 30/4: 772–80. Oxford Academic. DOI: 10.1093/molbev/mst010

Lengauer, T., Sander, O., Sierra, S., Thielen, A., & Kaiser, R. (2007). ‘Bioinformatics prediction of HIV coreceptor usage’, *Nature Biotechnology*, 25/12: 1407–10. Nature Publishing Group. DOI: 10.1038/nbt1371

Minh, B. Q., Schmidt, H. A., Chernomor, O., Schrempf, D., Woodhams, M. D., von Haeseler, A., & Lanfear, R. (2020). ‘IQ-TREE 2: New Models and Efficient Methods for Phylogenetic Inference in the Genomic Era’, *Molecular Biology and Evolution*, 37/5: 1530–4. DOI: 10.1093/molbev/msaa015

Price, M. N., Dehal, P. S., & Arkin, A. P. (2010). ‘FastTree 2 – Approximately Maximum-Likelihood Trees for Large Alignments’, *PLoS ONE*, 5/3: e9490. DOI: 10.1371/journal.pone.0009490

Shen, C., Park, M., & Warnow, T. (2022). ‘WITCH: Improved Multiple Sequence Alignment Through Weighted Consensus Hidden Markov Model Alignment’, *Journal of Computational Biology*, 29/8: 782–801. Mary Ann Liebert, Inc., publishers. DOI: 10.1089/cmb.2021.0585

Stanford HIV Drug Resistance Database (HIVdb). (2022). ‘PI Resistance Notes’. Retrieved February 15, 2024, from <https://hivdb.stanford.edu/dr-summary/resistance-notes/PI/>

——. (2024). *Major HIV-1 Drug Resistance Mutations*. Retrieved June 14, 2024, from <https://cms.hivdb.org/prod/downloads/resistance-mutation-handout/resistance-mutation-handout.pdf>

Tavare, S. (1986). ‘Some probabilistic and statistical problems in the analysis of DNA sequences’, *Some mathematical questions in biology / DNA sequence analysis edited by Robert M. Miura*. Providence, R.I. American Mathematical Society, c1986.

Vandekerckhove, L. P. R., Wensing, A. M. J., Kaiser, R., Brun-Vézinet, F., Clotet, B., Luca, A. D., Dressler, S., et al. (2011). ‘European guidelines on the clinical management of HIV-1 tropism testing’, *The Lancet Infectious Diseases*, 11/5: 394–407. Elsevier. DOI: 10.1016/S1473-3099(10)70319-4

Wheeler, T. J., & Eddy, S. R. (2013). ‘nhmmer: DNA homology search with profile HMMs’, *Bioinformatics*, 29/19: 2487–9. DOI: 10.1093/bioinformatics/btt403

Wymant, C., Bezemer, D., Blanquart, F., Ferretti, L., Gall, A., Hall, M., Golubchik, T., et al. (2022). ‘A highly virulent variant of HIV-1 circulating in the Netherlands’, *Science*, 375/6580: 540–5. American Association for the Advancement of Science. DOI: 10.1126/science.abk1688
