# Supplementary material for: Evidence for circulation of high-virulence HIV-1 subtype B variants in the United Kingdom
Source: Virus Evol. 2025 May 20;11(1):veaf048. doi: 10.1093/ve/veaf048 (PMC12257091; doi:10.1093/ve/veaf048)
Supplement: Data_S2_veaf048 [file data_s2_veaf048.pdf]

Data S2. UK HIV Drug Resistance Database and UK Collaborative HIV Cohort (UK CHIC) Steering committee members, Co-ordination, and Centres contributing data

A) UK HIV Drug Resistance Database

Steering Committee: David Asboe, Anton Pozniak (Chelsea & Westminster Hospital, London); Patricia Cane (Salisbury); David Chadwick (South Tees Hospitals NHS Trust, Middlesbrough); Duncan Churchill (Brighton and Sussex University Hospitals NHS Trust); Duncan Clark (Barts Health NHS Trust, London); Simon Collins (HIV i-Base, London); Valerie Delpech (National Infection Service, Public Health England); Samuel Douthwaite (Guy's and St. Thomas' NHS Foundation Trust, London); David Dunn, Esther Fearnhill, Kholoud Porter, Anna Tostevin, Oliver Stirrup (Institute for Global Health, UCL); Christophe Fraser (University of Oxford); Anna Maria Geretti (Institute of Infection and Global Health, University of Liverpool); Rory Gunson (Gartnavel General Hospital, Glasgow); Antony Hale (Leeds Teaching Hospitals NHS Trust); Stéphane Hué (London School of Hygiene and Tropical Medicine); Steve Kaye (Imperial College, London); Linda Lazarus (Expert Advisory Group on AIDS Secretariat, Public Health England); Andrew Leigh-Brown (University of Edinburgh); Tamyo Mbisa (National Infection Service, Public Health England); Nicola Mackie (Imperial NHS Trust, London); Samuel Moses (King's College Hospital, London); Chloe Orkin (Barts Health NHS Trust, London); Eleni Nastouli, Deenan Pillay, Andrew Phillips, Caroline Sabin (University College London, London); Erasmus Smit (Public Health England, Birmingham Heartlands Hospital); Kate Templeton (Royal Infirmary of Edinburgh); Peter Tilston (Manchester Royal Infirmary); Ian Williams (Mortimer Market Centre, London); Hongyi Zhang (Addenbrooke's Hospital, Cambridge)

Central Co-ordination: Institute for Global Health, UCL (David Dunn, Keith Fairbrother, Esther Fearnhill, Kholoud Porter, Anna Tostevin, Oliver Stirrup)

Centres contributing data: Clinical Microbiology and Public Health Laboratory, Addenbrooke's Hospital, Cambridge (Jane Greatorex); Guy's and St Thomas' NHS Foundation Trust, London (Siobhan O'Shea, Jane Mullen); PHE – Public Health Laboratory, Birmingham Heartlands Hospital, Birmingham (Erasmus Smit); Antiviral Unit, National Infection Service, Public Health England, London (Tamyo Mbisa); Imperial College Health NHS Trust, London (Alison Cox); King's College Hospital, London (Richard Tandy); Medical Microbiology Laboratory, Leeds Teaching Hospitals NHS Trust (Tracy Fawcett); Specialist Virology Centre, Liverpool (Mark Hopkins); Department of Clinical Virology, Manchester Royal Infirmary, Manchester (Peter Tilston); Department of Virology, Royal Free Hospital, London (Clare Booth, Ana Garcia-Diaz); Edinburgh Specialist Virology Centre, Royal Infirmary of Edinburgh (Lynne Renwick); Department of Infection & Tropical Medicine, Royal Victoria Infirmary, Newcastle (Matthias L Schmid, Brendan Payne); South Tees Hospitals NHS Trust, Middlesbrough (David Chadwick); Department of Virology, Barts Health NHS Trust, London (Jonathan Hubb); Molecular Diagnostic Unit, Imperial College, London (Steve Kaye); University College London Hospitals (Stuart Kirk); West of Scotland Specialist Virology Laboratory, Gartnavel, Glasgow (Rory Gunson, Amanda Bradley-Stewart)

B) UK Collaborative HIV Cohort (UK CHIC)

Steering Committee: Jonathan Ainsworth, Sris Allan, Jane Anderson, Ade Apoola, David Chadwick, Duncan Churchill, Valerie Delpech, David Dunn, Ian Fairley, Ashini Fox, Richard Gilson, Mark Gompels, Phillip Hay, Rajesh Hembrom, Teresa Hill, Margaret Johnson, Sophie Jose, Stephen Kegg, Clifford Leen, Dushyant Mital, Mark Nelson, Hajra Okhai, Chloe Orkin, Adrian Palfreeman, Andrew Phillips, Deenan Pillay, Ashley Price, Frank Post, Jillian Pritchard, Caroline Sabin, Achim Schwenk, Anjum Tariq, Roy Trevelion, Andy Ustianowski, John Walsh.

Central Co-ordination: University College London (David Dunn, Teresa Hill, Hajra Okhai, Andrew Phillips, Caroline Sabin); Medical Research Council Clinical Trials Unit at UCL (MRC CTU at UCL), London (Nadine van Looy, Keith Fairbrother).

Participating Centres: Barts Health NHS Trust, London (Chloe Orkin, Janet Lynch, James Hand); Brighton and Sussex University Hospitals NHS Trust (Duncan Churchill, Stuart Tilbury, Elaney Youssef, Duncan Churchill); Chelsea and Westminster Hospital NHS Foundation Trust, London (Mark Nelson, Richard Daly, David Asboe, Sundhiya Mandalia); Homerton University Hospital NHS Trust, London (Jane Anderson, Sajid Munshi); King's College Hospital NHS Foundation Trust, London (Frank Post, Ade Adefisan, Chris Taylor, Zachary Gleisner, Fowzia Ibrahim, Lucy Campbell); Middlesbrough, South Tees Hospitals NHS Foundation Trust, (David Chadwick, Kirsty Baillie); Mortimer Market Centre, University College London (Richard Gilson, Ian Williams); North Middlesex University Hospital NHS Trust, London (Jonathan Ainsworth, Achim Schwenk, Sheila Miller, Chris Wood); Royal Free NHS Foundation Trust/University College London (Margaret Johnson, Mike Youle, Fiona Lampe, Colette Smith, Rob Tsintas, Clinton Chaloner, Caroline Sabin, Andrew Phillips, Teresa Hill, Hajra Okhai); Imperial College Healthcare NHS Trust, London (John Walsh, Nicky Mackie, Alan Winston, Jonathan Weber, Farhan Ramzan, Mark Carder); The Lothian University Hospitals NHS Trust, Edinburgh (Clifford Leen, Andrew Kerr, David Wilks, Sheila Morris); North Bristol NHS Trust (Mark Gompels, Sue Allan); Leicester, University Hospitals of Leicester NHS Trust (Adrian Palfreeman, Adam Lewszuk); Woolwich, Lewisham and Greenwich NHS Trust (Stephen Kegg, Victoria Ogunbiyi, Sue Mitchell), St. George's Healthcare NHS Trust (Phillip Hay, Christopher Hunt, Olanike Okolo, Benjamin Watts); York Teaching Hospital NHS Foundation Trust (Ian Fairley, Sarah Russell-Sharpe, Olatunde Fagbayimu); Coventry, University Hospitals Coventry and Warwickshire NHS Trust (Sris Allan, Debra Brain); Wolverhampton, The Royal Wolverhampton Hospitals NHS Trust (Anjum Tariq, Liz Radford, Sarah Milgate); Chertsey, Ashford and St.Peter's Hospitals NHS Foundation Trust (Jillian Pritchard, Shirley Cumming, Claire Atkinson); Milton Keynes Hospital NHS Foundation Trust (Dushyant Mital, Annie Rose, Jeanette Smith); The Pennine Acute Hospitals NHS Trust (Andy Ustianowski, Cynthia Murphy, Ilise Gunder); Nottingham University Hospitals NHS Trust (Ashini Fox, Howard Gees, Gemma Squires, Laura Anderson), Kent Community Health NHS Foundation Trust (Rajesh Hembrom, Serena Mansfield, Lee Tomlinson, Christine LeHegerat, Roberta Box, Tom Hatton, Doreen Herbert), The Newcastle upon Tyne Hospitals NHS Foundation Trust (Ashley Price, Ian McVittie, Victoria Murtha, Laura Shewan); Derby Teaching Hospitals NHS Foundation Trust (Ade Apoola, Zak Connan, Luke Gregory, Kathleen Holding, Victoria Chester, Trusha Mistry, Catherine Gatford); UK Health Security Agency, London (Valerie Delpech); i-Base (Roy Trevelion)
